# Trans-ethnic Fine Mapping Highlights Kidney-Function Genes Linked to Salt Sensitivity

**DOI:** 10.1016/j.ajhg.2016.07.012

**Published:** 2016-09-01

**Authors:** Anubha Mahajan, Aylin R. Rodan, Thu H. Le, Kyle J. Gaulton, Jeffrey Haessler, Adrienne M. Stilp, Yoichiro Kamatani, Gu Zhu, Tamar Sofer, Sanjana Puri, Jeffrey N. Schellinger, Pei-Lun Chu, Sylvia Cechova, Natalie van Zuydam, the SUMMIT Consortium, the BioBank Japan Project, Johan Arnlov, Michael F. Flessner, Vilmantas Giedraitis, Andrew C. Heath, Michiaki Kubo, Anders Larsson, Cecilia M. Lindgren, Pamela A.F. Madden, Grant W. Montgomery, George J. Papanicolaou, Alex P. Reiner, Johan Sundström, Timothy A. Thornton, Lars Lind, Erik Ingelsson, Jianwen Cai, Nicholas G. Martin, Charles Kooperberg, Koichi Matsuda, John B. Whitfield, Yukinori Okada, Cathy C. Laurie, Andrew P. Morris, Nora Franceschini

**Affiliations:** 1 Wellcome Trust Centre for Human Genetics, University of Oxford, Oxford OX3 7BN, UK; 2 Department of Internal Medicine, University of Texas Southwestern Medical Center, Dallas, TX 75229, USA; 3 Department of Medicine, University of Virginia, Charlottesville, VA 22908, USA; 4 Department of Pediatrics, University of California San Diego, La Jolla, CA 92093, USA; 5 Division of Public Health Sciences, Fred Hutchinson Cancer Research Center, Seattle, WA 98109, USA; 6 Department of Biostatistics, University of Washington, Seattle, WA 98195, USA; 7 Laboratory for Statistical Analysis, RIKEN Center for Integrative Medical Sciences, Yokohama 230-0045, Japan; 8 Genetic Epidemiology Laboratory, QIMR Berghofer Medical Research Institute, Brisbane 4006, Australia; 9 Department of Medical Sciences, Cardiovascular Epidemiology, Uppsala University, Uppsala 751 85, Sweden; 10 School of Health and Social Studies, Dalarna University, Falun 791 88, Sweden; 11 National Institute of Diabetes, Digestive, and Kidney Disease, NIH, Bethesda, MD 20892, USA; 12 Department of Public Health and Caring Sciences, Molecular Geriatrics, Uppsala University, Uppsala 752 37, Sweden; 13 Department of Psychiatry, Washington University in St. Louis, St. Louis, MO 63110, USA; 14 Laboratory for Genotyping Development, RIKEN Center for Integrative Medical Sciences, Yokohama 230-0045, Japan; 15 Big Data Institute, Li Ka Shing Centre for Health Information and Discovery, University of Oxford, Oxford OX3 7BN, UK; 16 Molecular Epidemiology Laboratory, QIMR Berghofer Medical Research Institute, Brisbane 4006, Australia; 17 Epidemiology Branch, Division of Cardiovascular Sciences, National Heart, Lung and Blood Institute, Bethesda, MD 20892, USA; 18 Department of Medical Sciences, Molecular Epidemiology and Science for Life Laboratory, Uppsala University, Uppsala 752 37, Sweden; 19 Department of Medicine, Division of Cardiovascular Medicine, Stanford University School of Medicine, Stanford, CA 94305, USA; 20 Collaborative Studies Coordinating Center, Department of Biostatistics, University of North Carolina at Chapel Hill, Chapel Hill, NC 27599, USA; 21 Laboratory of Molecular Medicine, Human Genome Center, Institute of Medical Science, University of Tokyo, Tokyo 108-8639, Japan; 22 Department of Statistical Genetics, Osaka University Graduate School of Medicine, Osaka 565-0871, Japan; 23 Department of Biostatistics, University of Liverpool, Liverpool L69 3GL, UK; 24 Department of Epidemiology, University of North Carolina, Chapel Hill, NC 27514, USA

## Abstract

We analyzed genome-wide association studies (GWASs), including data from 71,638 individuals from four ancestries, for estimated glomerular filtration rate (eGFR), a measure of kidney function used to define chronic kidney disease (CKD). We identified 20 loci attaining genome-wide-significant evidence of association (p < 5 × 10^−8^) with kidney function and highlighted that allelic effects on eGFR at lead SNPs are homogeneous across ancestries. We leveraged differences in the pattern of linkage disequilibrium between diverse populations to fine-map the 20 loci through construction of “credible sets” of variants driving eGFR association signals. Credible variants at the 20 eGFR loci were enriched for DNase I hypersensitivity sites (DHSs) in human kidney cells. DHS credible variants were expression quantitative trait loci for *NFATC1* and *RGS14* (at the *SLC34A1* locus) in multiple tissues. Loss-of-function mutations in ancestral orthologs of both genes in *Drosophila melanogaster* were associated with altered sensitivity to salt stress. Renal mRNA expression of *Nfatc1* and *Rgs14* in a salt-sensitive mouse model was also reduced after exposure to a high-salt diet or induced CKD. Our study (1) demonstrates the utility of trans-ethnic fine mapping through integration of GWASs involving diverse populations with genomic annotation from relevant tissues to define molecular mechanisms by which association signals exert their effect and (2) suggests that salt sensitivity might be an important marker for biological processes that affect kidney function and CKD in humans.

## Introduction

Chronic kidney disease (CKD) is a major public health burden and affects nearly 10% of the global population.^1^ Reduced estimated glomerular filtration rate (eGFR), a measure of kidney function used to define CKD, is associated with premature cardiovascular disease and mortality, acute kidney injury, and progression to end stage renal disease (ESRD).^2^ Although individuals of African and Hispanic descent suffer the largest burden of CKD,^3^ the largest genome-wide association studies (GWASs) to search for kidney-function loci have been undertaken in populations of European and East Asian ancestry.^4^, ^5^, ^6^, ^7^, ^8^ Many of these loci are characterized by common variant association signals that map to large genomic intervals, which contain many possible causal genes for eGFR, thereby limiting understanding of the downstream pathogenesis of CKD.

To address this challenge, we have undertaken a trans-ethnic meta-analysis of nine GWASs comprising 71,638 individuals from four ancestries (African American, Hispanic, European, and East Asian), each imputed up to the phase 1 integrated (March 2012 release) multi-ethnic reference panel from the 1000 Genomes Project^9^, from the Continental Origins and Genetic Epidemiology Network (COGENT)-Kidney consortium. With these data, we aimed to (1) assess the evidence for heterogeneity in allelic effects on eGFR for lead SNPs at kidney-function loci across ethnic groups; (2) fine-map these loci by taking advantage of high-density imputation and by leveraging differences in the pattern of linkage disequilibrium (LD) between diverse populations to localize “credible sets” of variants driving eGFR association signals; (3) define potential molecular mechanisms through which eGFR association signals at these loci impact kidney function through overlap of credible variants with genomic annotation; and (4) assess possible markers for biological processes that impact kidney function and CKD in humans through targeted experimentation in model organisms.

## Subjects and Methods

### Ethics Statement

All human research was approved by the relevant institutional review boards and conducted according to the Declaration of Helsinki. All participants provided written informed consent.

### Study Overview

We aggregated five GWASs of individuals of European ancestry (23,553 individuals from Europe, the USA, and Australia), two GWASs of Hispanic Americans (16,325 individuals from the USA), one GWAS of individuals of East Asian ancestry (23,536 individuals from Japan), and one GWAS of African Americans (8,224 individuals from the USA). Study sample characteristics are presented in Table S1.

### Genotyping, Quality Control, and Imputation

Samples were genotyped with a variety of GWAS arrays, and quality control was undertaken within each study (Table S2). Sample quality control included exclusions on the basis of genome-wide call rate, extreme heterozygosity, sex discordance, cryptic relatedness, and outlying ethnicity. SNP quality control included exclusions on the basis of call rate across samples and extreme deviation from Hardy-Weinberg equilibrium. Non-autosomal SNPs were excluded from imputation and association analysis.

Within each study, the autosomal GWAS genotype scaffold was first pre-phased^10^, ^11^ with genetic maps from the International HapMap Consortium^12^ to model recombination rates. The scaffold was then imputed up to the phase 1 integrated (March 2012 release) multi-ethnic reference panel from the 1000 Genomes Project^9^ via IMPUTE2^11^, ^13^ or MaCH/Minimac^11^ (Table S2). Imputed variants were retained for downstream association analyses if they attained established GWAS quality control thresholds:^14^ IMPUTE2 info ≥ 0.4 or MaCH/Minimac *r*^2^ ≥ 0.3.

### Calculation of eGFR and Association Analysis

Within each study, eGFR was calculated from serum creatinine (mg/dL), with adjustment for age, sex, and ethnicity by means of the four-variable MDRD (modification of diet in renal disease) equation^15^ to be comparable with published GWASs of kidney function.^4^, ^5^, ^6^, ^7^, ^8^ Within each study, we tested association of eGFR with each variant passing quality control in a linear regression framework under an additive dosage model and with adjustment for study-specific covariates to account for confounding due to population structure (Table S2). Association summary statistics were subsequently corrected in each study for residual population structure through a first round of genomic control^16^ where necessary (Table S2).

### Trans-ethnic Meta-analysis

Association summary statistics were combined across studies via fixed-effects meta-analysis (inverse-variance weighting) implemented in the GWAMA software.^17^ Variants passing quality control in fewer than 50% of the total sample size across studies were excluded from the meta-analysis. Association summary statistics from the meta-analysis were then corrected for a second round of genomic control^16^ (λ_GC_ = 1.028). Heterogeneity in allelic effects between studies at each variant was assessed by means of Cochran’s Q statistic.^18^ We extracted association summary statistics for eGFR from the trans-ethnic meta-analysis for previously reported lead SNPs at established GWAS loci.

### LD Calculations

LD, as measured by the correlation coefficient *r*^2^, was calculated on the basis of haplotypes in each ancestry group from the 1000 Genomes Project^9^ via LDlink.^19^

### Conditional Analyses

To assess the evidence for distinct association signals at each locus attaining nominal significance (p_COND_ < 10^−5^, Bonferroni correction for ∼5,000 variants per locus) in our trans-ethnic meta-analysis, we performed conditional analysis in a 1 Mb genomic interval flanking the lead SNP. Within each study, we tested association of eGFR with each variant passing quality control in the flanking region in a linear regression framework under an additive dosage model and with adjustment for genotypes at the lead SNP, in addition to other study-specific covariates used in unconditional analysis (Table S2). Association summary statistics were subsequently corrected in each study for residual population structure, via the same genomic control^16^ correction employed for unconditional analysis (Table S2). These association summary statistics were combined across studies via fixed-effects meta-analysis (inverse-variance weighting) implemented in GWAMA.^17^ Variants passing quality control in less than 50% of the total sample size across studies were excluded from the meta-analysis. Association summary statistics from the conditional meta-analysis were then corrected for a second round of genomic control,^16^ making use of the same adjustment as defined in the unconditional analysis (λ_GC_ = 1.028).

### Association with CKD

We defined CKD by an eGFR < 60 mL/min/1.73 m^2^ (calculated with the MDRD equation defined above) and/or incidence of ESRD, if available. Any individual who was prospectively initiated on dialysis or received a kidney transplant (self-reported or obtained from medical records or registries) was defined as having ESRD. Individuals who did not develop ESRD at follow-up were considered control subjects. We considered the lead eGFR SNP identified at each locus attaining genome-wide significance in our trans-ethnic meta-analysis. Within each study, we tested association of CKD with each SNP in a logistic regression framework under an additive dosage model and with adjustment for study-specific covariates to account for confounding due to population structure (Table S2). Association summary statistics were combined across studies via fixed-effects meta-analysis (sample size and inverse-variance weighting) implemented in METAL^20^ and GWAMA.^17^

### Association with eGFR in Diabetic Individuals from the SUMMIT Consortium

We considered the lead eGFR SNP at each locus attaining genome-wide significance in our trans-ethnic meta-analysis. We performed a look-up of association summary statistics for eGFR in 13,158 subjects with diabetes (9,197 with type 2 diabetes [T2D] and 3,961 with type 1 diabetes [T1D]) from five studies of individuals of European ancestry from the SUMMIT Consortium. Within each study, the outcome variable was defined as the last measured eGFR, calculated with the MDRD equation (defined above). Each study was imputed up to the phase 1 integrated (March 2012 release) multi-ethnic reference panel from the 1000 Genomes Project.^9^ Estimated allelic effects on eGFR were obtained from a linear mixed model and implemented in EMMAX^21^ with an empirical genetic relationship matrix, assuming an additive dosage of the minor allele and including sex, age at diabetes onset, and duration of diabetes as covariates. Association summary statistics for eGFR were combined across studies via fixed-effects meta-analysis (inverse-variance weighting) implemented in GWAMA.^17^ Combined allelic effect estimates across studies were reported for T1D and T2D subjects, both separately and for all diabetic individuals combined. Heterogeneity in allelic effects between T1D and T2D subjects at each variant was assessed by means of Cochran’s Q statistic,^18^ as implemented in GWAMA.^17^

For lead SNPs, we tested for a difference in the allelic effect on eGFR in the general population (from our trans-ethnic meta-analysis) and in diabetic indivuduals (combined T1D and T2D from the SUMMIT Consortium) by using a two-sample Z-test.

### MANTRA Fine Mapping and Credible Set Construction

We performed trans-ethnic fine mapping of each locus in a 1 Mb genomic interval flanking the lead SNP. Association summary statistics for each variant in the flanking region were combined across studies with a Bayesian hybrid of fixed- and random-effects meta-analysis, as implemented in MANTRA.^22^ MANTRA allows for heterogeneity in allelic effects between ancestry groups arising as a result of differences in the structure of LD between diverse populations by assigning studies to clusters according to a Bayesian partition model of relatedness between them, defined by pairwise genome-wide mean allele frequency differences (Figure S1). MANTRA has been demonstrated, both empirically and by simulation, to improve fine-mapping resolution, as compared to either a fixed- or random-effects meta-analysis.^22^, ^23^, ^24^ Variants passing quality control in less than 50% of the total sample size across studies were excluded from the fine-mapping analysis.

We calculated the posterior probability that the *j*^th^ variant, π_C*j*_, is driving the association signal at each locus by

$$\pi_{Cj}=\frac{\Lambda_{j}}{\sum_{k}\Lambda_{k}},$$

where the summation is over all variants in the flanking interval. In this expression, *Λ*_*j*_ is the MANTRA Bayes factor in favor of association from the trans-ethnic meta-analysis. For each distinct association signal, a 99% credible set^25^ was then constructed by (1) ranking all variants according to their Bayes factor, *Λ*_*j*_, and (2) including ranked variants until their cumulative posterior probability exceeds 0.99.

### Genomic Annotation

For each locus attaining genome-wide significance in our trans-ethnic meta-analysis, we obtained genomic annotations of all single-nucleotide variants in a 1 Mb interval flanking the lead SNP. We utilized the Ensembl Variant Effect Predictor (VEP, version 2.7), based on the Ensembl transcript set (version 69). By default, the VEP reports all possible annotations (transcript- and gene-specific) for each variant. We therefore prioritized annotations by considering the most severe consequence of all those reported. We then calculated the total posterior probability of driving association signals for each consequence across loci.

### Regulatory Annotation

We collected genomic annotations from three sources. First, we obtained regulatory chromatin states from the Epigenome Roadmap Project^26^ for 93 cell types after removing five cancer cell lines. For each cell type, we pooled enhancer (EnhA and EnhWk) and promoter (TssA and TssFlnk) elements into one annotation. Second, we obtained 145 non-redundant DNase I hypersensitivity sites (DHSs) from the ENCODE Project^27^ by retaining only one dataset for cell types with multiple assayed samples. Third, we obtained chromatin immuno-precipitation sequence (ChIP-seq) binding sites for 165 transcription factors: 161 proteins from the ENCODE Project^27^ and additional factors assayed in primary pancreatic islets.^28^ This resulted in a total of 403 annotations for downstream enrichment analyses. For each annotation, we considered variants passing quality control and mapping within 1 Mb of the lead SNP attaining genome-wide significance in the trans-ethnic meta-analysis.

We first tested the effect of each annotation on the log odds of the posterior probability of driving eGFR association signals in a logistic regression model. For each variant, we encoded overlap with the tested annotation as a binary indicator (1 if variant overlaps annotation, 0 otherwise). The regression model also incorporated binary indicators of genic annotations as covariates, as well as a categorical variable for locus membership. Specifically,

$$\text{logit}\left(\pi_{\text{C}j}\right)=\alpha_{i}L_{ij}+\beta_{k}x_{jk}+\gamma_{3^{\prime }\mathrm{UTR}}x_{j3^{\prime }\mathrm{UTR}}+\gamma_{5^{\prime }\mathrm{UTR}}x_{j5^{\prime }\mathrm{UTR}}+\gamma_{\text{EXON}}x_{j\text{EXON}}+\gamma_{\mathrm{TSS}}x_{j\mathrm{TSS}},$$

where *π*_*Cj*_ is the posterior probability that the *j*^th^ variant drives the eGFR association; *α*_*i*_ denotes an intercept for the *i*^th^ locus and *L*_*ij*_ is a binary indicator of membership of the *j*^th^ variant in the *i*^th^ locus; *β*_*k*_ denotes the effect of the *k*^th^ annotation and *x*_*kj*_ is a binary indicator of overlap of the *j*^th^ variant with the *k*^th^ annotation; and *γ*_3′__UTR_, *γ*_5′_ _UTR_, *γ*_EXON_ and *γ*_TSS_ denote the effects of 3′ UTRs, 5′ UTRs, coding exons, and the region within 1 kb upstream of GENCODE transcription start site (TSS) annotations, respectively, and *x*_*j*3′__UTR_, *x*_*j*5′__UTR_, *x*_*j*EXON_ and *x*_*j*TSS_ are binary indicators of overlap of the *j*^th^ variant with these annotations. The SE of the effect of the *k*^th^ annotation, *β*_*k*_, was evaluated with a robust sandwich variance estimator.

Using fGWAS software, we then tested for the effect of each annotation by using the Bayes factor in favor of association.^29^ We included coding exons, 3′ UTRs, 5′ UTRs, and the region within 1 kb upstream of the TSS in the model for each annotation. We obtained the estimated effect and 95% confidence interval (CI) from this model and considered an annotation enriched if the 95% CI did not overlap zero.

### *Drosophila melanogaster* Salt-Sensitivity Assay

Four *y*^*1*^*w*^*1*^ virgin females were mated with two males each of the genotypes *y*^*1*^*w*^*1*^*/Y, y*^*1*^*w*^*1*^*/Y*; *loco*^*EY-P283*^*/TM3 Sb* or *y*^*1*^*w*^*1*^*/Y*; *loco*^*d06164*^ in rearing vials on standard cornmeal/yeast/molasses food (prepared in a central kitchen at University of Texas Southwestern Medical Center). The *y*^1^*w*^1^ isogenic control, in which all loci had been previously homozygosed, and to which *loco* mutants had been backcrossed for six generations, were obtained from Dr. Yongkyu Park (Rutgers New Jersey Medical School).^30^ Separately, to obtain highly heterozygous progeny (heterogenic), virgin females from the A.R.R. lab’s *wBerlin* strain were mated with males of genotypes *y*^*1*^*w*^*1*^*/Y*, *y*^*1*^*w*^*1*^*/Y*; *loco*^*EY-P283*^*/TM3 Sb* or *y*^*1*^*w*^*1*^*/Y*; *loco*^*d06164*^, as above. Adults were cleared from rearing vials on rearing day eight. Ten female progeny from each vial were collected within 1–3 days of eclosion and placed on food containing various concentrations of added NaCl. Each experimental vial contained flies from a single rearing vial. The number of dead flies in each vial was counted daily. Flies were transferred to fresh medium after day five, and again after day ten for the heterogenic flies. For each concentration of experimental medium, 225 g Applied Scientific Jazz-Mix *Drosophila* Food (Fisher, cat. no. AS-153) was added to 500 mL deionized water with constant stirring. Flasks were then placed on a hot plate at 350°C with constant stirring and heated to a slow boil (about 20–25 min). The heat was then turned off, 4M NaCl was added to achieve varying concentrations of added NaCl, and total volume adjusted to 900 mL with deinonized water. Medium was dispensed in 3–4 mL aliquots in polystyrene vials. All crosses and assays were performed at room temperature (∼22°C–23°C) and ambient humidity.

We estimated the effect of the mutations on salt sensitivity by applying a Cox proportional hazards model on the fly survival data. The outcome was survival time, and at the end of the follow-up period, all living flies were censored. The data for each genetic background (heterogenic or isogenic) and NaCl concentration were analyzed separately. We estimated the effect on the hazard ratio of genotype (each mutation versus control as baseline). To account for intra-vial correlation, we used robust sandwich variance estimators in a generalized estimating equation (GEE)-like model that treats members of each vial as associated with a single cluster. Analyses were performed with the R “survival” package.

### Mouse Renal Expression Study

129S6 mice were purchased from Taconic Biosciences and were maintained on a 12 hr light-dark cycle with free access to standard chow and water in the animal facility of the University of Virginia. Only male mice at 12 weeks of age were used. High-salt diet (HSD, 6% NaCl) in pellets was purchased from Harlan Teklad and administered in place of normal chow for two weeks. Experiments were carried out in accordance with local and NIH guidelines. To induce CKD, mice were subjected to sub-total nephrectomy (Nx) under 1.5% isoflurane anesthesia, the right kidney was removed, and the upper branch of the two main branches of the left renal artery were ligated to impede blood supply to the upper half of the kidney as previously reported.^31^

Renal mRNA was extracted at the end of 2 weeks of HSD, or at 12 weeks after sub-total Nx. Real-time RT-PCR was performed as previously described^32^ with the primers listed in Table S3. Fluorescence detection was accomplished with Sybr Green and the iCYcler system (Bio-Rad). mRNA expression was normalized against mRNA expression of the *Hprt* housekeeping gene, and the mean at baseline was used as the reference for determination of relative expression across conditions.

## Results

### Identification of Loci Associated with Kidney Function across Ancestry Groups

We identified 20 loci attaining genome-wide-significant evidence of association with eGFR (p < 5 × 10^−8^) in trans-ethnic meta-analysis (Table 1, Figure S2). These loci have been previously reported in ethnic-specific GWASs of individuals with European and East Asian ancestry^4^, ^5^, ^6^, ^8^ (Table S4). They include two loci discovered in a recently published meta-analysis of European ancestry GWASs: *LRP2* ([MIM: 600073] rs57989581, p = 5.6 × 10^−10^) and *NFATC1* ([MIM: 600489] rs8096658, p = 1.3 × 10^−8^). Previously reported lead SNPs at an additional 21 established kidney-function loci attained nominal evidence of association (p < 0.05) with eGFR, with consistent direction of effect (Table S4).

As expected, lead SNPs were common across ancestry groups at all 20 loci, with each displaying modest effects on eGFR (Table S5). Despite substantial variability in allele frequencies between ancestry groups, we observed no evidence of trans-ethnic heterogeneity in allelic effects on eGFR for any lead SNP (Table 1, Table S5). Through conditional analyses (Table S6), we observed no evidence of multiple distinct signals of association for eGFR at any locus (p_COND_ < 10^−5^, Bonferroni correction for ∼5,000 variants per locus). Taken together, these data are consistent with a single variant driving association signals in each locus; each variant is shared across ancestry groups and has homogeneous effects on eGFR in diverse populations. However, we recognize that larger multi-ethnic samples will be required to detect lower frequency, population-specific distinct association signals of modest effect on kidney function.

### Impact of Lead eGFR SNPs on CKD and Kidney Function in Diabetic Individuals

We assessed the impact on CKD of lead SNPs at the 20 eGFR loci in a subset of individuals (up to 3,976 cases and 55,904 controls) contributing to our trans-ethnic meta-analysis (Table S7). We defined CKD by eGFR < 60 mL/min/1.73 m^2^ and/or incidence of ESRD. For all 20 lead SNPs, the eGFR-decreasing allele was associated with increased risk of CKD. Eleven of the lead SNPs demonstrated evidence of association with CKD at nominal significance (p < 0.05), and the strongest signals were observed at *UNCX* (rs62435145, p = 2.2 × 10^−7^), *ALMS1* ([MIM: 606844] rs7587577, p = 3.1 × 10^−6^), and *PDILT-UMOD* ([MIM: 191845] rs77924615, p = 4.0 × 10^−6^).

We also investigated the impact of the lead SNPs on eGFR in GWASs of individuals with diabetes for whom there are different mechanisms for loss of renal function, such as diabetic nephropathy. We obtained association summary statistics for eGFR in 13,158 subjects of European ancestry with diabetes (9,197 with T2D and 3,961 with T1D) from the SUMMIT Consortium (Table S8). Consistent with previous reports,^8^, ^33^ allelic effects on eGFR in diabetic individuals and our trans-ethnic meta-analysis of individuals from the general population were homogeneous (Figure S3). There was nominal evidence of association with eGFR (p < 0.05), with the same direction of effect, at seven loci, and the strongest signals were observed at *PDILT-UMOD* (p = 6.9 × 10^−6^), *PRKAG2* ([MIM: 602743] p = 0.00013) and *NFATC1* (p = 0.00045).

### Fine Mapping of eGFR Loci

We next sought to localize variants driving eGFR association signals in each of the 20 loci attaining genome-wide significance in our trans-ethnic meta-analysis. We utilized trans-ethnic fine mapping implemented in MANTRA,^22^ taking advantage of increased sample size and the expectation that patterns of LD vary between diverse populations. We derived credible sets of variants^25^ mapping within 500 kb of the lead SNP at each locus that together account for 99% of the posterior probability (π_C_) of driving the association signal (Table S9). Smaller credible sets, in terms of the number of SNPs they contain, or the genomic interval that they cover, thus correspond to more precise fine-mapping. The 99% credible set at the *PDILT-UMOD* locus included a single variant (rs77924615, π_C_>0.999), which maps to an intron of *PDILT*. This variant has previously been reported as driving the primary association signal for CKD at the *PDILT-UMOD* locus through whole-genome sequencing and long-range haplotype imputation into 194,286 Icelandic individuals with serum creatinine measurements.^34^ We also observed precise localization, defined by a 99% credible set including no more than five variants (Table S10), at a five additional loci: *NFATC1* (two variants, mapping to 0.4 kb), *SLC34A1* ([MIM: 182309] two variants, mapping to 0.6 kb), *GCKR* ([MIM: 600842] three variants, mapping to 11.7 kb), *DCDC5-MPPED2* ([MIM: 612321, 600911] four variants, mapping to 27.9 kb), and *PIP5K1B* ([MIM: 602745] five variants, mapping to 3.5 kb).

### Integration of Genetic Fine-mapping and Genomic Annotation

To gain insight into the mechanisms through which association signals at the 20 GWAS loci attaining genome-wide significance in our trans-ethnic meta-analysis impact eGFR, we began by obtaining genomic annotations for all single-nucleotide variants mapping within 500 kb of lead SNPs. Across all 20 loci, only 5.4% of the posterior probability of driving association signals was annotated to coding variants (Table S11), the majority of which was accounted for by *GCKR* p.Pro446Leu (rs1260326, π_C_ = 0.938). This missense variant has been shown, functionally, to result in increased de novo triglyceride and cholesterol synthesis and export and decreased plasma glucose concentrations, all of which have been associated with risk of CKD,^35^, ^36^ making *GCKR* the likely effector transcript for eGFR at this locus. However, outside of the *GCKR* locus, variants mapping to non-coding sequence accounted for more than 99.4% of the probability of driving eGFR association, suggesting that these signals are most likely to be mediated by effects on gene regulation.

**Figure 1:**
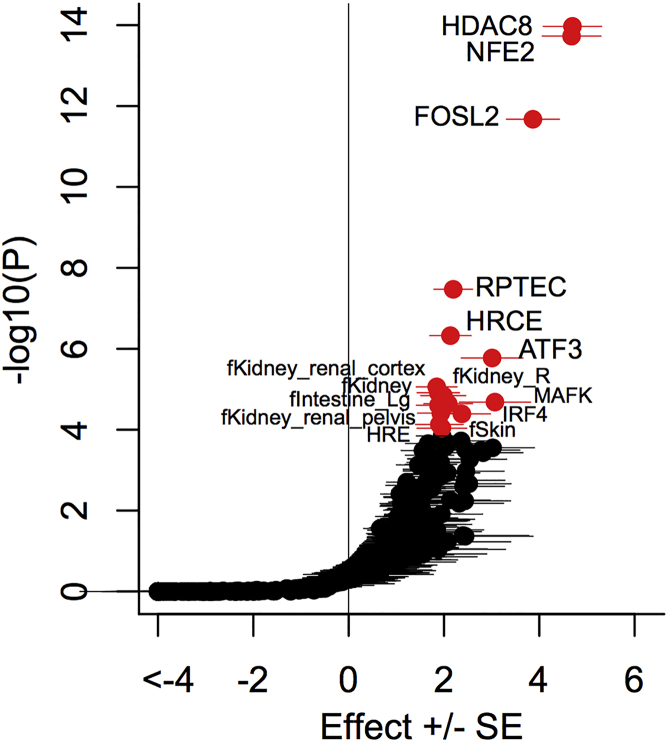
DNase I Hypersensitivity Sites in Kidney Cells and HDAC8 Binding Sites are Predictive of Posterior Probability of Driving Association Signals at 20 eGFR Loci We tested whether genomic annotations of regulatory chromatin state for 93 cell types, DNase I hypersensitivity sites (DHSs) for 145 cell types, and chromatin immuno-precipitation sequence binding sites for 165 transcription factors were predictive of posterior probability of driving eGFR association signals. Each point corresponds to an annotation, plotted according to the effect size (log-odds ratio for driving association signal) on the x axis and ranked according to the significance of the association on the y axis. Significant association (p < 0.00012, highlighted in red) was defined by Bonferroni correction for 403 tested annotations. The most significant effects included DHSs in kidney cells (RPTECs and HRCEs) and binding sites for HDAC8.

We next investigated whether genomic annotations of regulatory chromatin state for 93 cell types,^26^ DHSs for 145 cell types,^27^ and ChIP-seq binding sites for 165 transcription factors^27^, ^28^ were predictive of posterior probability of driving association signals across the 20 loci (Figure 1, Table S12). We observed significant effects (p < 0.00012, Bonferroni correction for 403 annotations) on posterior probability for variants in kidney DHSs, including adult renal proximal tubular epithelial cells (RPTECs; p = 3.4 × 10^−8^), renal cortical epithelial cells (HRCEs; p = 4.7 ×10^−7^), and fetal kidney cells (p = 8.8 × 10^−6^). We also observed significant effects on posterior probability for transcription-factor binding sites, most notably for HDAC8 (p = 1.1 × 10^−14^). Histone deacetylases (HDACs) are involved in kidney function and development,^37^ and HDAC inhibitors could be promising in the treatment of kidney disease.^38^ We repeated our analyses by using fGWAS^29^ (Figure S4, Table S12) and observed strong correlation in the ranking of enriched annotations (*r*^2^ = 0.93). These results highlight that variants driving association signals with eGFR are more likely to be co-localized with annotated elements in kidney cells, thereby suggesting that gene regulation in disease-relevant tissues is a likely mechanism by which GWAS loci impact CKD.

Lead SNPs that, by themselves, accounted for more than 80% of the posterior probability of driving association signals overlapped an enriched annotation at five loci (Table S13). In particular, at the *SLC34A1* locus, rs35716097 (π_C_ = 0.946) overlapped DHSs in RPTECs and HRCEs, as well as a binding site for HDAC8, while at the *NFATC1* locus, rs8096658 (π_C_ = 0.877) overlapped fetal kidney cell DHSs (Figure S5). At both of these loci, the lead SNPs were also expression quantitative trait loci (eQTLs) for *NFATC1* and *RGS14* (MIM: 602513; at the *SLC34A1* locus) in multiple tissues (Table S13), highlighting these genes as likely effector transcripts through which eGFR association signals are mediated. *NFATC1* plays a central role in inducible gene transcription during immune response and is a downstream target of the transplant immunosuppression drug cyclosporine A. *RGS14* encodes a member of the regulator of G protein signaling family, which modulates downstream effects of Gα subunits and has unknown function in kidneys.

### Experimental Data in Model Organisms

**Figure 2:**
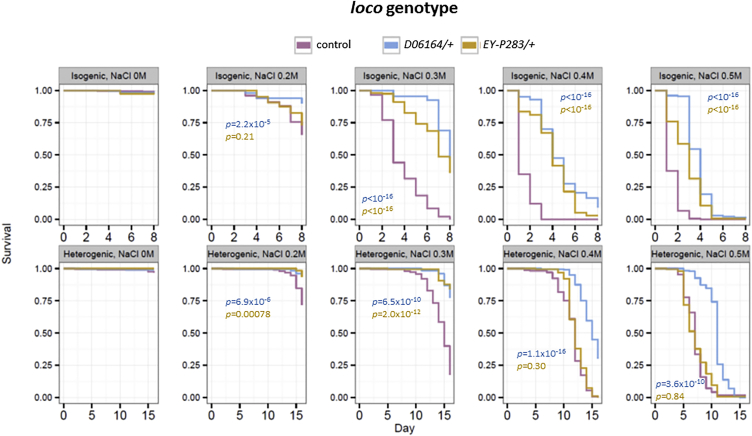
*Drosophila RGS14* Heterozygous Mutants Are Resistant to Salt Stress Survival of flies carrying heterozygous loss-of-function mutations in the *Drosophila melanogaster RGS14* homolog, *loco*, was compared to that of controls of the same genetic background. In the isogenic experiment, all genotypes were backcrossed to the control strain. In the heterogenic experiment, controls and *loco* mutants were crossed with the A.R.R. lab’s *wBerlin* strain to obtain highly heterozygous progeny. Kaplan-Meier plots demonstrated that flies heterozygous for two independently derived loco mutations, *loco*^*EY-P283*^ and *loco*^*d06164*^, were resistant to salt stress across a range of NaCl concentrations when compared to controls. Cox-proportional hazards p values for each mutant, compared to those of controls, are presented and are calculated for each genetic background (isogenic or heterogenic) and NaCl concentration separately. Results are based on 170–200 flies per genotype for each NaCl concentration.

To provide insight into the role of *NFATC1* and *RGS14* (at the *SLC34A1* locus) in kidney physiology, we examined the function of ancestral orthologs in *Drosophila melanogaster*. The *Drosophila* genome encodes a single member of the *NFAT* family, and a previous report has demonstrated that flies with *NFAT* loss-of-function mutations have increased salt sensitivity, suggesting a role for this gene in ionic or osmotic regulation.^39^ The closest *RGS14* ortholog in *Drosophila melanogaster* is *loco*, for which reduced expression is associated with longer lifespan and stress resistance.^30^ We thus conducted experiments aimed at characterizing a role for *loco* loss-of-function variants in salt sensitivity. We compared survival of two independently derived heterozygous *loco* mutants (*y*^*1*^*w*^*1*^*; loco*^*d06164*^*/+* and *y*^*1*^*w*^*1*^*; loco*^*EY-P283*^*/+*) with isogenic *y*^*1*^*w*^*1*^ controls after supplementing their diet with varying NaCl concentrations for 8 days (Figure 2). There was very little mortality of any of the genotypes on non-NaCl-supplemented food, indicating no baseline differences in viability over the time period tested. However, we observed significantly improved survival of the heterozygous *loco* mutants over controls on NaCl-supplemented food (Figure 2, Table S14), thereby indicating a role for this gene in resistance to salt stress. To exclude the effects of inbreeding depression on our findings, we also repeated our experiments with the same strains on a heterogenic background. As expected, the hybrid heterogenic strains were less salt susceptible than the isogenic strains, but the *loco* mutants remained salt-resistant when compared to controls of a similar genetic background (Figure 2, Table S14).

**Figure 3:**
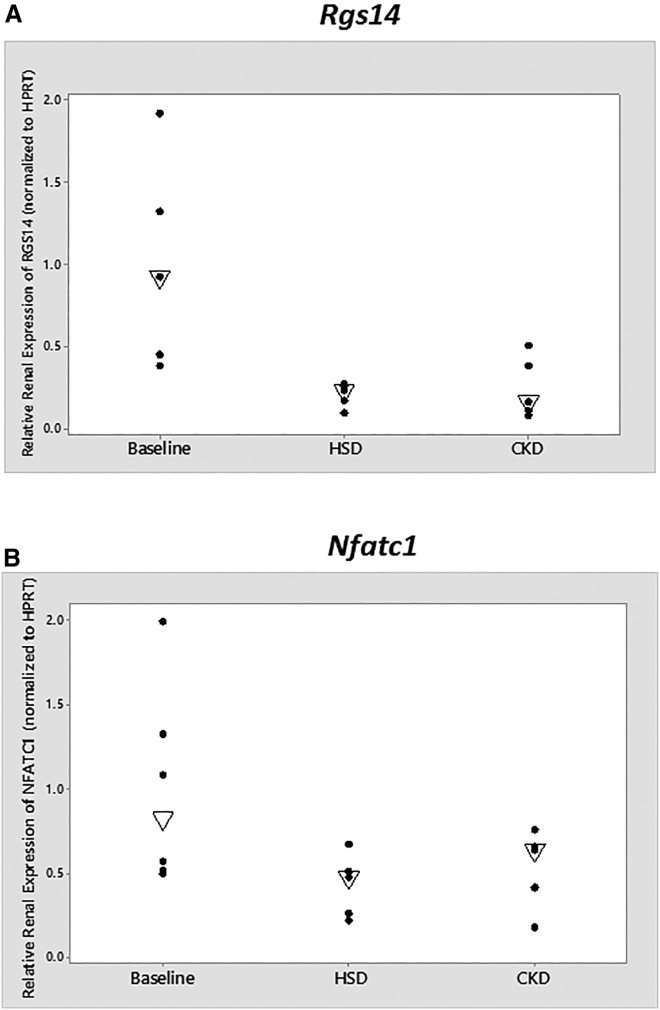
Relative Renal mRNA Expression of *Rgs14* and *Nfatc1* Expression of *Rgs14* is shown in (A) and *Nfatc1* in (B). n = 5 or 6 in each group. The empty triangle represents the median. According to the Mann-Whitney test, and compared to the baseline, *Rgs14* expression after exposure to a high-salt diet was significantly lower (p = 0.01), and was also lower in CKD (p = 0.06). *Nfatc1* expression after exposure to a high-salt diet was also significantly lower (p = 0.03) and trended in the same direction in CKD (p = 0.31).

To further investigate the role of *NFATC1* and *RGS14* in kidney function, we used the 129S6 mouse strain that is salt-sensitive^31^ and susceptible to glomerulosclerosis.^40^ We compared the renal mRNA expression of *Nfatc1* and *Rgs14* at baseline versus (1) after a 2-week exposure to high-salt diet and (2) at 12 weeks after CKD induced by sub-total nephrectomy. Compared to baseline condition, *Rgs14* was significantly decreased (∼75%, p = 0.01) during high-salt exposure (Figure 3). In the CKD model, *Rgs14* expression was also reduced and approached statistical significance (p = 0.06). The renal mRNA expression of *Nfatc1* was also significantly decreased (∼50%, p = 0.03) during high-salt exposure and trended down in CKD (p = 0.31). Although we cannot establish cause and effect, these data illustrate that the expression of both genes is altered during disease states.

## Discussion

We have undertaken a trans-ethnic meta-analysis of GWASs of eGFR, supplemented by imputation up to the phase 1 integrated (March 2012 release) multi-ethnic reference panel from the 1000 Genomes Project.^9^ With these high-density imputed data, we identified 20 loci at genome-wide significance for eGFR through trans-ethnic meta-analysis. Despite improved coverage of low-frequency variation offered by high-density imputation, lead SNPs were common across ancestry groups at all 20 of these kidney-function loci. There was also minimal evidence of trans-ethnic heterogeneity in allelic effects on eGFR at lead SNPs at kidney-function loci, thereby arguing against the “synthetic association” hypothesis.^41^ It is highly unlikely that eGFR association signals at these kidney-function loci reflect unobserved lower frequency causal alleles with larger effects because (1) rare variants are unlikely to have arisen before human population migration out of Africa and thus are not anticipated to be widely shared across diverse populations^9^, ^42^ and (2) LD with these variants is expected to be highly variable between ethnicities.

Our conditional analyses did not provide evidence for multiple distinct eGFR association signals, which is consistent with a single causal variant at each of the 20 eGFR loci. However, we recognize that conditional analyses evaluate the evidence for residual association at the locus that cannot be ascribed to the lead SNP and do not provide a formal framework to test for the presence of multiple causal variants, for example, that are in strong LD with each other and reside on the same haplotype. Furthermore, larger sample sizes will be required to detect distinct association signals defined by common variants of modest effect or low-frequency variants that might be specific to particular ethnic groups.

As with most previous GWASs of kidney function, our study was limited to a single measure of eGFR for each participant. We also did not adjust for diabetes or hypertension in our analyses given that these conditions are potential mediators or modifiers of the SNP-eGFR associations. However, despite ethnic differences in the prevalence of these conditions, we observed no evidence of heterogeneity in allelic effects on eGFR between ancestry groups. Exploration of context-dependent effects should be considered in future studies, for example, by using gene-environment interaction or mediation analyses.

Given our observation that eGFR association signals are shared across ancestry groups, we next sought to take advantage of the differential patterns of LD across diverse populations to fine-map kidney-function loci. Credible-set variants mapped predominantly to non-coding sequence, suggesting that eGFR association signals are most likely to be mediated by effects on gene regulation, in agreement with previous reports for other complex human traits.^43^, ^44^, ^45^ Through integration of genetic fine-mapping data with information from regulatory annotation resources, we have demonstrated significant enrichment of variants driving eGFR association signals with DHSs in multiple kidney cell types. Overlap with these enriched annotations could be used as a prior model for eGFR association signals, genome-wide, to improve power for discovery of additional kidney-function loci and further enhance trans-ethnic fine-mapping efforts.^46^

Lead SNPs at kidney-function loci overlapping enriched annotations included eQTL for *NFATC1* and *RGS14* (at the *SLC34A1* locus) in multiple tissues, pointing to likely effector transcripts through which these eGFR association signals are mediated. We have established that loss-of-function mutations in ancestral orthologs of both genes in *Drosophila melanogaster* are associated with response to salt stress. Although salt sensitivity has not been directly correlated with variation in eGFR in humans, it has been associated with albuminuria,^47^, ^48^ elevated creatinine,^48^ and the subsequent development of hypertension,^49^ suggesting the relevance of this trait to kidney function. Indeed, in animal models, salt sensitivity is tightly linked with a blunted tubuloglomerular feedback (TGF) or impaired increase in GFR after salt loading.^50^, ^51^, ^52^, ^53^ Consistent with this, we demonstrated that renal mRNA expression of *Nfatc1* and *Rgs14* in a salt-sensitive mouse model was reduced after exposure to a high-salt diet and induced CKD. In parallel with the findings in *Drosophila melanogaster*, these results are consistent with the hypothesis that the capacity to reduce expression of *Rgs14* and *Nfatc1* determines the extent of the response to stress. Another possible mechanism suggested by our results in *Drosophila* is a role for oxidative stress, to which *RGS14* ortholog mutants are resistant,^30^ and which has been implicated in mammalian salt sensitivity.^54^, ^55^ Establishing the functional role of these genes in salt sensitivity, TGF, GFR, oxidative stress, and CKD will require targeted in vivo studies using knockout and/or transgenic mouse models.

In conclusion, our study demonstrates the utility of trans-ethnic fine mapping through integration of GWASs of diverse populations with genomic annotation from relevant tissues to define molecular mechanisms by which association signals exert their effect, thereby offering an exciting opportunity to elucidate the pathophysiology of complex human diseases.

## Figures and Tables

**Table 1:** Loci Attaining Genome-wide-Significant Evidence of Association (p < 5 × 10^−8^) with eGFR in Trans-ethnic Meta-analysis of 71,638 Individuals

**Locus**	**Lead SNP**	**Chr**	**Position (bp, b37)**	**Alleles**		**Trans-ethnic Meta-analysis**				
**Effect**^a^	**Other**	**Beta**	**SE**	**p Value**	**Cochran’s Q p Value**	**N**
*SLC43A1*	rs35716097	5	176,806,636	T	C	−1.097	0.127	2.3 × 10^−17^	0.13	71,638
*SHROOM3*	rs5020545	4	77,414,988	T	C	−0.969	0.119	1.3 × 10^−15^	0.010	71,638
*PDILT-UMOD*	rs77924615	16	20,392,332	G	A	−1.185	0.147	1.7 × 10^−15^	0.011	71,638
*UNCX*	rs62435145	7	1,286,567	T	G	−1.092	0.137	4.7 × 10^−15^	0.17	59,865
*GCKR*	rs1260326	2	27,730,940	C	T	−0.872	0.114	6.1 × 10^−14^	0.069	71,638
*BCAS3*	rs9895661	17	59,456,589	C	T	−1.003	0.132	7.9 × 10^−14^	0.19	71,638
*SPATA5L1-GATM*	rs2486288	15	45,712,339	C	T	−0.883	0.126	4.7 × 10^−12^	0.76	71,638
*ALMS1*	rs7587577	2	73,832,786	C	T	−0.948	0.135	5.2 × 10^−12^	0.098	48,102
*CPS1*	rs715	2	211,543,055	C	T	−0.876	0.127	1.3 × 10^−11^	0.21	71,638
*WDR72*	rs1031755	15	53,951,435	A	C	−0.860	0.127	2.2 × 10^−11^	0.0013	71,638
*PIP5K1B*	rs4744712	9	71,434,707	A	C	−0.753	0.112	3.3 × 10^−11^	0.91	71,638
*PRKAG2*	rs10265221	7	151,414,329	C	T	−0.963	0.146	7.3 × 10^−11^	0.23	71,638
*DAB2-C9*	chr5: 39,404,526:D	5	39,404,526	D	R	−0.817	0.126	1.5 × 10^−10^	0.80	48,102
*LRP2*	rs57989581	2	170,194,459	C	A	−1.980	0.315	5.6 × 10^−10^	0.16	71,638
*SLC22A2*	rs316009	6	160,675,764	C	T	−1.193	0.192	1.0 × 10^−9^	0.49	71,638
*LOC100132354-VEGFA*	rs881858	6	43,806,609	A	G	−0.772	0.127	2.0 × 10^−9^	0.0020	71,638
*DCDC5-MPPED2*	rs963837	11	30,749,090	T	C	−0.685	0.114	3.7 × 10^−9^	0.0034	71,638
*NFATC1*	rs8096658	18	77,156,537	G	C	−0.814	0.141	1.3 × 10^−8^	0.015	59,865
*PHTF2*	rs848486	7	77,552,127	G	A	−0.643	0.113	2.0 × 10^−8^	0.83	71,638
*TFDP2*	rs1511299	3	141,716,072	T	C	−0.727	0.131	4.4 × 10^−8^	0.55	71,638

a Effect allele is eGFR-decreasing allele.

## Web Resources

1000 Genomes, http://www.1000genomes.org
ENCODE, https://www.encodeproject.org/
Ensembl Genome Browser, http://www.ensembl.org/index.html
EPACTS, http://genome.sph.umich.edu/wiki/EPACTS
fGWAS, https://github.com/joepickrell/fgwas
Gencode, http://www.gencodegenes.org
GWAMA, http://www.geenivaramu.ee/en/tools/gwama
IMPUTE2, http://mathgen.stats.ox.ac.uk/impute/impute_v2.html
International HapMap Project, http://hapmap.ncbi.nlm.nih.gov/
LDlink, http://analysistools.nci.nih.gov/LDlink/
METAL, http://www.sph.umich.edu/csg/abecasis/metal/
Minimac, http://genome.sph.umich.edu/wiki/Minimac
OMIM, http://www.omim.org/
Roadmap, http://www.roadmapepigenomics.org/
Variant Effect Predictor, http://useast.ensembl.org/Homo_sapiens/Tools/VEP

## Supplemental Data

Document S1. Figures S1–S5, Tables S1–S14, and Supplemental Acknowledgments Document S1. Figures S1–S5, Tables S1–S14, and Supplemental Acknowledgments

Document S2. Article plus Supplemental Data Document S2. Article plus Supplemental Data
