# Supplementary material for: Trans-ethnic Fine Mapping Highlights Kidney-Function Genes Linked to Salt Sensitivity
Source: Am J Hum Genet. 2016 Sep 1;99(3):636–46. doi: 10.1016/j.ajhg.2016.07.012 (PMC5011075; doi:10.1016/j.ajhg.2016.07.012)
Supplement: Document S1. Figures S1–S5, Tables S1–S14, and Supplemental Acknowledgments [file mmc1.pdf]

The American Journal of Human Genetics, Volume *99*

Supplemental Data

Trans-ethnic Fine Mapping Highlights Kidney-Function Genes Linked to Salt Sensitivity

Anubha Mahajan, Aylin R. Rodan, Thu H. Le, Kyle J. Gaulton, Jeffrey Haessler, Adrienne M. Stilp, Yoichiro Kamatani, Gu Zhu, Tamar Sofer, Sanjana Puri, Jeffrey N. Schellinger, Pei-Lun Chu, Sylvia Cechova, Natalie van Zuydam, the SUMMIT Consortium, the BioBank Japan Project, Johan Arnlov, Michael F. Flessner, Vilmantas Giedraitis, Andrew C. Heath, Michiaki Kubo, Anders Larsson, Cecilia M. Lindgren, Pamela A.F. Madden, Grant W. Montgomery, George J. Papanicolaou, Alex P. Reiner, Johan Sundström, Timothy A. Thornton, Lars Lind, Erik Ingelsson, Jianwen Cai, Nicholas G. Martin, Charles Kooperberg, Koichi Matsuda, John B. Whitfield, Yukinori Okada, Cathy C. Laurie, Andrew P. Morris, and Nora Franceschini

Figure S1. Dendogram to summarise relatedness between studies. The dendogram was constructed on the basis of genome-wide pair-wise allele frequency differences between studies. European ancestry studies are grouped in the red cluster, and Hispanic ancestry studies are grouped in the blue cluster.

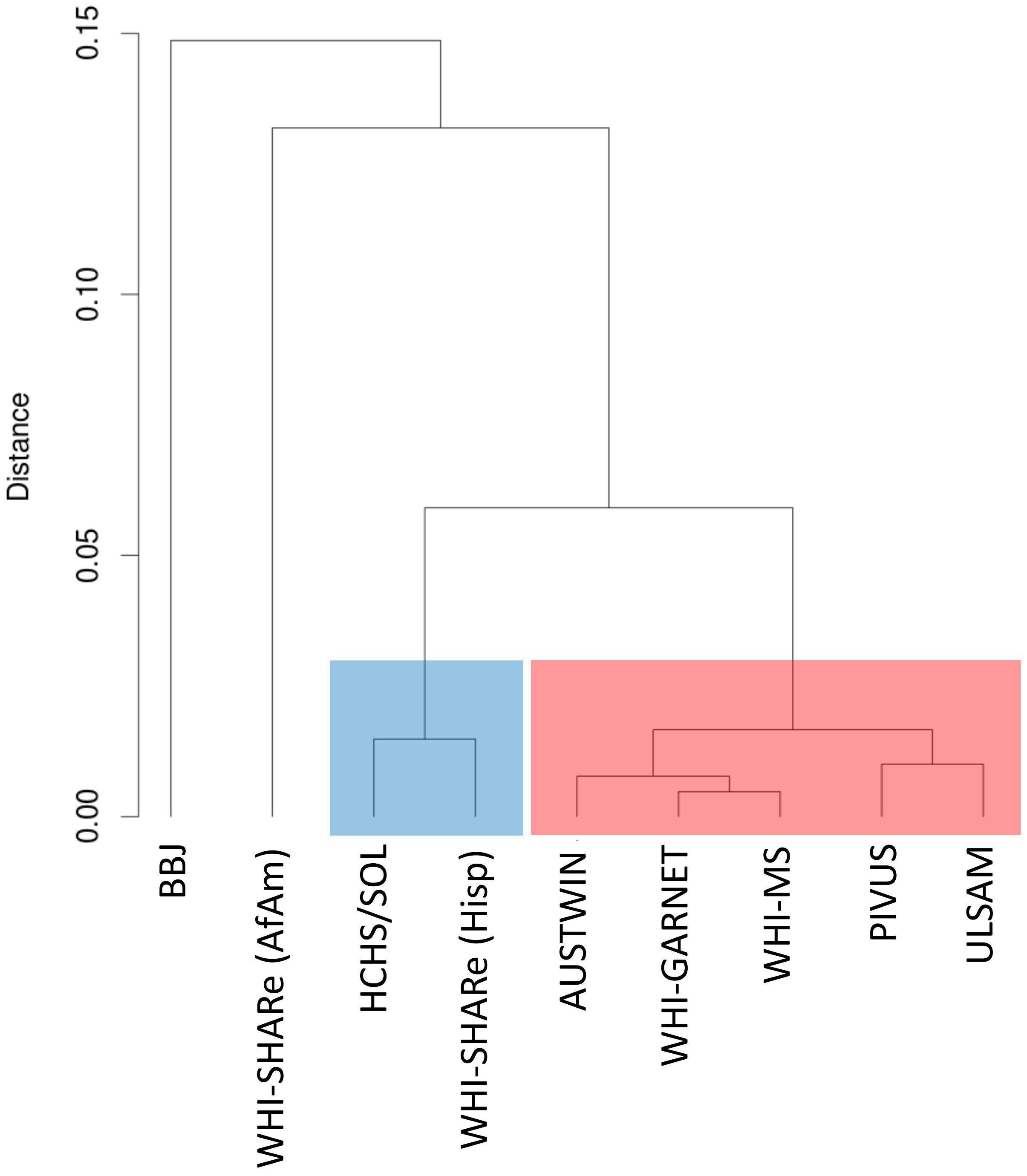

Figure S2. Genome-wide eGFR association summary from the trans-ethnic meta-analysis of 71,638 individuals. Each point corresponds to a SNP passing quality control in the meta-analysis, plotted according to physical position (NCBI build 37) on the x-axis and $-\log_{10}$ *p*-value on the y-axis. The locus names of loci attaining genome-wide significance ($p<5\times10^{-8}$, horizontal red line) are highlighted above the Manhattan plot. Association signals mapping to previously established loci are highlighted in green.

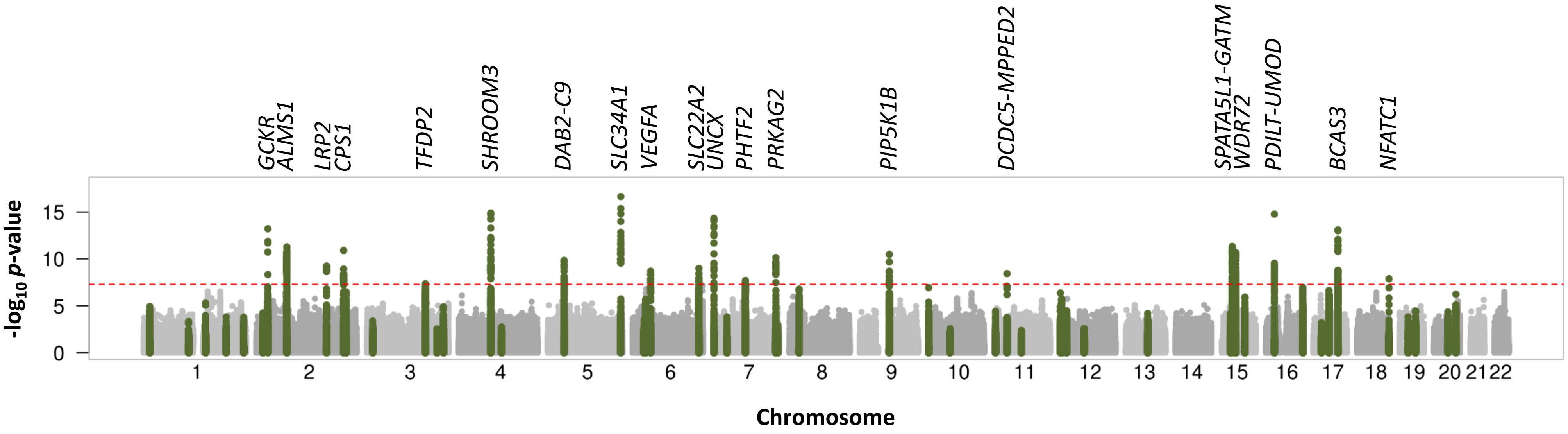

Figure S3. Comparison of allelic effects (beta) of lead SNPs on eGFR in the general population (from our trans-ethnic meta-analysis of 71,638 individuals) and in diabetics (from a meta-analysis of 13,158 individuals from the SUMMIT Consortium). Grey bars represent 95% confidence intervals for allelic effect sizes. The lead SNP at the *PDILT-UMOD* locus demonstrates greater allelic effect on eGFR in diabetics than in the general population at nominal significance ($p<0.05$).

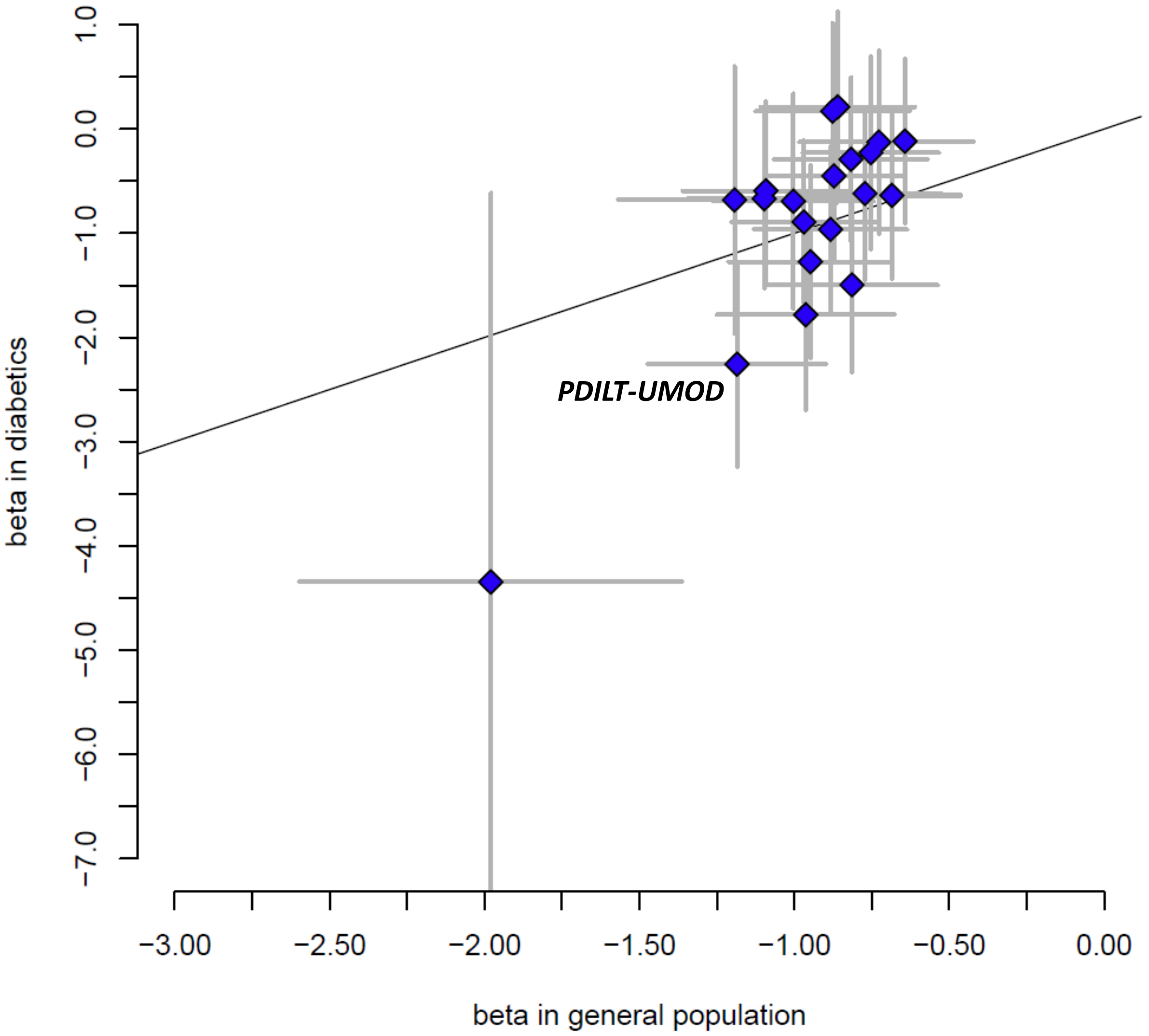

Figure S4. Enrichment of genomic annotations of regulatory chromatin state for 93 cell types, DHS for 145 cell types, and chromatin immuno-precipitation sequence binding sites for 165 transcription factors for Bayes' factors in favour of eGFR association. Each point corresponds to an annotation, plotted according to the effect size (log-enrichment in Bayes' factor) on the x-axis, and ranked according to the significance of the association on the y-axis. Significant enrichments are highlighted in red.

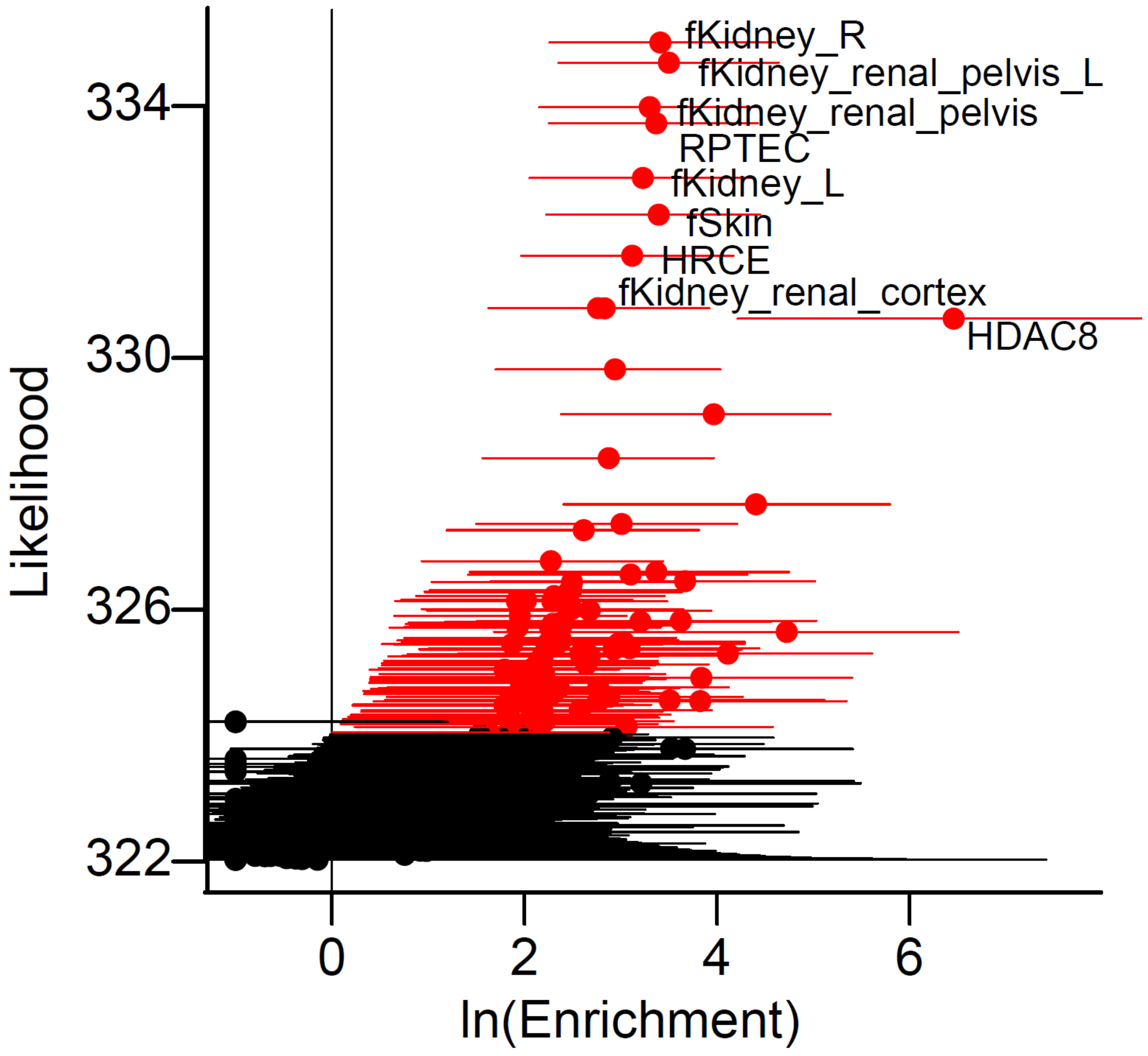

Figure S5. Overlap of credible set variants with enriched regulatory annotations at the *SLC34A1* and *NFATC1* loci. Each point corresponds to a SNP, plotted according to their chromosomal position and posterior probability of driving the eGFR association signal. The locations of enriched regulatory annotations (DHS in multiple kidney cell-types and TFBS for HDAC8) are highlighted for each locus.

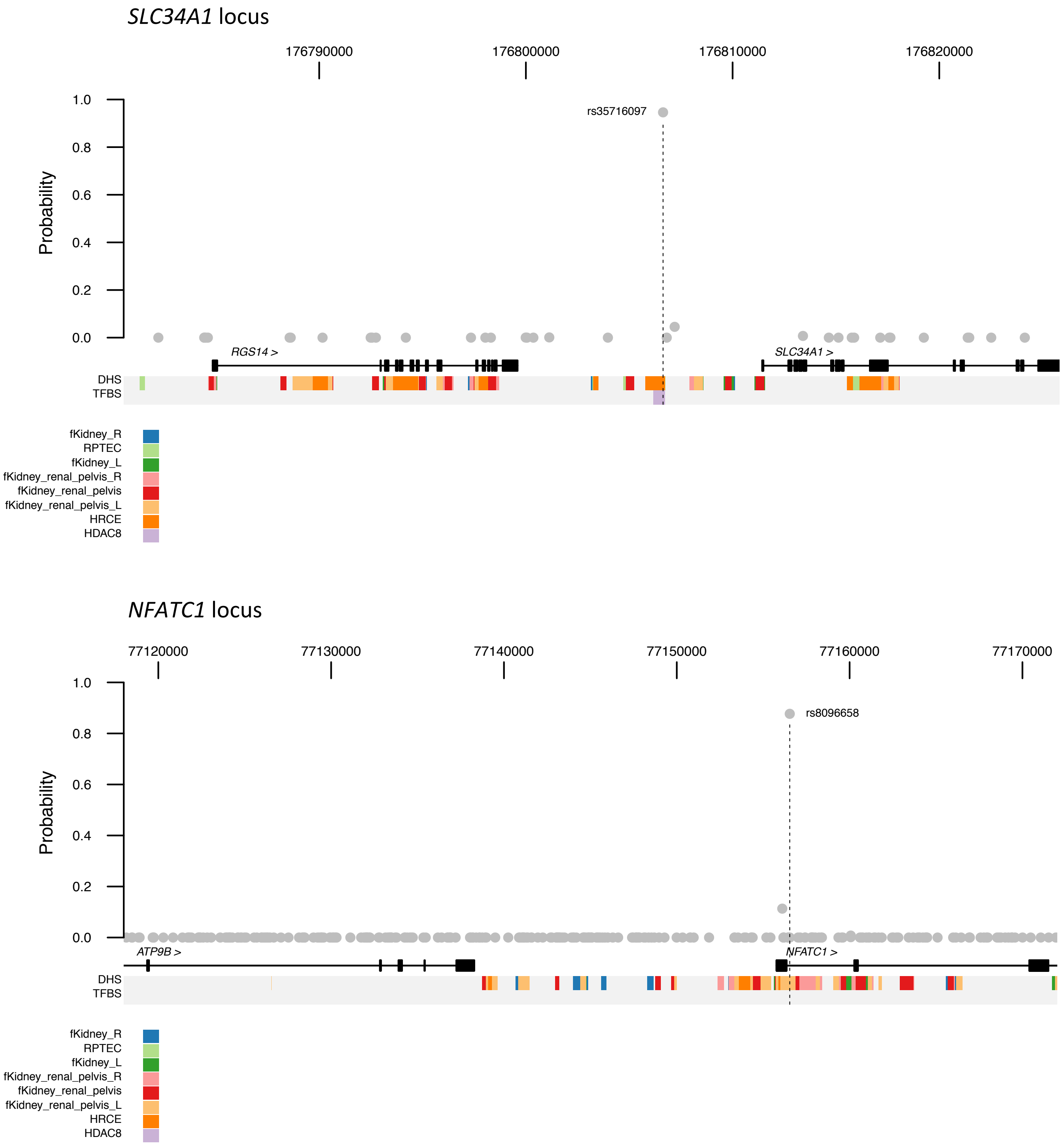

Table S1. Study sample characteristics.

Study (acronym)	Ethnicity (origin)	Sex	Sample characteristics				
			Sample size	Age (years) mean (SD)	Serum Creatinine (mg/dL) mean (SD)	eGFR mean (SD)	CKD cases/controls
Prospective Investigation of the Vasculature in Uppsala Seniors (PIVUS)	European (Sweden)	Males	471	70.1 (0.1)	0.99 (0.22)	83.8 (19.9)	136/808
		Females	473	70.2 (0.2)	0.82 (0.18)	77.9 (20.2)	
Uppsala Longitudinal Study of Adult Men (ULSAM)	European (Sweden)	Males	1,080	71.0 (0.6)	1.06 (0.15)	75.2 (11.3)	88/992
		Females	0	N/A	N/A	N/A	
Australian Twin-Family Studies (AUSTWIN)	European (Australia)	Males	4,662	48.7 (13.1)	1.13 (0.20)	76.6 (15.8)	NA/NA
		Females	7,096	46.9 (13.4)	0.90 (0.16)	75.1 (16.5)	
Women's Health Initiative Memory Study (WHI-MS)	European (USA)	Males	0	N/A	N/A	N/A	343/5,312
		Females	5,655	68.1 (5.9)	0.75 (0.15)	85.6 (17.8)	
Women's Health Initiative Genome-wide Association Research Network into Effects of Treatment (WHI-GARNET)	European (USA)	Males	0	N/A	N/A	N/A	240/3,876
		Females	4,116	65.6 (6.9)	0.74 (0.15)	88.1 (19.3)	
BioBank Japan Project (BBJ)	East Asian (Japan)	Males	12,802	64.4 (9.8)	0.89 (0.29)	100.2 (28.5)	1,330/22,206
		Females	10,734	60.7 (13.1)	0.64 (0.20)	109.1 (31.0)	
Hispanic Community Health Study and Study of Latinos (HCHS/SOL)	Hispanic (USA)	Males	5,242	45.3 (14.2)	0.98 (0.44)	95.5 (22.3)	462/12,314
		Females	7,534	46.7 (13.6)	0.73 (0.23)	96.6 (23.4)	
Women's Health Initiative SNP Health Association Resource (WHI-SHARe)	Hispanic (USA)	Males	0	N/A	N/A	N/A	174/3,375
		Females	3,549	60.3 (6.7)	0.71 (0.19)	94.7 (21.9)	
	African American (USA)	Males	0	N/A	N/A	N/A	1,203/7,021
		Females	8,224	61.6 (7.0)	0.82 (0.22)	80.1 (19.4)	

SD: standard deviation.

Table S2. Summary of study-specific genotyping, quality control, imputation and analysis.

Study acronym	Genotyping array	Sample quality control		Scaffold quality control			Pre-phasing and imputation			Association analysis		
		**Call rate**	**Exclusions**	**Call rate**	**HWE *p***	**MAF**	**Software**	**Quality filter**	**Passed SNPs**	**Software**	**Covariates**	λ_{GC}
PIVUS	Illumina OmniExpress & Metabochip	95%	Heterozygosity, gender check and relatedness	95% (99% if MAF<5%)	10^{-6}	1%	SHAPEITv2 IMPUTEv2	info≥0.4	9,316,737	SNPTESTv2	Age, sex, 2 PCs	0.982
ULSAM	Illumina Omni2.5M & Metabochip	95%	Heterozygosity, gender check and relatedness	95% (99% if MAF<5%)	10^{-6}	1%	SHAPEITv2 IMPUTEv2	info≥0.4	9,388,420	SNPTESTv2	Age, 2 PCs	1.013
AUSTWIN	Illumina 317K, 370K, 610K, OmniExpress, Omni2.5 & HumanCoreExome	95%	Heterozygosity, gender check and relatedness	95%	10^{-6}	1%	MaCH minimac	r^2≥0.3	8,584,822	MERLIN	Age, sex, sub-study, 10 PCs	1.120
WHI-MS	Illumina OmniExpress-Exome	None	Ethnic outliers, gender check, relatedness and duplicates	97%	10^{-4}	1%	Beagle minimac	r^2≥0.3	8,814,333	ProbAbel/R	Age, centre, 10 PCs	1.025
WHI-GARNET	Illumina Human Omni1-Quad	None	Ethnic outliers, gender check, relatedness and duplicates	98%	10^{-4}	None	Beagle minimac	r^2≥0.3	8,864,693	ProbAbel/R	Age, centre, 10 PCs	1.018
BBJ	Iluumina HumanHap 610-Quad	98%	Ethnic outliers and relatedness	99%	10^{-7}	1%	MaCH minimac	r^2≥0.5	6,581,000	mach2qtl	None	1.058
HCHS/SOL	Illumina Omni2.5M & custom	98%	Gender check and duplicates	98%	10^{-5}	None	SHAPEITv2 IMPUTEv2	info≥0.4	11,374,299	LMM-OPS[a]	Age, sex, centre, sampling weights, 5PCs	1.006
WHI-SHARe (Hispanic)	Affymetrix 6.0	95%	Ethnic outliers, gender check, relatedness and duplicates	95%	10^{-6}	1%	MaCH	r^2≥0.3	10,025,812	ProbAbel	Age, centre, 10 PCs	1.027
WHI-SHARe (African American)	Affymetrix 6.0	95%	Ethnic outliers, gender check, relatedness and duplicates	95%	10^{-6}	1%	MaCH	r^2≥0.3	15,345,552	ProbAbel	Age, centre, 10 PCs	1.033

HWE: Hardy-Weinberg equilibrium. MAF: minor allele frequency. PC: principal component.
[a]In-house software, not yet publicly available; accounts for relatedness in linear mixed model.

Table S3. Real-time RT-PCR, oligonucleotide primers.

Gene	Primer Sequence
Rgs14	Forward: 5’-TGAGCCCAGTGAACATCGAC -3’<br>Reverse: 5’- TGTGCTCGGAACATATCTGGC-3'
Nfatc1	Forward: 5’-TGCCTTTTGCGAGCAGTATCT-3’<br>Reverse: 5’-CAGGCAAGGATGGGCTCATAT-3’

Table S4. Association summary statistics for eGFR at previously reported lead SNPs in established loci in trans-ethnic meta-analysis of 71,638 individuals.

Locus	SNP	Chr	Position (bp, b37)	Alleles		Association summary statistics				Reference
				Effect[a]	Other	Beta	SE	*p*-value	*N*	
CASP9	rs12124078	1	15,869,899	G	A	-0.437	0.115	0.00019	71,636	Pattaro *et al*. (2012)[5]
SYPL2	rs12136063	1	110,014,170	G	A	-0.172	0.148	0.25	61,867	Pattaro *et al*. (2016)[8]
LASS2	rs267734	1	150,951,477	T	C	-0.311	0.158	0.052	71,638	Kottgen *et al*. (2010)[4]
CACNA1S	rs3850625	1	201,016,296	G	A	-0.795	0.207	0.00016	71,638	Pattaro *et al*. (2016)[8]
SDCCAG8	rs2802729	1	243,501,763	A	C	-0.323	0.118	0.0068	71,638	Pattaro *et al*. (2016)[8]
DDX1	rs6431731	2	15,863,002	T	C	-0.456	0.322	0.16	48,102	Pattaro *et al*. (2012)[5]
GCKR	rs1260326	2	27,730,940	C	T	-0.872	0.114	6.1×10^{-14}	71,638	Kottgen *et al*. (2010)[4]
ALMS1	rs13538	2	73,868,328	A	G	-0.920	0.140	9.2×10^{-11}	48,102	Kottgen *et al*. (2010)[4]
LRP2	rs4667594	2	170,008,506	A	T	-0.263	0.115	0.025	71,637	Pattaro *et al*. (2016)[8]
CPS1	rs7422339	2	211,540,507	A	C	-0.771	0.125	1.2×10^{-9}	71,638	Kottgen *et al*. (2010)[4]
IGFBP5	rs2712184	2	217,682,779	A	C	-0.573	0.114	7.6×10^{-7}	65,983	Pattaro *et al*. (2016)[8]
WNT7A	rs6795744	3	13,906,850	G	A	-0.159	0.156	0.32	71,638	Pattaro *et al*. (2016)[8]
TFDP2	rs347685	3	141,807,137	A	C	-0.637	0.123	3.0×10^{-7}	71,638	Kottgen *et al*. (2010)[4]
SKIL	rs9682041	3	170,091,902	T	C	-0.141	0.159	0.38	71,638	Pattaro *et al*. (2016)[8]
ETV5	rs10513801	3	185,822,353	G	T	-0.341	0.194	0.083	71,638	Pattaro *et al*. (2016)[8]
SHROOM3	rs17319721	4	77,368,847	A	G	-0.815	0.120	2.2×10^{-11}	71,638	Kottgen *et al*. (2010)[4]
NFKB1	rs228611	4	103,561,709	A	G	-0.351	0.124	0.0052	48,101	Pattaro *et al*. (2016)[8]
DAB2-C9	rs11959928	5	39,397,132	A	T	-0.719	0.113	4.1×10^{-10}	71,638	Kottgen *et al*. (2010)[4]
SLC34A1	rs6420094	5	176,817,636	G	A	-0.804	0.123	1.1×10^{-10}	71,638	Kottgen *et al*. (2010)[4]
ZNF204	rs7759001	6	27,341,409	A	G	-0.233	0.138	0.099	61,867	Pattaro *et al*. (2016)[8]
MHC region	rs3828890	6	31,440,669	C	G	-0.089	0.194	0.65	59,865	Okada *et al*. (2012)[6]
LOC100132354-VEGFA	rs881858	6	43,806,609	A	G	-0.772	0.127	2.0×10^{-9}	71,638	Kottgen *et al*. (2010)[4]
SLC22A2	rs2279463	6	160,668,389	G	A	-0.905	0.169	1.4×10^{-7}	71,638	Kottgen *et al*. (2010)[4]
UNCX	rs10277115	7	1,285,195	T	A	-1.089	0.141	3.3×10^{-14}	59,865	Okada *et al*. (2012)[6]
KBTBD2	rs3750082	7	32,919,927	T	A	-0.441	0.118	0.00025	71,638	Pattaro *et al*. (2016)[8]
PHTF2	rs6465825	7	77,416,439	C	T	-0.590	0.125	3.4×10^{-6}	61,867	Kottgen *et al*. (2010)[4]
PRKAG2	rs7805747	7	151,407,801	A	G	-0.813	0.136	4.4×10^{-9}	48,102	Kottgen *et al*. (2010)[4]
RNF32	rs6459680	7	156,258,568	T	G	-0.315	0.120	0.0097	71,638	Pattaro *et al*. (2016)[8]
STC1	rs10109414	8	23,751,151	T	C	-0.605	0.116	2.5×10^{-7}	71,637	Kottgen *et al*. (2010)[4]
PIP5K1B	rs4744712	9	71,434,707	A	C	-0.753	0.112	3.3×10^{-11}	71,638	Kottgen *et al*. (2010)[4]

WDR37	rs10794720	10	1,156,165	T	C	-0.664	0.182	0.00033	71,638	Kottgen *et al*. (2010)[4]
A1CF	rs10994860	10	52,645,424	C	T	-0.322	0.177	0.072	61,866	Pattaro *et al*. (2016)[8]
KCNQ1	rs163160	11	2,789,955	G	A	-0.557	0.148	0.00021	71,638	Pattaro *et al*. (2016)[8]
DCDC5-MPPED2	rs3925584	11	30,760,335	T	C	-0.647	0.121	$1.5x10^{-7}$	65,983	Pattaro *et al*. (2012)[5]
AP5B1	rs4014195	11	65,506,822	G	C	-0.289	0.122	0.021	71,638	Pattaro *et al*. (2016)[8]
SLC6A13	rs10774021	12	349,298	T	C	-0.477	0.112	$2.9x10^{-5}$	71,638	Kottgen *et al*. (2010)[4]
TSPAN9	rs10491967	12	3,368,093	A	G	-0.398	0.148	0.0080	61,867	Pattaro *et al*. (2016)[8]
PTPRO	rs7956634	12	15,321,194	T	C	-0.426	0.125	0.00076	71,638	Pattaro *et al*. (2016)[8]
INHBC	rs1106766	12	57,809,456	C	T	-0.233	0.137	0.095	71,637	Pattaro *et al*. (2016)[8]
DACH1	rs626277	13	72,347,696	A	C	-0.428	0.116	0.00027	71,638	Kottgen *et al*. (2010)[4]
INO80	rs2928148	15	41,401,550	G	A	-0.278	0.113	0.016	71,638	Pattaro *et al*. (2012)[5]
SPATA5L1-GATM	rs2453533	15	45,641,225	A	C	-0.849	0.124	$1.8x10^{-11}$	71,638	Kottgen *et al*. (2010)[4]
WDR72	rs491567	15	53,946,593	A	C	-0.639	0.120	$1.4x10^{-7}$	71,638	Kottgen *et al*. (2010)[4]
UBE2Q2	rs1394125	15	76,158,983	A	G	-0.442	0.126	0.00052	71,638	Kottgen *et al*. (2010)[4]
PDILT-UMOD	rs12917707	16	20,367,690	G	T	-1.050	0.169	$8.8x10^{-10}$	48,102	Kottgen *et al*. (2010)[4]
DPEP1	rs164748	16	89,708,292	G	C	-0.593	0.131	$8.4x10^{-6}$	71,637	Pattaro *et al*. (2016)[8]
SLC47A1	rs2453580	17	19,438,321	C	T	-0.314	0.126	0.014	71,638	Pattaro *et al*. (2012)[5]
CDK12	rs11078903	17	37,631,924	A	G	-0.564	0.125	$8.6x10^{-6}$	71,638	Pattaro *et al*. (2012)[5]
BCAS3	rs9895661	17	59,456,589	C	T	-1.003	0.132	$7.9x10^{-14}$	71,638	Kottgen *et al*. (2010)[4]
NFATC1	rs8091180	18	77,164,243	A	G	-0.415	0.131	0.0018	59,864	Pattaro *et al*. (2016)[8]
SLC7A9	rs12460786	19	20,977,663	T	C	-0.030	0.117	0.80	71,638	Kottgen *et al*. (2010)[4]
SIPA1L3	rs11666497	19	38,464,262	T	C	-0.119	0.157	0.46	71,638	Pattaro *et al*. (2016)[8]
TP53INP2	rs6088580	20	33,285,053	C	G	-0.192	0.112	0.091	71,638	Pattaro *et al*. (2016)[8]
BCAS1	rs17216707	20	52,732,362	T	C	-0.761	0.150	$5.7x10^{-7}$	71,638	Pattaro *et al*. (2016)[8]

Chr: chromosome. SE: standard error.
[a]Effect allele is eGFR decreasing allele.

Table S5. Ancestry-specific association summary statistics for eGFR for lead SNPs from the trans-ethnic meta-analysis of 71,638 individuals.

Locus	Lead SNP	Chr	Position (bp, b37)	Alleles		Ancestry group	Ancestry-specific association statistics				
				Effect	Other		EAF	Beta	SE	*p*-value	*N*
GCKR	rs1260326	2	27,730,940	C	T	AFA	0.84	-1.123	0.422	0.0078	8,224
						EAS	0.44	-0.637	0.262	0.015	23,536
						EUR	0.60	-0.830	0.158	2.0×10^{-7}	23,553
						HIS	0.66	-1.098	0.248	1.1×10^{-5}	16,325
ALMS1	rs7587577	2	73,832,786	C	T	AFA	0.48	-0.894	0.301	0.0029	8,224
						EAS	N/A	N/A	N/A	N/A	N/A
						EUR	0.76	-1.037	0.186	3.2×10^{-8}	23,553
						HIS	0.73	-0.813	0.262	0.0020	16,325
LRP2	rs57989581	2	170,194,459	C	A	AFA	0.96	-2.458	0.813	0.0025	8,224
						EAS	0.92	-1.065	0.461	0.021	23,536
						EUR	0.98	-3.084	0.667	4.4×10^{-6}	23,553
						HIS	0.98	-2.658	0.784	0.00074	16,325
CPS1	rs715	2	211,543,055	C	T	AFA	0.22	-1.180	0.386	0.022	8,224
						EAS	0.16	-1.406	0.373	0.00017	23,536
						EUR	0.32	-0.765	0.175	1.4×10^{-5}	23,553
						HIS	0.28	-0.729	0.259	0.0051	16,325
TFDP2	rs1511299	3	141,716,072	T	C	AFA	0.91	-1.214	0.523	0.022	8,224
						EAS	0.72	-0.584	0.286	0.041	23,536
						EUR	0.74	-0.746	0.177	3.0×10^{-5}	23,553
						HIS	0.83	-0.668	0.306	0.029	16,325
SHROOM3	rs52020545	4	77,414,988	T	C	AFA	0.26	-0.410	0.355	0.25	8,224
						EAS	0.16	-1.803	0.391	4.0×10^{-6}	23,536
						EUR	0.43	-1.116	0.161	6.4×10^{-12}	23,553
						HIS	0.36	-0.579	0.242	0.017	16,325
DAB2-C9	chr5:39404526:D	5	39,404,526	D	R	AFA	0.12	-1.086	0.375	0.0039	8,224
						EAS	N/A	N/A	N/A	N/A	N/A
						EUR	0.42	-0.721	0.160	7.8×10^{-6}	23,553
						HIS	0.38	-0.925	0.241	0.00013	16,325
SLC43A1	rs35716097	5	176,806,636	T	C	AFA	0.36	-0.734	0.393	0.062	8,224
						EAS	0.33	-1.905	0.324	4.2×10^{-9}	23,536
						EUR	0.30	-0.897	0.178	5.8×10^{-7}	23,553
						HIS	0.29	-1.169	0.268	1.4×10^{-5}	16,325
LOC100132354-VEGFA	rs881858	6	43,806,609	A	G	AFA	0.41	-0.335	0.318	0.29	8,224
						EAS	0.87	-0.807	0.434	0.063	23,536
						EUR	0.68	-0.632	0.175	0.00034	23,553
						HIS	0.71	-1.375	0.264	2.1×10^{-7}	16,325
SLC22A2	rs316009	6	160,675,764	C	T	AFA	0.91	-1.648	0.547	0.0026	8,224
						EAS	0.95	-1.777	0.569	0.0018	23,536
						EUR	0.90	-1.000	0.255	0.00010	23,553
						HIS	0.92	-1.123	0.437	0.010	16,325
UNCX	rs62435145	7	1,286,567	T	G	AFA	N/A	N/A	N/A	N/A	N/A
						EAS	0.32	-1.611	0.282	1.1×10^{-8}	23,536
						EUR	0.66	-0.773	0.197	0.00010	23,553
						HIS	0.49	-1.208	0.261	4.1×10^{-6}	12,776
PHTF2	rs848486	7	77,552,127	G	A	AFA	0.53	-0.665	0.298	0.026	8,224
						EAS	0.23	-0.442	0.309	0.15	23,536
						EUR	0.41	-0.600	0.160	0.00020	23,553
						HIS	0.37	-0.844	0.238	0.00041	16,325
PRKAG2	rs10265221	7	151,414,329	C	T	AFA	0.16	-1.512	0.506	0.0028	8,224
						EAS	0.07	-0.680	0.707	0.34	23,536
						EUR	0.29	-0.850	0.183	4.2×10^{-6}	23,553
						HIS	0.20	-1.117	0.295	0.00016	16,325

PIP5K1B	rs4744712	9	71,434,707	A	C	AFA	0.42	-0.828	0.303	0.0063	8,224
						EAS	0.38	-0.620	0.264	0.019	23,536
						EUR	0.40	-0.669	0.158	$2.7x10^{-5}$	23,553
						HIS	0.28	-1.055	0.261	$5.7x10^{-5}$	16,325
DCDC5-MPPED2	rs963837	11	30,749,090	T	C	AFA	0.85	-0.365	0.453	0.42	8,224
						EAS	0.65	-0.989	0.268	0.00023	23,536
						EUR	0.55	-0.572	0.158	0.00032	23,553
						HIS	0.60	0.79	0.239	0.0010	16,325
SPATA5L1-GATM	rs2486288	15	45,712,339	C	T	AFA	0.82	-1.057	0.405	0.0090	8,224
						EAS	0.94	-0.497	0.543	0.36	23,536
						EUR	0.38	-0.923	0.162	$1.5x10^{-8}$	23,553
						HIS	0.63	-0.801	0.255	0.0018	16,325
WDR72	rs1031755	15	53,951,435	A	C	AFA	0.82	-0.966	0.390	0.013	8,224
						EAS	0.60	-1.463	0.263	$2.8x10^{-8}$	23,536
						EUR	0.79	-0.963	0.193	$7.0x10^{-7}$	23,553
						HIS	0.70	-0.016	0.263	0.95	16,325
PDILT-UMOD	rs77924615	16	20,392,332	G	A	AFA	0.92	-0.446	0.651	0.49	8,224
						EAS	0.78	-1.589	0.314	$4.2x10^{-7}$	23,536
						EUR	0.80	-1.300	0.213	$1.5x10^{-9}$	23,553
						HIS	0.80	-0.779	0.289	0.0072	16,325
BCAS3	rs9895661	17	59,456,589	C	T	AFA	0.45	-0.569	0.301	0.059	8,224
						EAS	0.53	-1.491	0.309	$1.4x10^{-6}$	23,536
						EUR	0.20	-0.851	0.231	0.00026	23,553
						HIS	0.45	-1.148	0.243	$2.5x10^{-6}$	16,325
NFATC1	rs8096658	18	77,156,537	G	C	AFA	N/A	N/A	N/A	N/A	N/A
						EAS	0.29	-0.856	0.308	0.0054	23,536
						EUR	0.47	-0.632	0.196	0.0014	23,553
						HIS	0.43	-1.124	0.270	$3.4x10^{-5}$	12,776

Chr: chromosome. EAF: effect allele frequency. SE: standard error. AFA: African American. EAS: East Asian. EUR: European. HIS: Hispanic.

Table S6. Residual association signals for eGFR from the trans-ethnic meta-analysis of 71,638 individuals at each locus after adjusting for the lead SNP.

Locus	Index SNP[a]	Chr	Pos (bp, b37)	Alleles		Unconditional meta-analysis			Conditional meta-analysis		
				Effect[b]	Other	Beta	SE	*p*-value	Beta	SE	*p*-value
GCKR	rs113778329	2	27,896,643	G	A	-0.608	0.383	0.12	-1.332	0.447	0.0029
ALMS1	rs12998058	2	73,511,468	G	A	-0.125	0.172	0.47	-0.717	0.173	$3.3x10^{-5}$
LRP2	rs74648148	2	169,774,784	G	C	-0.742	0.336	0.029	-1.114	0.382	0.0035
CPS1	rs9917188	2	211,904,894	T	A	-0.552	0.249	0.029	-0.910	0.251	0.00028
TFDP2	rs58623354	3	141,550,696	T	G	-0.313	0.144	0.032	-0.548	0.145	0.00017
SHROOM3	rs62300863	4	77,399,651	C	T	-0.697	0.152	$6.4x10^{-6}$	-0.543	0.161	0.00075
DAB2-C9	rs117574694	5	39,762,051	G	T	1.183	0.337	0.00053	-1.113	0.330	0.00074
SLC34A1	rs72813176	5	176,709,333	A	G	0.145	0.248	0.57	-0.585	0.253	0.021
LOC100132354-VEGFA	rs111451988	6	43,566,036	G	A	-0.638	0.305	0.040	-1.060	0.308	0.00058
SLC22A2	rs2665355	6	160,837,368	G	C	0.120	0.109	0.28	-0.401	0.110	0.00026
UNCX	rs10282027	7	1,005,018	A	G	-0.747	0.316	0.020	-1.047	0.317	0.00095
PHTF2	rs151202634	7	77,811,782	G	A	-1.495	0.525	0.0050	-1.585	0.513	0.0020
PRKAG2	rs6464171	7	151,505,876	C	G	-0.099	0.127	0.44	-0.388	0.127	0.0023
PIP5K1B	rs75852340	9	71,164,514	C	G	-1.916	0.564	0.00081	-1.877	0.661	0.0046
DCDC5-MPPED2	rs1813133	11	31,243,672	C	T	-0.523	0.340	0.13	-1.191	0.344	0.00054
SPATA5L1-GATM	rs140661904	15	46,041,594	A	T	-1.567	0.441	0.00047	-1.551	0.536	0.0038
WDR72	rs1878189	15	53,786,594	C	G	-1.015	0.282	0.00040	-1.108	0.302	0.00025
PDILT-UMOD	rs9928757	16	20,352,863	G	C	-1.012	0.165	$1.5x10^{-9}$	-0.677	0.180	0.00018
BCAS3	rs79068244	17	59,217,958	C	T	1.542	0.389	$9.3x10^{-5}$	-1.629	0.380	$1.8x10^{-5}$
NFATC1	rs526317	18	77,546,641	A	G	-0.676	0.198	0.00077	-0.549	0.186	0.0032

Chr: chromosome. SE: standard error.

[a]Index SNP has strongest residual signal of association across the locus in trans-ethnic meta-analysis after adjusting for lead SNP.

[b]Effect allele is eGFR decreasing allele in conditional meta-analysis.

Table S7. Association summary statistics for CKD at lead eGFR SNPs from the trans-ethnic meta-analysis of up to 3,976 cases and 55,904 controls.

Locus	Lead eGFR SNP	Chr	Position (bp, b37)	Alleles		CKD association summary statistics			Sample size: cases/controls
				Effect[a]	Other	OR (95% CI)	*p*-value	Cochran's *Q* *p*-value	
GCKR	rs1260326	2	27,730,940	C	T	1.04 (0.99-1.09)	0.16	0.0047	3,976/55,904
ALMS1	rs7587577	2	73,832,786	C	T	1.17 (1.09-1.24)	$3.1x10^{-6}$	0.29	2,646/33,698
LRP2	rs57989581	2	170,194,459	C	A	1.10 (0.98-1.24)	0.11	0.29	3,976/55,904
CPS1	rs715	2	211,543,055	C	T	1.06 (1.00-1.12)	0.069	0.38	3,976/55,904
TFDP2	rs1511299	3	141,716,072	T	C	1.06 (1.00-1.12)	0.068	0.39	3,976/55,904
SHROOM3	rs5020545	4	77,414,988	T	C	1.05 (1.00-1.12)	0.064	0.22	3,976/55,904
DAB2-C9	chr5:39404526:D	5	39,404,526	D	R	1.09 (1.02-1.16)	0.0084	0.63	2,646/33,698
SLC34A1	rs35716097	5	176,806,636	T	C	1.10 (1.04-1.16)	0.0011	0.84	3,976/55,904
LOC100132354-VEGFA	rs881858	6	43,806,609	A	G	1.06 (1.00-1.12)	0.057	0.010	3,976/55,904
SLC22A2	rs316009	6	160,675,764	C	T	1.13 (1.03-1.24)	0.0089	0.52	3,976/55,904
UNCX	rs62435145	7	1,286,567	T	G	1.18 (1.11-1.25)	$2.2x10^{-7}$	0.27	2,599/45,508
PHTF2	rs848486	7	77,552,127	G	A	1.03 (0.98-1.08)	0.31	0.29	3,976/55,904
PRKAG2	rs10265221	7	151,414,329	C	T	1.09 (1.02-1.16)	0.023	0.43	3,976/55,904
PIP5K1B	rs4744712	9	71,434,707	A	C	1.06 (1.01-1.11)	0.016	0.78	3,976/55,904
DCDC5-MPPED2	rs963837	11	30,749,090	T	C	1.04 (0.99-1.10)	0.15	0.49	3,976/55,904
SPATA5L1-GATM	rs2486288	15	45,712,339	C	T	1.09 (1.03-1.16)	0.0049	0.098	3,976/55,904
WDR72	rs1031755	15	53,951,435	A	C	1.09 (1.03-1.15)	0.0033	0.99	3,976/55,904
PDILT-UMOD	rs77924615	16	20,392,332	G	A	1.18 (1.10-1.26)	$4.0x10^{-6}$	0.11	3,976/55,904
BCAS3	rs9895661	17	59,456,589	C	T	1.06 (1.01-1.12)	0.020	0.70	3,976/55,904
NFATC1	rs8096658	18	77,156,537	G	C	1.07 (1.00-1.14)	0.040	0.13	2,599/45,508

Chr: chromosome. OR: odds ratio. CI: confidence interval.
[a]Effect allele is eGFR decreasing allele.

Table S8. Association summary statistics for eGFR for lead SNPs in 3,961/9,197 type 1/2 diabetes cases, all of European ancestry, from the SUMMIT Consortium.

Locus	Lead SNP	Chr	Position (bp, b37)	Alleles		Type 1 diabetes cases			Type 2 diabetes cases			All diabetes cases combined			
				Effect[a]	Other	Beta (SE)	*p*-value	*N*	Beta (SE)	*p*-value	*N*	Beta (SE)	*p*-value	Cochran's *Q p*-value	*N*
GCKR	rs1260326	2	27,730,940	C	T	-0.768 (0.618)	0.21	3,961	-0.204 (0.543)	0.71	9,197	-0.450 (0.408)	0.27	0.49	13,158
ALMS1	rs7587577	2	73,832,786	C	T	-0.977 (0.746)	0.19	3,961	-1.472 (0.611)	0.016	9,197	-1.273 (0.473)	0.0071	0.61	13,158
LRP2	rs57989581	2	170,194,459	C	A	-8.085 (4.266)	0.058	1,313	-3.417 (2.127)	0.11	9,197	-4.346 (1.904)	0.022	0.33	10,510
CPS1	rs715	2	211,543,055	C	T	-0.292 (0.662)	0.66	3,961	0.517 (0.571)	0.37	9,197	0.172 (0.432)	0.69	0.35	13,158
TFDP2	rs1511299	3	141,716,072	T	C	0.019 (0.701)	0.98	3,961	-0.228 (0.585)	0.70	9,197	-0.126 (0.449)	0.78	0.79	13,158
SHROOM3	rs5020545	4	77,414,988	T	C	-1.051 (0.616)	0.088	3,961	-0.777 (0.522)	0.14	9,197	-0.892 (0.398)	0.025	0.73	13,158
DAB2-C9	chr5:39404526:D	5	39,404,526	D	R	-1.165 (0.615)	0.058	3,961	0.363 (0.532)	0.49	9,197	-0.291 (0.402)	0.47	0.060	13,158
SLC34A1	rs35716097	5	176,806,636	T	C	-0.876 (0.658)	0.18	3,961	-0.507 (0.584)	0.39	9,197	-0.669 (0.437)	0.13	0.68	13,158
LOC100132354-VEGFA	rs881858	6	43,806,609	A	G	-0.356 (0.656)	0.59	3,961	-0.809 (0.557)	0.15	9,197	-0.619 (0.424)	0.14	0.60	13,158
SLC22A2	rs316009	6	160,675,764	C	T	0.268 (1.075)	0.80	3,961	-1.241 (0.827)	0.13	9,197	-0.680 (0.655)	0.30	0.27	13,158
UNCX	rs62435145	7	1,286,567	T	G	-0.884 (0.645)	0.17	3,961	-0.341 (0.600)	0.57	9,197	-0.593 (0.440)	0.18	0.54	13,158
PHTF2	rs848486	7	77,552,127	G	A	0.076 (0.616)	0.90	3,961	-0.260 (0.531)	0.62	9,197	-0.117 (0.402)	0.77	0.68	13,158
PRKAG2	rs10265221	7	151,414,329	C	T	-1.273 (0.731)	0.082	3,961	-2.129 (0.604)	0.00043	9,197	-1.782 (0.466)	0.00013	0.37	13,158
PIP5K1B	rs4744712	9	71,434,707	A	C	-0.640 (1.003)	0.52	1,313	0.475 (0.536)	0.38	9,197	-0.227 (0.473)	0.63	0.33	10,510
DCDC5-MPPED2	rs963837	11	30,749,090	T	C	-0.336 (0.614)	0.58	3,961	-0.872 (0.543)	0.11	9,197	-0.637 (0.407)	0.12	0.51	13,158
SPATA5L1-GATM	rs2486288	15	45,712,339	C	T	-0.711 (0.617)	0.25	3,961	-1.159 (0.544)	0.033	9,197	-0.963 (0.408)	0.018	0.59	13,158
WDR72	rs1031755	15	53,951,435	A	C	1.758 (0.702)	0.012	3,961	-1.018 (0.627)	0.10	9,197	0.212 (0.468)	0.65	0.0032	13,158
PDILT-UMOD	rs77924615	16	20,392,332	G	A	-1.405 (0.760)	0.064	3,961	-2.915 (0.668)	1.3×10^{-5}	9,197	-2.256 (0.502)	6.9×10^{-6}	0.14	13,158
BCAS3	rs9895661	17	59,456,589	C	T	-0.943 (0.771)	0.22	3,961	-0.471 (0.723)	0.51	9,197	-0.692 (0.527)	0.19	0.66	13,158
NFATC1	rs8096658	18	77,156,537	G	C	-0.617 (0.630)	0.33	3,961	-2.235 (0.578)	0.00011	9,197	-1.495 (0.426)	0.00045	0.058	13,158

Chr: chromosome. SE: standard error.

[a]Effect allele is eGFR decreasing allele from trans-ethnic meta-analysis.

Table S9. Properties of 99% credible sets of variants at eGFR loci on the basis of trans-ethnic meta-analysis of 71,638 individuals.

Locus	Lead SNP	Chr	Position (bp, b37)	99% credible set		
				SNPs	Distance (bp)	Interval (bp, b37)
GCKR	rs1260326	2	27,730,940	3	11,664	27,730,940-27,742,603
ALMS1	rs7587577	2	73,832,786	159	278,238	73,622,663-73,900,900
LRP2	rs57989581	2	170,194,459	6	10,315	170,194,459-170,204,773
CPS1	rs715	2	211,543,055	9	40,636	211,540,507-211,581,142
TFDP2	rs1511299	3	141,716,072	123	221,865	141,637,438-141,859,302
SHROOM3	rs5020545	4	77,414,988	6	20,971	77,394,018-77,414,988
DAB2-C9	chr5:39404526:D	5	39,404,526	31	68,620	39,359,773-39,428,392
SLC34A1	rs35716097	5	176,806,636	2	562	176,806,636-176,807,197
LOC100132354-VEGFA	rs881858	6	43,806,609	16	14,135	43,804,808-43,818,942
SLC22A2	rs316009	6	160,675,764	99	126,912	160,631,670-160,758,581
UNCX	rs62435145	7	1,286,567	7	11,947	1,281,064-1,293,010
PHTF2	rs848486	7	77,552,127	180	478,315	77,112,367-77,590,681
PRKAG2	rs10265221	7	151,414,329	13	9,719	151,405,818-151,415,536
PIP5K1B	rs4744712	9	71,434,707	5	3,534	71,431,174-71,434,707
DCDC5-MPPED2	rs963837	11	30,749,090	4	27,925	30,749,090-30,777,014
SPATA5L1-GATM	rs2486288	15	45,712,339	49	114,098	45,614,502-45,728,599
WDR72	rs1031755	15	53,951,435	20	49,581	53,915,766-53,965,346
PDILT-UMOD	rs77924615	16	20,392,332	1	1	20,392,332-20,393,332
BCAS3	rs9895661	17	59,456,589	6	22,488	59,449,636-59,472,123
NFATC1	rs8096658	18	77,156,537	2	435	77,156,103-77,156,537

Chr: chromosome.

Table S10. Membership of 99% credible sets containing no more than five variants on the basis of MANTRA trans-ethnic fine-mapping analysis of 71,638 individuals.

Locus	Lead SNP	99% credible set										
		Variant	Chr	Position (bp, b37)	Effect allele[a]	Other allele	Beta	SE	*p*-value	*N*	$\log_{10}$BF	Posterior probability π_C
GCKR	rs1260326	rs1260326	2	27,730,940	C	T	-0.872	0.114	6.1×10^{-14}	71,638	12.23	0.938
		rs780094	2	27,741,237	C	T	-0.810	0.113	2.0×10^{-12}	71,638	10.59	0.021
		rs780093	2	27,742,603	C	T	-0.821	0.114	1.3×10^{-12}	71,638	10.84	0.038
SLC34A1	rs35716097	rs35716097	5	176,806,636	T	C	-1.097	0.127	2.2×10^{-17}	71,638	15.92	0.946
		rs12659266	5	176,807,197	T	C	-1.109	0.134	4.3×10^{-16}	71,638	14.60	0.045
PIP5K1B	rs4744712	rs7042786	9	71,431,174	A	T	-0.727	0.113	2.1×10^{-10}	71,637	8.36	0.117
		rs2039424	9	71,432,174	G	A	-0.689	0.113	2.0×10^{-9}	71,638	7.57	0.019
		rs1556751	9	71,433,212	G	A	-0.666	0.113	7.0×10^{-9}	71,638	6.78	0.003
		rs10746942	9	71,434,465	G	A	-0.688	0.114	2.5×10^{-9}	71,637	7.54	0.018
		rs4744712	9	71,434,707	A	C	-0.753	0.112	3.3×10^{-11}	71,638	9.21	0.835
DCDC5-MPPED2	rs963837	rs963837	11	30,749,090	T	C	-0.685	0.114	3.7×10^{-9}	71,638	7.37	0.920
		rs3925584	11	30,760,335	T	C	-0.647	0.121	1.5×10^{-7}	65,983	5.84	0.027
		rs10767873	11	30,768,678	C	T	-0.628	0.115	8.2×10^{-8}	71,638	6.04	0.043
		chr11:30777014:I	11	30,777,014	R	I	-0.656	0.130	6.4×10^{-7}	48,102	5.24	0.007
PDILT-UMOD	rs77924615	rs77924615	16	20,392,332	G	A	-1.185	0.147	1.7×10^{-15}	71,638	14.23	1.000
NFATC1	rs8096658	rs71359461	18	77,156,103	C	G	-0.786	0.146	1.2×10^{-7}	59,864	5.95	0.113
		rs8096658	18	77,156,537	G	C	-0.814	0.141	1.3×10^{-8}	59,864	6.84	0.876
		rs138901831	18	77,160,067	G	C	-0.827	0.169	1.5×10^{-6}	59,864	4.78	0.008

Chr: chromosome. SE: standard error.
[a]Effect allele is eGFR decreasing allele.

Table S11. Posterior probability of driving eGFR association signals across for each single nucleotide variant annotation.

Annotation[a]	Number of single nucleotide variants	Posterior probability of driving association signals	
		Total	Percentage
Missense	317	1.04	5.39
5' UTR	249	0.14	0.73
3' UTR	709	1.02	5.29
Downstream	2099	0.3	1.56
Upstream	2473	0.12	0.62
Intronic	32384	12.12	62.83
Intergenic	13354	2.83	14.67
Non-coding transcript	1135	0.97	5.03
Others	1661	0.75	3.89

[a]Annotations were prioritised by considering the most severe consequence of all those reported for each variant.

Table S12. Genomic annotations of regulatory chromatin state from 93 cell types, Dnase I hypersensitivity sites from 145 cell types (DHS), and chromatin immuno-precipitation binding sites for 165 proteins (TF ChIP-seq) that were predictive of posterior probability of driving eGFR association signals (*p*<0.00012, Bonferroni correction for 403 annotations).

Annotation	Description	Logistic regression model			fGWAS
		Effect	SE	*p*-value	Effect (95% CI)
HDAC8	TF ChIP-seq	4.695	0.614	$1.1x10^{-14}$	6.45 (4.21-8.40)
NFE2	TF ChIP-seq	4.676	0.618	$1.9x10^{-14}$	4.72 (1.68-6.50)
FOSL1	TF ChIP-seq	3.866	0.558	$2.1x10^{-12}$	4.40 (2.40-5.80)
RPTEC	Renal epithelial DHS	2.194	0.407	$3.4x10^{-8}$	3.37 (2.25-4.42)
HRCE	Renal epithelial DHS	2.135	0.436	$4.7x10^{-7}$	3.11 (1.96-4.17)
ATF3	TF ChIP-seq	3.010	0.648	$1.7x10^{-6}$	3.66 (1.64-5.01)
fKidney_renal_cortex_L	Fetal kidney DHS	1.847	0.430	$8.8x10^{-6}$	2.76 (1.62-3.82)
fKidney_L	Fetal kidney DHS	1.881	0.446	$1.2x10^{-5}$	3.22 (2.05-4.36)
fKidney_R	Fetal kidney DHS	1.986	0.475	$1.4x10^{-5}$	3.41 (2.25-4.60)
IRF4	TF ChIP-seq	3.069	0.749	$2.1x10^{-5}$	3.84 (0.95-5.40)
fIntestine_Lg	Fetal intestine DHS	2.088	0.512	$2.3x10^{-5}$	2.96 (1.70-4.03)
fKidney_renal_pelvis	Fetal kidney DHS	1.884	0.465	$2.5x10^{-5}$	3.30 (2.15-4.22)
fKidney_renal_pelvis_L	Fetal kidney DHS	1.932	0.489	$3.9x10^{-5}$	3.50 (2.35-4.64)
MAFK	TF ChIP-seq	2.375	0.603	$4.1x10^{-5}$	3.03 (1.06-4.29)
HRE	Renal epithelial DHS	1.903	0.501	$7.4x10^{-5}$	2.87 (1.56-3.97)
fSkin	Fetal skin DHS	1.956	0.523	$9.2x10^{-5}$	3.40 (2.22-4.45)

SE: standard error. CI: confidence interval.

Table S13. Variants with more than 80% posterior probability of driving eGFR association signals that overlap with enriched regulatory annotations and their impact on expression of most correlated gene in GTEx database.

Locus	Lead SNP	Posterior probability π_C	Overlap with enriched regulatory annotations	Expression quantitative trait loci reported in GTEx database		
				Tissue	Gene	*p*-value
PDILT-UMOD	rs77924615	1.000	fKidney_R, RPTEC, fKidney_L, fKidney_renal_pelvis_L, fKidney_renal_pelvis, fKidney_renal_pelvis_R, HRCE			
SLC34A1	rs35716097	0.946	RPTEC, HRCE, HDAC8	Adipose_Subcutaneous	*RGS14*	4.1×10^{-15}
				Adrenal_Gland	*RGS14*	1.1×10^{-11}
				Artery_Aorta	*RGS14*	6.4×10^{-21}
				Artery_Coronary	*RGS14*	2.8×10^{-8}
				Artery_Tibial	*RGS14*	2.5×10^{-28}
				Brain_Cerebellum	*RGS14*	1.9×10^{-9}
				Breast_Mammary_Tissue	*RGS14*	1.1×10^{-8}
				Cells_Transformed_fibroblasts	*RGS14*	1.9×10^{-45}
				Colon_Sigmoid	*RGS14*	9.3×10^{-7}
				Colon_Transverse	*RGS14*	1.1×10^{-14}
				Esophagus_Mucosa	*RGS14*	5.2×10^{-18}
				Esophagus_Muscularis	*RGS14*	5.6×10^{-13}
				Heart_Atrial_Appendage	*RGS14*	3.9×10^{-13}
				Heart_Left_Ventricle	*RGS14*	1.4×10^{-18}
				Lung	*RGS14*	3.9×10^{-11}
				Muscle_Skeletal	*RGS14*	1.3×10^{-12}
				Nerve_Tibial	*RGS14*	6.1×10^{-15}
				Pancreas	*RGS14*	1.4×10^{-6}
				Pituitary	*RGS14*	3.0×10^{-13}
				Skin_Not_Sun_Exposed_Suprapubic	*RGS14*	2.1×10^{-8}
				Skin_Sun_Exposed_Lower_leg	*RGS14*	5.9×10^{-18}
				Stomach	*RGS14*	1.0×10^{-11}
				Testis	*RGS14*	1.5×10^{-27}
				Thyroid	*RGS14*	7.8×10^{-15}
DCDC5- MPPED2	rs963837	0.920	fKidney_R, fKidney_renal_pelvis_L			
NFATC1	rs8096658	0.877	fKidney_R, fKidney_L, fKidney_renal_pelvis, fKidney_renal_pelvis_R, fKidney_renal_pelvis_L	Heart_Left_Ventricle	*NFATC1*	2.4×10^{-9}
				Muscle_Skeletal	*NFATC1*	2.8×10^{-21}

PIP5K1B	rs4744712	0.835	fKidney_renal_pelvis	Artery_Aorta	*PIP5K1B*	$3.6x10^{-6}$
				Artery_Tibial	*PIP5K1B*	$3.6x10^{-14}$
				Testis	*PIP5K1B*	$1.9x10^{-6}$

Table S14. Estimated effects from the Cox proportional hazards model with robust standard errors, applied on the experimental *Drosophila melanogaster* survival data under isogenic and heterogenic conditions.

(a) Isogenic background

**Mutation**	**NaCl concentration**	**Log-hazard ratio**	**Robust SE**	***p*-value**
d06164	0.2	-1.4	0.32	$2.2x10^{-5}$
	0.3	-2.4	0.20	$<10^{-16}$
	0.4	-2.7	0.16	$<10^{-16}$
	0.5	-2.2	0.17	$<10^{-16}$
EY-P283	0.2	-0.29	0.75	0.21
	0.3	-1.9	0.22	$<10^{-16}$
	0.4	-2.3	0.17	$<10^{-16}$
	0.5	-1.7	0.18	$<10^{-16}$

(b) Heterogenic background

**Mutation**	**NaCl concentration**	**Log-hazard ratio**	**Robust SE**	***p*-value**
d06164	0.2	-2.1	0.46	$6.9x10^{-6}$
	0.3	-1.9	0.31	$6.5x10^{-10}$
	0.4	-1.8	0.22	$1.1x10^{-16}$
	0.5	-1.4	0.23	$3.6x10^{-10}$
EY-P283	0.2	-1.6	0.48	0.00078
	0.3	-2.3	0.32	$2.0x10^{-12}$
	0.4	-0.18	0.17	0.30
	0.5	-0.042	0.21	0.84

SE: standard error

ADDITIONAL ACKNOWLEDGEMENTS

AUSTWIN. We acknowledge funding from the Australian National Health and Medical Research Council (241944, 389875, 389891,389892, 389938, 442915, 442981, 496739 and 552485), US National Institutes of Health (AA07535, AA10248 and AA014041) and the Australian Research Council (DP0770096).

BBJ. The BioBank Japan Project was supported by the Ministry of Education, Culture, Sports, Sciences and Technology of the Japanese government.

HCHS/SOL. The Hispanic Community Health Study / Study of Latinos was carried out as a collaborative study supported by contracts from the National Heart, Lung, and Blood Institute (NHLBI) to the University of North Carolina (N01-HC65233), University of Miami (N01-HC65234), Albert Einstein College of Medicine (N01-HC65235), Northwestern University (N01-HC65236), and San Diego State University (N01-HC65237). The following contribute to the HCHS/SOL through a transfer of funds to the NHLBI: National Center on Minority Health and Health Disparities, the National Institute of Deafness and Other Communications Disorders, the National Institute of Dental and Craniofacial Research (NIDCR), the National Institute of Diabetes and Digestive and Kidney Diseases, the National Institute of Neurological Disorders and Stroke, and the Office of Dietary Supplements. The Genetic Analysis Center at the University of Washington was supported by NHLBI and NIDCR contracts (HHSN268201300005C AM03 and MOD03).

PIVUS and ULSAM. These projects were supported by Knut and Alice Wallenberg Foundation (Wallenberg Academy Fellow), European Research Council (ERC Starting Grant), Swedish Diabetes Foundation (2013-024), Swedish Research Council (2012-1397, 2012-1727, and 2012-2215), Marianne and Marcus Wallenberg Foundation, County Council of Dalarna, Dalarna University, and Swedish Heart-Lung Foundation (20120197). The computations were performed on resources provided by SNIC through Uppsala Multidisciplinary Center for Advanced Computational Science (UPPMAX) under Project b2011036. Genotyping was funded by the Wellcome Trust under award WT064890. Analysis of genetic data was funded by the Wellcome Trust under awards WT098017 and WT090532. We thank the SNP&SEQ Technology Platform in Uppsala (www.genotyping.se) for excellent genotyping.

WHI. The Women's Health Initiative program is funded by the National Heart, Lung, and Blood Institute, National Institutes of Health, US Department of Health and Human Services, through contracts HHSN268201100046C, HHSN268201100001C, HHSN268201100002C, HHSN268201100003C, HHSN268201100004C, and HHSN271201100004C.
