# Supplementary material for: Trans-ethnic Fine Mapping Highlights Kidney-Function Genes Linked to Salt Sensitivity
Source: Am J Hum Genet. 2016 Sep 1;99(3):636–46. doi: 10.1016/j.ajhg.2016.07.012 (PMC5011075; doi:10.1016/j.ajhg.2016.07.012)
Supplement: Document S2. Article plus Supplemental Data [file mmc2.pdf]

ARTICLE

Trans-ethnic Fine Mapping Highlights Kidney-Function Genes Linked to Salt Sensitivity

Anubha Mahajan,[1] Aylin R. Rodan,[2] Thu H. Le,[3] Kyle J. Gaulton,[4] Jeffrey Haessler,[5] Adrienne M. Stilp,[6] Yoichiro Kamatani,[7] Gu Zhu,[8] Tamar Sofer,[6] Sanjana Puri,[2] Jeffrey N. Schellinger,[2] Pei-Lun Chu,[3] Sylvia Cechova,[3] Natalie van Zuydam,[1] the SUMMIT Consortium, the BioBank Japan Project, Johan Arnlov,[9,10] Michael F. Flessner,[11] Vilmantas Giedraitis,[12] Andrew C. Heath,[13] Michiaki Kubo,[14] Anders Larsson,[9] Cecilia M. Lindgren,[1,15] Pamela A.F. Madden,[13] Grant W. Montgomery,[16] George J. Papanicolaou,[17] Alex P. Reiner,[5] Johan Sundström,[9] Timothy A. Thornton,[6] Lars Lind,[9] Erik Ingelsson,[18,19] Jianwen Cai,[20] Nicholas G. Martin,[8] Charles Kooperberg,[5] Koichi Matsuda,[21] John B. Whitfield,[8] Yukinori Okada,[7,22] Cathy C. Laurie,[6] Andrew P. Morris,[1,23,25,*] and Nora Franceschini[24,25,*]

We analyzed genome-wide association studies (GWASs), including data from 71,638 individuals from four ancestries, for estimated glomerular filtration rate (eGFR), a measure of kidney function used to define chronic kidney disease (CKD). We identified 20 loci attaining genome-wide-significant evidence of association ($p < 5 \times 10^{-8}$) with kidney function and highlighted that allelic effects on eGFR at lead SNPs are homogeneous across ancestries. We leveraged differences in the pattern of linkage disequilibrium between diverse populations to fine-map the 20 loci through construction of "credible sets" of variants driving eGFR association signals. Credible variants at the 20 eGFR loci were enriched for DNase I hypersensitivity sites (DHSs) in human kidney cells. DHS credible variants were expression quantitative trait loci for *NFATC1* and *RGS14* (at the *SLC34A1* locus) in multiple tissues. Loss-of-function mutations in ancestral orthologs of both genes in *Drosophila melanogaster* were associated with altered sensitivity to salt stress. Renal mRNA expression of *Nfatc1* and *Rgs14* in a salt-sensitive mouse model was also reduced after exposure to a high-salt diet or induced CKD. Our study (1) demonstrates the utility of trans-ethnic fine mapping through integration of GWASs involving diverse populations with genomic annotation from relevant tissues to define molecular mechanisms by which association signals exert their effect and (2) suggests that salt sensitivity might be an important marker for biological processes that affect kidney function and CKD in humans.

Introduction

Chronic kidney disease (CKD) is a major public health burden and affects nearly 10% of the global population.[1] Reduced estimated glomerular filtration rate (eGFR), a measure of kidney function used to define CKD, is associated with premature cardiovascular disease and mortality, acute kidney injury, and progression to end stage renal disease (ESRD).[2] Although individuals of African and Hispanic descent suffer the largest burden of CKD,[3] the largest genome-wide association studies (GWASs) to search for kidney-function loci have been undertaken in populations of European and East Asian ancestry.[4–8] Many of these loci are characterized by common variant association signals that map to large genomic intervals, which contain many possible causal genes for eGFR, thereby limiting understanding of the downstream pathogenesis of CKD.

To address this challenge, we have undertaken a trans-ethnic meta-analysis of nine GWASs comprising 71,638 individuals from four ancestries (African American, Hispanic, European, and East Asian), each imputed up to the phase 1 integrated (March 2012 release) multi-ethnic reference panel from the 1000 Genomes Project[9], from the Continental Origins and Genetic Epidemiology

[1]Wellcome Trust Centre for Human Genetics, University of Oxford, Oxford OX3 7BN, UK; [2]Department of Internal Medicine, University of Texas Southwestern Medical Center, Dallas, TX 75229, USA; [3]Department of Medicine, University of Virginia, Charlottesville, VA 22908, USA; [4]Department of Pediatrics, University of California San Diego, La Jolla, CA 92093, USA; [5]Division of Public Health Sciences, Fred Hutchinson Cancer Research Center, Seattle, WA 98109, USA; [6]Department of Biostatistics, University of Washington, Seattle, WA 98195, USA; [7]Laboratory for Statistical Analysis, RIKEN Center for Integrative Medical Sciences, Yokohama 230-0045, Japan; [8]Genetic Epidemiology Laboratory, QIMR Berghofer Medical Research Institute, Brisbane 4006, Australia; [9]Department of Medical Sciences, Cardiovascular Epidemiology, Uppsala University, Uppsala 751 85, Sweden; [10]School of Health and Social Studies, Dalarna University, Falun 791 88, Sweden; [11]National Institute of Diabetes, Digestive, and Kidney Disease, NIH, Bethesda, MD 20892, USA; [12]Department of Public Health and Caring Sciences, Molecular Geriatrics, Uppsala University, Uppsala 752 37, Sweden; [13]Department of Psychiatry, Washington University in St. Louis, St. Louis, MO 63110, USA; [14]Laboratory for Genotyping Development, RIKEN Center for Integrative Medical Sciences, Yokohama 230-0045, Japan; [15]Big Data Institute, Li Ka Shing Centre for Health Information and Discovery, University of Oxford, Oxford OX3 7BN, UK; [16]Molecular Epidemiology Laboratory, QIMR Berghofer Medical Research Institute, Brisbane 4006, Australia; [17]Epidemiology Branch, Division of Cardiovascular Sciences, National Heart, Lung and Blood Institute, Bethesda, MD 20892, USA; [18]Department of Medical Sciences, Molecular Epidemiology and Science for Life Laboratory, Uppsala University, Uppsala 752 37, Sweden; [19]Department of Medicine, Division of Cardiovascular Medicine, Stanford University School of Medicine, Stanford, CA 94305, USA; [20]Collaborative Studies Coordinating Center, Department of Biostatistics, University of North Carolina at Chapel Hill, Chapel Hill, NC 27599, USA; [21]Laboratory of Molecular Medicine, Human Genome Center, Institute of Medical Science, University of Tokyo, Tokyo 108-8639, Japan; [22]Department of Statistical Genetics, Osaka University Graduate School of Medicine, Osaka 565-0871, Japan; [23]Department of Biostatistics, University of Liverpool, Liverpool L69 3GL, UK; [24]Department of Epidemiology, University of North Carolina, Chapel Hill, NC 27514, USA
[25]These authors contributed equally to this work
*Correspondence: noraf@unc.edu (N.F.), apmorris@liverpool.ac.uk (A.P.M.)
http://dx.doi.org/10.1016/j.ajhg.2016.07.012.
© 2016 The Author(s). This is an open access article under the CC BY license (http://creativecommons.org/licenses/by/4.0/).

Network (COGENT)-Kidney consortium. With these data, we aimed to (1) assess the evidence for heterogeneity in allelic effects on eGFR for lead SNPs at kidney-function loci across ethnic groups; (2) fine-map these loci by taking advantage of high-density imputation and by leveraging differences in the pattern of linkage disequilibrium (LD) between diverse populations to localize "credible sets" of variants driving eGFR association signals; (3) define potential molecular mechanisms through which eGFR association signals at these loci impact kidney function through overlap of credible variants with genomic annotation; and (4) assess possible markers for biological processes that impact kidney function and CKD in humans through targeted experimentation in model organisms.

Subjects and Methods

Ethics Statement

All human research was approved by the relevant institutional review boards and conducted according to the Declaration of Helsinki. All participants provided written informed consent.

Study Overview

We aggregated five GWASs of individuals of European ancestry (23,553 individuals from Europe, the USA, and Australia), two GWASs of Hispanic Americans (16,325 individuals from the USA), one GWAS of individuals of East Asian ancestry (23,536 individuals from Japan), and one GWAS of African Americans (8,224 individuals from the USA). Study sample characteristics are presented in Table S1.

Genotyping, Quality Control, and Imputation

Samples were genotyped with a variety of GWAS arrays, and quality control was undertaken within each study (Table S2). Sample quality control included exclusions on the basis of genome-wide call rate, extreme heterozygosity, sex discordance, cryptic relatedness, and outlying ethnicity. SNP quality control included exclusions on the basis of call rate across samples and extreme deviation from Hardy-Weinberg equilibrium. Non-autosomal SNPs were excluded from imputation and association analysis.

Within each study, the autosomal GWAS genotype scaffold was first pre-phased[10,11] with genetic maps from the International HapMap Consortium[12] to model recombination rates. The scaffold was then imputed up to the phase 1 integrated (March 2012 release) multi-ethnic reference panel from the 1000 Genomes Project[9] via IMPUTE2[11,13] or MaCH/Minimac[11] (Table S2). Imputed variants were retained for downstream association analyses if they attained established GWAS quality control thresholds:[14] IMPUTE2 info ≥ 0.4 or MaCH/Minimac $r^2 \geq 0.3$.

Calculation of eGFR and Association Analysis

Within each study, eGFR was calculated from serum creatinine (mg/dL), with adjustment for age, sex, and ethnicity by means of the four-variable MDRD (modification of diet in renal disease) equation[15] to be comparable with published GWASs of kidney function.[4–8] Within each study, we tested association of eGFR with each variant passing quality control in a linear regression framework under an additive dosage model and with adjustment for study-specific covariates to account for confounding due to population structure (Table S2). Association summary statistics were subsequently corrected in each study for residual population structure through a first round of genomic control[16] where necessary (Table S2).

Trans-ethnic Meta-analysis

Association summary statistics were combined across studies via fixed-effects meta-analysis (inverse-variance weighting) implemented in the GWAMA software.[17] Variants passing quality control in fewer than 50% of the total sample size across studies were excluded from the meta-analysis. Association summary statistics from the meta-analysis were then corrected for a second round of genomic control[16] ($\lambda_{GC} = 1.028$). Heterogeneity in allelic effects between studies at each variant was assessed by means of Cochran's Q statistic.[18] We extracted association summary statistics for eGFR from the trans-ethnic meta-analysis for previously reported lead SNPs at established GWAS loci.

LD Calculations

LD, as measured by the correlation coefficient r^2, was calculated on the basis of haplotypes in each ancestry group from the 1000 Genomes Project[9] via LDlink.[19]

Conditional Analyses

To assess the evidence for distinct association signals at each locus attaining nominal significance ($p_{COND} < 10^{-5}$, Bonferroni correction for ~5,000 variants per locus) in our trans-ethnic meta-analysis, we performed conditional analysis in a 1 Mb genomic interval flanking the lead SNP. Within each study, we tested association of eGFR with each variant passing quality control in the flanking region in a linear regression framework under an additive dosage model and with adjustment for genotypes at the lead SNP, in addition to other study-specific covariates used in unconditional analysis (Table S2). Association summary statistics were subsequently corrected in each study for residual population structure, via the same genomic control[16] correction employed for unconditional analysis (Table S2). These association summary statistics were combined across studies via fixed-effects meta-analysis (inverse-variance weighting) implemented in GWAMA.[17] Variants passing quality control in less than 50% of the total sample size across studies were excluded from the meta-analysis. Association summary statistics from the conditional meta-analysis were then corrected for a second round of genomic control,[16] making use of the same adjustment as defined in the unconditional analysis ($\lambda_{GC} = 1.028$).

Association with CKD

We defined CKD by an eGFR < 60 mL/min/1.73 m^2 (calculated with the MDRD equation defined above) and/or incidence of ESRD, if available. Any individual who was prospectively initiated on dialysis or received a kidney transplant (self-reported or obtained from medical records or registries) was defined as having ESRD. Individuals who did not develop ESRD at follow-up were considered control subjects. We considered the lead eGFR SNP identified at each locus attaining genome-wide significance in our trans-ethnic meta-analysis. Within each study, we tested association of CKD with each SNP in a logistic regression framework under an additive dosage model and with adjustment for study-specific covariates to account for confounding due to population structure (Table S2). Association summary statistics were combined across studies via fixed-effects meta-analysis (sample size

and inverse-variance weighting) implemented in METAL[20] and GWAMA.[17]

Association with eGFR in Diabetic Individuals from the SUMMIT Consortium

We considered the lead eGFR SNP at each locus attaining genome-wide significance in our trans-ethnic meta-analysis. We performed a look-up of association summary statistics for eGFR in 13,158 subjects with diabetes (9,197 with type 2 diabetes [T2D] and 3,961 with type 1 diabetes [T1D]) from five studies of individuals of European ancestry from the SUMMIT Consortium. Within each study, the outcome variable was defined as the last measured eGFR, calculated with the MDRD equation (defined above). Each study was imputed up to the phase 1 integrated (March 2012 release) multi-ethnic reference panel from the 1000 Genomes Project.[9] Estimated allelic effects on eGFR were obtained from a linear mixed model and implemented in EMMAX[21] with an empirical genetic relationship matrix, assuming an additive dosage of the minor allele and including sex, age at diabetes onset, and duration of diabetes as covariates. Association summary statistics for eGFR were combined across studies via fixed-effects meta-analysis (inverse-variance weighting) implemented in GWAMA.[17] Combined allelic effect estimates across studies were reported for T1D and T2D subjects, both separately and for all diabetic individuals combined. Heterogeneity in allelic effects between T1D and T2D subjects at each variant was assessed by means of Cochran's Q statistic,[18] as implemented in GWAMA.[17]

For lead SNPs, we tested for a difference in the allelic effect on eGFR in the general population (from our trans-ethnic meta-analysis) and in diabetic indivuduals (combined T1D and T2D from the SUMMIT Consortium) by using a two-sample Z-test.

MANTRA Fine Mapping and Credible Set Construction

We performed trans-ethnic fine mapping of each locus in a 1 Mb genomic interval flanking the lead SNP. Association summary statistics for each variant in the flanking region were combined across studies with a Bayesian hybrid of fixed- and random-effects meta-analysis, as implemented in MANTRA.[22] MANTRA allows for heterogeneity in allelic effects between ancestry groups arising as a result of differences in the structure of LD between diverse populations by assigning studies to clusters according to a Bayesian partition model of relatedness between them, defined by pairwise genome-wide mean allele frequency differences (Figure S1). MANTRA has been demonstrated, both empirically and by simulation, to improve fine-mapping resolution, as compared to either a fixed- or random-effects meta-analysis.[22–24] Variants passing quality control in less than 50% of the total sample size across studies were excluded from the fine-mapping analysis.

We calculated the posterior probability that the jth variant, π_{Cj}, is driving the association signal at each locus by

$$\pi_{Cj} = \frac{\Lambda_j}{\sum_k \Lambda_k},$$

where the summation is over all variants in the flanking interval. In this expression, Λ_j is the MANTRA Bayes factor in favor of association from the trans-ethnic meta-analysis. For each distinct association signal, a 99% credible set[25] was then constructed by (1) ranking all variants according to their Bayes factor, Λ_j, and (2) including ranked variants until their cumulative posterior probability exceeds 0.99.

Genomic Annotation

For each locus attaining genome-wide significance in our trans-ethnic meta-analysis, we obtained genomic annotations of all single-nucleotide variants in a 1 Mb interval flanking the lead SNP. We utilized the Ensembl Variant Effect Predictor (VEP, version 2.7), based on the Ensembl transcript set (version 69). By default, the VEP reports all possible annotations (transcript- and gene-specific) for each variant. We therefore prioritized annotations by considering the most severe consequence of all those reported. We then calculated the total posterior probability of driving association signals for each consequence across loci.

Regulatory Annotation

We collected genomic annotations from three sources. First, we obtained regulatory chromatin states from the Epigenome Roadmap Project[26] for 93 cell types after removing five cancer cell lines. For each cell type, we pooled enhancer (EnhA and EnhWk) and promoter (TssA and TssFlnk) elements into one annotation. Second, we obtained 145 non-redundant DNase I hypersensitivity sites (DHSs) from the ENCODE Project[27] by retaining only one dataset for cell types with multiple assayed samples. Third, we obtained chromatin immuno-precipitation sequence (ChIP-seq) binding sites for 165 transcription factors: 161 proteins from the ENCODE Project[27] and additional factors assayed in primary pancreatic islets.[28] This resulted in a total of 403 annotations for downstream enrichment analyses. For each annotation, we considered variants passing quality control and mapping within 1 Mb of the lead SNP attaining genome-wide significance in the trans-ethnic meta-analysis.

We first tested the effect of each annotation on the log odds of the posterior probability of driving eGFR association signals in a logistic regression model. For each variant, we encoded overlap with the tested annotation as a binary indicator (1 if variant overlaps annotation, 0 otherwise). The regression model also incorporated binary indicators of genic annotations as covariates, as well as a categorical variable for locus membership. Specifically,

$$\text{logit}(\pi_{Cj}) = \alpha_i L_{ij} + \beta_k x_{jk} + \gamma_{3'\text{UTR}} x_{j3'\text{UTR}} + \gamma_{5'\text{UTR}} x_{j5'\text{UTR}} + \gamma_{\text{EXON}} x_{j\text{EXON}} + \gamma_{\text{TSS}} x_{j\text{TSS}},$$

where π_{Cj} is the posterior probability that the jth variant drives the eGFR association; α_i denotes an intercept for the ith locus and L_{ij} is a binary indicator of membership of the jth variant in the ith locus; β_k denotes the effect of the kth annotation and x_{kj} is a binary indicator of overlap of the jth variant with the kth annotation; and $\gamma_{3'\ \text{UTR}}$, $\gamma_{5'\ \text{UTR}}$, γ_{EXON} and γ_{TSS} denote the effects of 3′ UTRs, 5′ UTRs, coding exons, and the region within 1 kb upstream of GENCODE transcription start site (TSS) annotations, respectively, and $x_{j3'\ \text{UTR}}$, $x_{j5'\ \text{UTR}}$, $x_{j\text{EXON}}$ and $x_{j\text{TSS}}$ are binary indicators of overlap of the jth variant with these annotations. The SE of the effect of the kth annotation, β_k, was evaluated with a robust sandwich variance estimator.

Using fGWAS software, we then tested for the effect of each annotation by using the Bayes factor in favor of association.[29] We included coding exons, 3′ UTRs, 5′ UTRs, and the region within 1 kb upstream of the TSS in the model for each annotation. We obtained the estimated effect and 95% confidence interval (CI) from this model and considered an annotation enriched if the 95% CI did not overlap zero.

Drosophila melanogaster Salt-Sensitivity Assay

Four y^1w^1 virgin females were mated with two males each of the genotypes y^1w^1/Y, y^1w^1/Y; $loco^{EY\text{-}P283}/TM3\ Sb$ or y^1w^1/Y; $loco^{d06164}$

in rearing vials on standard cornmeal/yeast/molasses food (prepared in a central kitchen at University of Texas Southwestern Medical Center). The y^1w^1 isogenic control, in which all loci had been previously homozygosed, and to which *loco* mutants had been backcrossed for six generations, were obtained from Dr. Yongkyu Park (Rutgers New Jersey Medical School).[30] Separately, to obtain highly heterozygous progeny (heterogenic), virgin females from the A.R.R. lab's *wBerlin* strain were mated with males of genotypes y^1w^1/Y, y^1w^1/Y; $loco^{EY\text{-}P283}/TM3\ Sb$ or y^1w^1/Y; $loco^{d06164}$, as above. Adults were cleared from rearing vials on rearing day eight. Ten female progeny from each vial were collected within 1–3 days of eclosion and placed on food containing various concentrations of added NaCl. Each experimental vial contained flies from a single rearing vial. The number of dead flies in each vial was counted daily. Flies were transferred to fresh medium after day five, and again after day ten for the heterogenic flies. For each concentration of experimental medium, 225 g Applied Scientific Jazz-Mix *Drosophila* Food (Fisher, cat. no. AS-153) was added to 500 mL deionized water with constant stirring. Flasks were then placed on a hot plate at 350°C with constant stirring and heated to a slow boil (about 20–25 min). The heat was then turned off, 4M NaCl was added to achieve varying concentrations of added NaCl, and total volume adjusted to 900 mL with deinonized water. Medium was dispensed in 3–4 mL aliquots in polystyrene vials. All crosses and assays were performed at room temperature (~22°C–23°C) and ambient humidity.

We estimated the effect of the mutations on salt sensitivity by applying a Cox proportional hazards model on the fly survival data. The outcome was survival time, and at the end of the follow-up period, all living flies were censored. The data for each genetic background (heterogenic or isogenic) and NaCl concentration were analyzed separately. We estimated the effect on the hazard ratio of genotype (each mutation versus control as baseline). To account for intra-vial correlation, we used robust sandwich variance estimators in a generalized estimating equation (GEE)-like model that treats members of each vial as associated with a single cluster. Analyses were performed with the R "survival" package.

Mouse Renal Expression Study

129S6 mice were purchased from Taconic Biosciences and were maintained on a 12 hr light-dark cycle with free access to standard chow and water in the animal facility of the University of Virginia. Only male mice at 12 weeks of age were used. High-salt diet (HSD, 6% NaCl) in pellets was purchased from Harlan Teklad and administered in place of normal chow for two weeks. Experiments were carried out in accordance with local and NIH guidelines. To induce CKD, mice were subjected to sub-total nephrectomy (Nx) under 1.5% isoflurane anesthesia, the right kidney was removed, and the upper branch of the two main branches of the left renal artery were ligated to impede blood supply to the upper half of the kidney as previously reported.[31]

Renal mRNA was extracted at the end of 2 weeks of HSD, or at 12 weeks after sub-total Nx. Real-time RT-PCR was performed as previously described[32] with the primers listed in Table S3. Fluorescence detection was accomplished with Sybr Green and the iCYcler system (Bio-Rad). mRNA expression was normalized against mRNA expression of the *Hprt* housekeeping gene, and the mean at baseline was used as the reference for determination of relative expression across conditions.

Results

Identification of Loci Associated with Kidney Function across Ancestry Groups

We identified 20 loci attaining genome-wide-significant evidence of association with eGFR ($p < 5 \times 10^{-8}$) in trans-ethnic meta-analysis (Table 1, Figure S2). These loci have been previously reported in ethnic-specific GWASs of individuals with European and East Asian ancestry[4–6,8] (Table S4). They include two loci discovered in a recently published meta-analysis of European ancestry GWASs: *LRP2* ([MIM: 600073] rs57989581, $p = 5.6 \times 10^{-10}$) and *NFATC1* ([MIM: 600489] rs8096658, $p = 1.3 \times 10^{-8}$). Previously reported lead SNPs at an additional 21 established kidney-function loci attained nominal evidence of association ($p < 0.05$) with eGFR, with consistent direction of effect (Table S4).

As expected, lead SNPs were common across ancestry groups at all 20 loci, with each displaying modest effects on eGFR (Table S5). Despite substantial variability in allele frequencies between ancestry groups, we observed no evidence of trans-ethnic heterogeneity in allelic effects on eGFR for any lead SNP (Table 1, Table S5). Through conditional analyses (Table S6), we observed no evidence of multiple distinct signals of association for eGFR at any locus ($p_{COND} < 10^{-5}$, Bonferroni correction for ~5,000 variants per locus). Taken together, these data are consistent with a single variant driving association signals in each locus; each variant is shared across ancestry groups and has homogeneous effects on eGFR in diverse populations. However, we recognize that larger multi-ethnic samples will be required to detect lower frequency, population-specific distinct association signals of modest effect on kidney function.

Impact of Lead eGFR SNPs on CKD and Kidney Function in Diabetic Individuals

We assessed the impact on CKD of lead SNPs at the 20 eGFR loci in a subset of individuals (up to 3,976 cases and 55,904 controls) contributing to our trans-ethnic meta-analysis (Table S7). We defined CKD by eGFR < 60 mL/min/1.73 m^2 and/or incidence of ESRD. For all 20 lead SNPs, the eGFR-decreasing allele was associated with increased risk of CKD. Eleven of the lead SNPs demonstrated evidence of association with CKD at nominal significance ($p < 0.05$), and the strongest signals were observed at *UNCX* (rs62435145, $p = 2.2 \times 10^{-7}$), *ALMS1* ([MIM: 606844] rs7587577, $p = 3.1 \times 10^{-6}$), and *PDILT-UMOD* ([MIM: 191845] rs77924615, $p = 4.0 \times 10^{-6}$).

We also investigated the impact of the lead SNPs on eGFR in GWASs of individuals with diabetes for whom there are different mechanisms for loss of renal function, such as diabetic nephropathy. We obtained association summary statistics for eGFR in 13,158 subjects of European ancestry with diabetes (9,197 with T2D and 3,961 with T1D) from the SUMMIT Consortium (Table S8). Consistent

Table 1. Loci Attaining Genome-wide-Significant Evidence of Association ($p < 5 \times 10^{-8}$) with eGFR in Trans-ethnic Meta-analysis of 71,638 Individuals

				Alleles		Trans-ethnic Meta-analysis				
Locus	Lead SNP	Chr	Position (bp, b37)	Effect[a]	Other	Beta	SE	p Value	Cochran's Q p Value	N
SLC43A1	rs35716097	5	176,806,636	T	C	−1.097	0.127	2.3×10^{-17}	0.13	71,638
SHROOM3	rs5020545	4	77,414,988	T	C	−0.969	0.119	1.3×10^{-15}	0.010	71,638
PDILT-UMOD	rs77924615	16	20,392,332	G	A	−1.185	0.147	1.7×10^{-15}	0.011	71,638
UNCX	rs62435145	7	1,286,567	T	G	−1.092	0.137	4.7×10^{-15}	0.17	59,865
GCKR	rs1260326	2	27,730,940	C	T	−0.872	0.114	6.1×10^{-14}	0.069	71,638
BCAS3	rs9895661	17	59,456,589	C	T	−1.003	0.132	7.9×10^{-14}	0.19	71,638
SPATA5L1-GATM	rs2486288	15	45,712,339	C	T	−0.883	0.126	4.7×10^{-12}	0.76	71,638
ALMS1	rs7587577	2	73,832,786	C	T	−0.948	0.135	5.2×10^{-12}	0.098	48,102
CPS1	rs715	2	211,543,055	C	T	−0.876	0.127	1.3×10^{-11}	0.21	71,638
WDR72	rs1031755	15	53,951,435	A	C	−0.860	0.127	2.2×10^{-11}	0.0013	71,638
PIP5K1B	rs4744712	9	71,434,707	A	C	−0.753	0.112	3.3×10^{-11}	0.91	71,638
PRKAG2	rs10265221	7	151,414,329	C	T	−0.963	0.146	7.3×10^{-11}	0.23	71,638
DAB2-C9	chr5: 39,404,526:D	5	39,404,526	D	R	−0.817	0.126	1.5×10^{-10}	0.80	48,102
LRP2	rs57989581	2	170,194,459	C	A	−1.980	0.315	5.6×10^{-10}	0.16	71,638
SLC22A2	rs316009	6	160,675,764	C	T	−1.193	0.192	1.0×10^{-9}	0.49	71,638
LOC100132354-VEGFA	rs881858	6	43,806,609	A	G	−0.772	0.127	2.0×10^{-9}	0.0020	71,638
DCDC5-MPPED2	rs963837	11	30,749,090	T	C	−0.685	0.114	3.7×10^{-9}	0.0034	71,638
NFATC1	rs8096658	18	77,156,537	G	C	−0.814	0.141	1.3×10^{-8}	0.015	59,865
PHTF2	rs848486	7	77,552,127	G	A	−0.643	0.113	2.0×10^{-8}	0.83	71,638
TFDP2	rs1511299	3	141,716,072	T	C	−0.727	0.131	4.4×10^{-8}	0.55	71,638

[a]Effect allele is eGFR-decreasing allele.

with previous reports,[8,33] allelic effects on eGFR in diabetic individuals and our trans-ethnic meta-analysis of individuals from the general population were homogeneous (Figure S3). There was nominal evidence of association with eGFR ($p < 0.05$), with the same direction of effect, at seven loci, and the strongest signals were observed at *PDILT-UMOD* ($p = 6.9 \times 10^{-6}$), *PRKAG2* ([MIM: 602743] $p = 0.00013$) and *NFATC1* ($p = 0.00045$).

Fine Mapping of eGFR Loci

We next sought to localize variants driving eGFR association signals in each of the 20 loci attaining genome-wide significance in our trans-ethnic meta-analysis. We utilized trans-ethnic fine mapping implemented in MANTRA,[22] taking advantage of increased sample size and the expectation that patterns of LD vary between diverse populations. We derived credible sets of variants[25] mapping within 500 kb of the lead SNP at each locus that together account for 99% of the posterior probability (π_C) of driving the association signal (Table S9). Smaller credible sets, in terms of the number of SNPs they contain, or the genomic interval that they cover, thus correspond to more precise fine-mapping. The 99% credible set at the *PDILT-UMOD* locus included a single variant (rs77924615, π_C>0.999), which maps to an intron of *PDILT*. This variant has previously been reported as driving the primary association signal for CKD at the *PDILT-UMOD* locus through whole-genome sequencing and long-range haplotype imputation into 194,286 Icelandic individuals with serum creatinine measurements.[34] We also observed precise localization, defined by a 99% credible set including no more than five variants (Table S10), at a five additional loci: *NFATC1* (two variants, mapping to 0.4 kb), *SLC34A1* ([MIM: 182309] two variants, mapping to 0.6 kb), *GCKR* ([MIM: 600842] three variants, mapping to 11.7 kb), *DCDC5-MPPED2* ([MIM: 612321, 600911] four variants, mapping to 27.9 kb), and *PIP5K1B* ([MIM: 602745] five variants, mapping to 3.5 kb).

Integration of Genetic Fine-mapping and Genomic Annotation

To gain insight into the mechanisms through which association signals at the 20 GWAS loci attaining genome-wide significance in our trans-ethnic meta-analysis impact eGFR, we began by obtaining genomic annotations for all single-nucleotide variants mapping within 500 kb of lead SNPs. Across all 20 loci, only 5.4% of the posterior

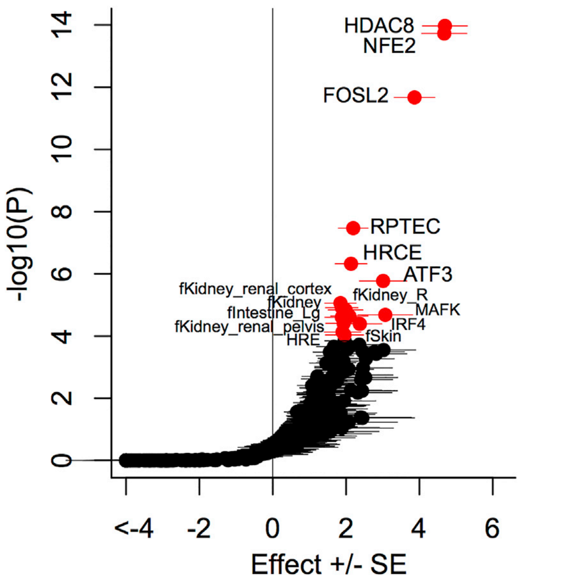

Figure 1. DNase I Hypersensitivity Sites in Kidney Cells and HDAC8 Binding Sites are Predictive of Posterior Probability of Driving Association Signals at 20 eGFR Loci
We tested whether genomic annotations of regulatory chromatin state for 93 cell types, DNase I hypersensitivity sites (DHSs) for 145 cell types, and chromatin immuno-precipitation sequence binding sites for 165 transcription factors were predictive of posterior probability of driving eGFR association signals. Each point corresponds to an annotation, plotted according to the effect size (log-odds ratio for driving association signal) on the x axis and ranked according to the significance of the association on the y axis. Significant association ($p < 0.00012$, highlighted in red) was defined by Bonferroni correction for 403 tested annotations. The most significant effects included DHSs in kidney cells (RPTECs and HRCEs) and binding sites for HDAC8.

probability of driving association signals was annotated to coding variants (Table S11), the majority of which was accounted for by *GCKR* p.Pro446Leu (rs1260326, $\pi_C = 0.938$). This missense variant has been shown, functionally, to result in increased de novo triglyceride and cholesterol synthesis and export and decreased plasma glucose concentrations, all of which have been associated with risk of CKD,[35,36] making *GCKR* the likely effector transcript for eGFR at this locus. However, outside of the *GCKR* locus, variants mapping to non-coding sequence accounted for more than 99.4% of the probability of driving eGFR association, suggesting that these signals are most likely to be mediated by effects on gene regulation.

We next investigated whether genomic annotations of regulatory chromatin state for 93 cell types,[26] DHSs for 145 cell types,[27] and ChIP-seq binding sites for 165 transcription factors[27,28] were predictive of posterior probability of driving association signals across the 20 loci (Figure 1, Table S12). We observed significant effects ($p < 0.00012$, Bonferroni correction for 403 annotations) on posterior probability for variants in kidney DHSs, including adult renal proximal tubular epithelial cells (RPTECs; $p = 3.4 \times 10^{-8}$), renal cortical epithelial cells (HRCEs; $p = 4.7 \times 10^{-7}$), and fetal kidney cells ($p = 8.8 \times 10^{-6}$). We also observed significant effects on posterior probability for transcription-factor binding sites, most notably for HDAC8 ($p = 1.1 \times 10^{-14}$). Histone deacetylases (HDACs) are involved in kidney function and development,[37] and HDAC inhibitors could be promising in the treatment of kidney disease.[38] We repeated our analyses by using fGWAS[29] (Figure S4, Table S12) and observed strong correlation in the ranking of enriched annotations ($r^2 = 0.93$). These results highlight that variants driving association signals with eGFR are more likely to be co-localized with annotated elements in kidney cells, thereby suggesting that gene regulation in disease-relevant tissues is a likely mechanism by which GWAS loci impact CKD.

Lead SNPs that, by themselves, accounted for more than 80% of the posterior probability of driving association signals overlapped an enriched annotation at five loci (Table S13). In particular, at the *SLC34A1* locus, rs35716097 ($\pi_C = 0.946$) overlapped DHSs in RPTECs and HRCEs, as well as a binding site for HDAC8, while at the *NFATC1* locus, rs8096658 ($\pi_C = 0.877$) overlapped fetal kidney cell DHSs (Figure S5). At both of these loci, the lead SNPs were also expression quantitative trait loci (eQTLs) for *NFATC1* and *RGS14* (MIM: 602513; at the *SLC34A1* locus) in multiple tissues (Table S13), highlighting these genes as likely effector transcripts through which eGFR association signals are mediated. *NFATC1* plays a central role in inducible gene transcription during immune response and is a downstream target of the transplant immunosuppression drug cyclosporine A. *RGS14* encodes a member of the regulator of G protein signaling family, which modulates downstream effects of Gα subunits and has unknown function in kidneys.

Experimental Data in Model Organisms

To provide insight into the role of *NFATC1* and *RGS14* (at the *SLC34A1* locus) in kidney physiology, we examined the function of ancestral orthologs in *Drosophila melanogaster*. The *Drosophila* genome encodes a single member of the *NFAT* family, and a previous report has demonstrated that flies with *NFAT* loss-of-function mutations have increased salt sensitivity, suggesting a role for this gene in ionic or osmotic regulation.[39] The closest *RGS14* ortholog in *Drosophila melanogaster* is *loco*, for which reduced expression is associated with longer lifespan and stress resistance.[30] We thus conducted experiments aimed at characterizing a role for *loco* loss-of-function variants in salt sensitivity. We compared survival of two independently derived heterozygous *loco* mutants (y^1w^1; $loco^{d06164}$/+ and y^1w^1; $loco^{EY\text{-}P283}$/+) with isogenic y^1w^1 controls after supplementing their diet with varying NaCl concentrations for 8 days (Figure 2). There was very little mortality of any of the genotypes on non-NaCl-supplemented food, indicating no baseline differences in viability over the time period tested. However, we observed significantly improved survival of the heterozygous *loco* mutants over controls on NaCl-supplemented food (Figure 2, Table S14), thereby indicating a role

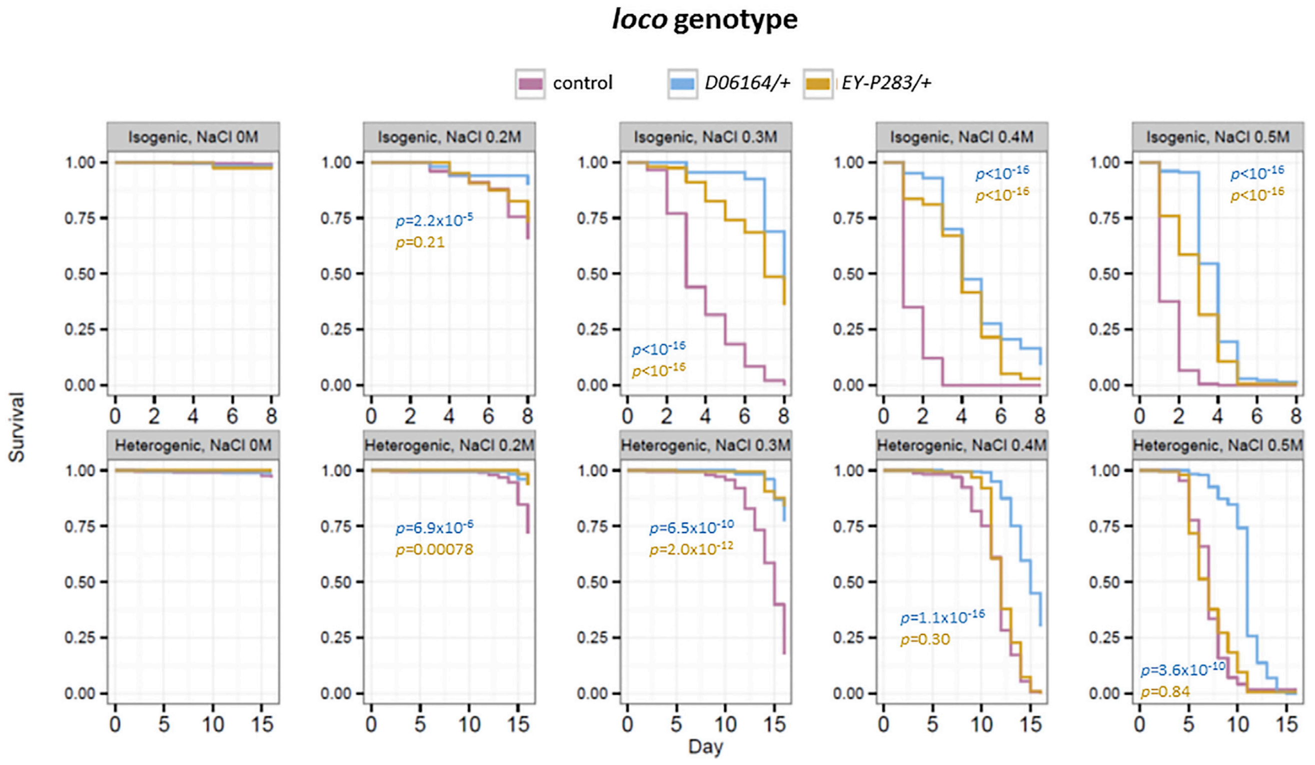

Figure 2. ***Drosophila RGS14* Heterozygous Mutants Are Resistant to Salt Stress**
Survival of flies carrying heterozygous loss-of-function mutations in the *Drosophila melanogaster RGS14* homolog, *loco*, was compared to that of controls of the same genetic background. In the isogenic experiment, all genotypes were backcrossed to the control strain. In the heterogenic experiment, controls and *loco* mutants were crossed with the A.R.R. lab's *wBerlin* strain to obtain highly heterozygous progeny. Kaplan-Meier plots demonstrated that flies heterozygous for two independently derived loco mutations, $loco^{EY-P283}$ and $loco^{d06164}$, were resistant to salt stress across a range of NaCl concentrations when compared to controls. Cox-proportional hazards p values for each mutant, compared to those of controls, are presented and are calculated for each genetic background (isogenic or heterogenic) and NaCl concentration separately. Results are based on 170–200 flies per genotype for each NaCl concentration.

for this gene in resistance to salt stress. To exclude the effects of inbreeding depression on our findings, we also repeated our experiments with the same strains on a heterogenic background. As expected, the hybrid heterogenic strains were less salt susceptible than the isogenic strains, but the *loco* mutants remained salt-resistant when compared to controls of a similar genetic background (Figure 2, Table S14).

To further investigate the role of *NFATC1* and *RGS14* in kidney function, we used the 129S6 mouse strain that is salt-sensitive[31] and susceptible to glomerulosclerosis.[40] We compared the renal mRNA expression of *Nfatc1* and *Rgs14* at baseline versus (1) after a 2-week exposure to high-salt diet and (2) at 12 weeks after CKD induced by sub-total nephrectomy. Compared to baseline condition, *Rgs14* was significantly decreased (~75%, $p = 0.01$) during high-salt exposure (Figure 3). In the CKD model, *Rgs14* expression was also reduced and approached statistical significance ($p = 0.06$). The renal mRNA expression of *Nfatc1* was also significantly decreased (~50%, $p = 0.03$) during high-salt exposure and trended down in CKD ($p = 0.31$). Although we cannot establish cause and effect, these data illustrate that the expression of both genes is altered during disease states.

Discussion

We have undertaken a trans-ethnic meta-analysis of GWASs of eGFR, supplemented by imputation up to the phase 1 integrated (March 2012 release) multi-ethnic reference panel from the 1000 Genomes Project.[9] With these high-density imputed data, we identified 20 loci at genome-wide significance for eGFR through trans-ethnic meta-analysis. Despite improved coverage of low-frequency variation offered by high-density imputation, lead SNPs were common across ancestry groups at all 20 of these kidney-function loci. There was also minimal evidence of trans-ethnic heterogeneity in allelic effects on eGFR at lead SNPs at kidney-function loci, thereby arguing against the "synthetic association" hypothesis.[41] It is highly unlikely that eGFR association signals at these kidney-function loci reflect unobserved lower frequency causal alleles with larger effects because (1) rare variants are unlikely to have arisen before human population migration out of Africa and thus are not anticipated to be widely shared across diverse populations[9,42] and (2) LD with these variants is expected to be highly variable between ethnicities.

Our conditional analyses did not provide evidence for multiple distinct eGFR association signals, which is

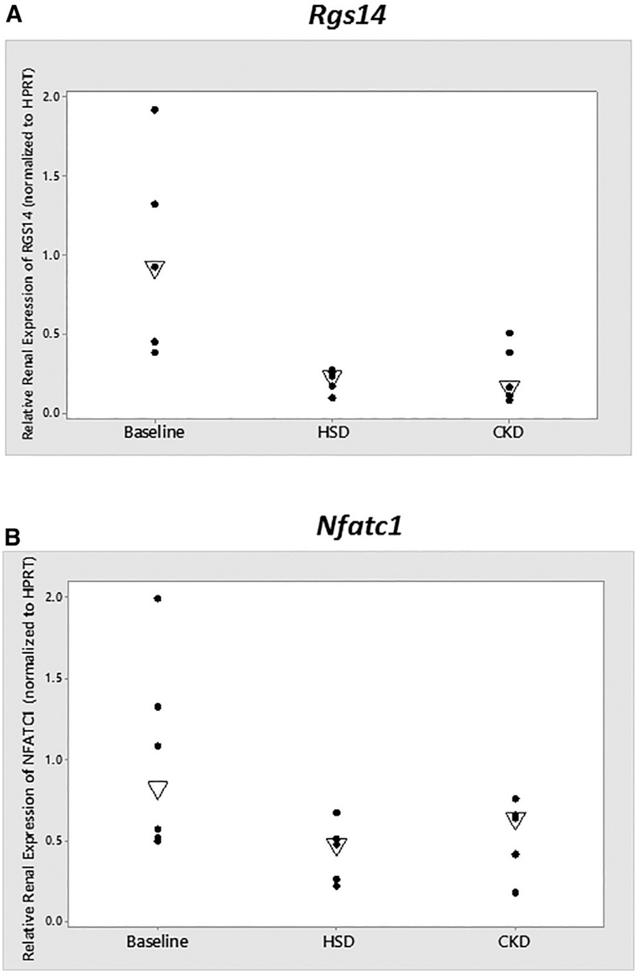

Figure 3. Relative Renal mRNA Expression of *Rgs14* and *Nfatc1*
Expression of *Rgs14* is shown in (A) and *Nfatc1* in (B). n = 5 or 6 in each group. The empty triangle represents the median. According to the Mann-Whitney test, and compared to the baseline, *Rgs14* expression after exposure to a high-salt diet was significantly lower ($p = 0.01$), and was also lower in CKD ($p = 0.06$). *Nfatc1* expression after exposure to a high-salt diet was also significantly lower ($p = 0.03$) and trended in the same direction in CKD ($p = 0.31$).

consistent with a single causal variant at each of the 20 eGFR loci. However, we recognize that conditional analyses evaluate the evidence for residual association at the locus that cannot be ascribed to the lead SNP and do not provide a formal framework to test for the presence of multiple causal variants, for example, that are in strong LD with each other and reside on the same haplotype. Furthermore, larger sample sizes will be required to detect distinct association signals defined by common variants of modest effect or low-frequency variants that might be specific to particular ethnic groups.

As with most previous GWASs of kidney function, our study was limited to a single measure of eGFR for each participant. We also did not adjust for diabetes or hypertension in our analyses given that these conditions are potential mediators or modifiers of the SNP-eGFR associations. However, despite ethnic differences in the prevalence of these conditions, we observed no evidence of heterogeneity in allelic effects on eGFR between ancestry groups. Exploration of context-dependent effects should be considered in future studies, for example, by using gene-environment interaction or mediation analyses.

Given our observation that eGFR association signals are shared across ancestry groups, we next sought to take advantage of the differential patterns of LD across diverse populations to fine-map kidney-function loci. Credible-set variants mapped predominantly to non-coding sequence, suggesting that eGFR association signals are most likely to be mediated by effects on gene regulation, in agreement with previous reports for other complex human traits.[43–45] Through integration of genetic fine-mapping data with information from regulatory annotation resources, we have demonstrated significant enrichment of variants driving eGFR association signals with DHSs in multiple kidney cell types. Overlap with these enriched annotations could be used as a prior model for eGFR association signals, genome-wide, to improve power for discovery of additional kidney-function loci and further enhance trans-ethnic fine-mapping efforts.[46]

Lead SNPs at kidney-function loci overlapping enriched annotations included eQTL for *NFATC1* and *RGS14* (at the *SLC34A1* locus) in multiple tissues, pointing to likely effector transcripts through which these eGFR association signals are mediated. We have established that loss-of-function mutations in ancestral orthologs of both genes in *Drosophila melanogaster* are associated with response to salt stress. Although salt sensitivity has not been directly correlated with variation in eGFR in humans, it has been associated with albuminuria,[47,48] elevated creatinine,[48] and the subsequent development of hypertension,[49] suggesting the relevance of this trait to kidney function. Indeed, in animal models, salt sensitivity is tightly linked with a blunted tubuloglomerular feedback (TGF) or impaired increase in GFR after salt loading.[50–53] Consistent with this, we demonstrated that renal mRNA expression of *Nfatc1* and *Rgs14* in a salt-sensitive mouse model was reduced after exposure to a high-salt diet and induced CKD. In parallel with the findings in *Drosophila melanogaster*, these results are consistent with the hypothesis that the capacity to reduce expression of *Rgs14* and *Nfatc1* determines the extent of the response to stress. Another possible mechanism suggested by our results in *Drosophila* is a role for oxidative stress, to which *RGS14* ortholog mutants are resistant,[30] and which has been implicated in mammalian salt sensitivity.[54,55] Establishing the functional role of these genes in salt sensitivity, TGF, GFR, oxidative stress, and CKD will require targeted in vivo studies using knockout and/or transgenic mouse models.

In conclusion, our study demonstrates the utility of trans-ethnic fine mapping through integration of GWASs of diverse populations with genomic annotation from relevant tissues to define molecular mechanisms by which association signals exert their effect, thereby offering an exciting opportunity to elucidate the pathophysiology of complex human diseases.

Supplemental Data

Supplemental Data include five figures, fourteen tables, and Supplemental Acknowledgments and can be found with this article online at http://dx.doi.org/10.1016/j.ajhg.2016.07.012.

Acknowledgments

A.R.R. and J.N.S. are supported by the US NIH (K08DK091316). T.H.L. is supported by the National Institute of Diabetes and Digestive and Kidney Diseases (1R01DK094907-01). S.P. is supported by the US NIH (R25DK101401). G.W.M. is supported by the Australian National Health and Medical Research Council Fellowship Scheme. Yukinori Okada was supported by the Japan Society for the Promotion of Science KAKENHI (15H05911, 15H05670, 15K14429), the Japan Science and Technology Agency, Mochida Memorial Foundation for Medical and Pharmaceutical Research, Takeda Science Foundation, Gout Research Foundation, the Tokyo Biochemical Research Foundation, and the Japan Rheumatism Foundation. A.P.M. is a Wellcome Trust Senior Fellow in Basic Biomedical Science (grant WT098017). N.F. is supported by the US NIH (5R21HL123677-02). The ESRD data reported here have been supplied by the United States Renal Data System. The interpretation and reporting of these data are the responsibility of the authors and in no way should be seen as official policy or interpretation of the US government. Additional acknowledgments are provided in the Supplemental Data.

Received: February 17, 2016
Accepted: July 8, 2016
Published: September 1, 2016

Web Resources

1000 Genomes, http://www.1000genomes.org
ENCODE, https://www.encodeproject.org/
Ensembl Genome Browser, http://www.ensembl.org/index.html
EPACTS, http://genome.sph.umich.edu/wiki/EPACTS
fGWAS, https://github.com/joepickrell/fgwas
Gencode, http://www.gencodegenes.org
GWAMA, http://www.geenivaramu.ee/en/tools/gwama
IMPUTE2, http://mathgen.stats.ox.ac.uk/impute/impute_v2.html
International HapMap Project, http://hapmap.ncbi.nlm.nih.gov/
LDlink, http://analysistools.nci.nih.gov/LDlink/
METAL, http://www.sph.umich.edu/csg/abecasis/metal/
Minimac, http://genome.sph.umich.edu/wiki/Minimac
OMIM, http://www.omim.org/
Roadmap, http://www.roadmapepigenomics.org/
Variant Effect Predictor, http://useast.ensembl.org/Homo_sapiens/Tools/VEP

References

1. Levey, A.S., and Coresh, J. (2012). Chronic kidney disease. Lancet *379*, 165–180.
2. Collins, A.J., Foley, R.N., Gilbertson, D.T., and Chen, S.-C. (2015). United States Renal Data System public health surveillance of chronic kidney disease and end-stage renal disease. Kidney Int Suppl (2011) *5*, 2–7.
3. Xue, J.L., Eggers, P.W., Agodoa, L.Y., Foley, R.N., and Collins, A.J. (2007). Longitudinal study of racial and ethnic differences in developing end-stage renal disease among aged medicare beneficiaries. J. Am. Soc. Nephrol. *18*, 1299–1306.
4. Köttgen, A., Pattaro, C., Böger, C.A., Fuchsberger, C., Olden, M., Glazer, N.L., Parsa, A., Gao, X., Yang, Q., Smith, A.V., et al. (2010). New loci associated with kidney function and chronic kidney disease. Nat. Genet. *42*, 376–384.
5. Pattaro, C., Köttgen, A., Teumer, A., Garnaas, M., Böger, C.A., Fuchsberger, C., Olden, M., Chen, M.-H., Tin, A., Taliun, D., et al.; CARDIoGRAM Consortium; ICBP Consortium; CARe Consortium; Wellcome Trust Case Control Consortium 2 (WTCCC2) (2012). Genome-wide association and functional follow-up reveals new loci for kidney function. PLoS Genet. *8*, e1002584.
6. Okada, Y., Sim, X., Go, M.J., Wu, J.Y., Gu, D., Takeuchi, F., Takahashi, A., Maeda, S., Tsunoda, T., Chen, P., et al.; KidneyGen Consortium; CKDGen Consortium; GUGC consortium (2012). Meta-analysis identifies multiple loci associated with kidney function-related traits in east Asian populations. Nat. Genet. *44*, 904–909.
7. Gorski, M., Tin, A., Garnaas, M., McMahon, G.M., Chu, A.Y., Tayo, B.O., Pattaro, C., Teumer, A., Chasman, D.I., Chalmers, J., et al. (2015). Genome-wide association study of kidney function decline in individuals of European descent. Kidney Int. *87*, 1017–1029.
8. Pattaro, C., Teumer, A., Gorski, M., Chu, A.Y., Li, M., Mijatovic, V., Garnaas, M., Tin, A., Sorice, R., Li, Y., et al.; ICBP Consortium; AGEN Consortium; CARDIOGRAM; CHARGe-Heart Failure Group; ECHOGen Consortium (2016). Genetic associations at 53 loci highlight cell types and biological pathways relevant for kidney function. Nat. Commun. *7*, 10023.
9. Abecasis, G.R., Auton, A., Brooks, L.D., DePristo, M.A., Durbin, R.M., Handsaker, R.E., Kang, H.M., Marth, G.T., and McVean, G.A.; The 1000 Genomes Project Consortium (2012). An integrated map of genetic variation from 1,092 human genomes. Nature *491*, 56–65.
10. Delaneau, O., Marchini, J., and Zagury, J.F. (2011). A linear complexity phasing method for thousands of genomes. Nat. Methods *9*, 179–181.
11. Howie, B., Fuchsberger, C., Stephens, M., Marchini, J., and Abecasis, G.R. (2012). Fast and accurate genotype imputation in genome-wide association studies through pre-phasing. Nat. Genet. *44*, 955–959.
12. Frazer, K.A., Ballinger, D.G., Cox, D.R., Hinds, D.A., Stuve, L.L., Gibbs, R.A., Belmont, J.W., Boudreau, A., Hardenbol, P., Leal, S.M., et al.; International HapMap Consortium (2007). A second generation human haplotype map of over 3.1 million SNPs. Nature *449*, 851–861.
13. Howie, B.N., Donnelly, P., and Marchini, J. (2009). A flexible and accurate genotype imputation method for the next generation of genome-wide association studies. PLoS Genet. *5*, e1000529.
14. Winkler, T.W., Day, F.R., Croteau-Chonka, D.C., Wood, A.R., Locke, A.E., Mägi, R., Ferreira, T., Fall, T., Graff, M., Justice, A.E., et al.; Genetic Investigation of Anthropometric Traits (GIANT) Consortium (2014). Quality control and conduct of genome-wide association meta-analyses. Nat. Protoc. *9*, 1192–1212.
15. National Kidney Foundation (2002). K/DOQI clinical practice guidelines for chronic kidney disease: evaluation, classification, and stratification. Am. J. Kidney Dis. *39* (2 Suppl 1), S1–S266.
16. Devlin, B., and Roeder, K. (1999). Genomic control for association studies. Biometrics *55*, 997–1004.

17. Mägi, R., and Morris, A.P. (2010). GWAMA: software for genome-wide association meta-analysis. BMC Bioinformatics *11*, 288.
18. Ioannidis, J.P.A., Patsopoulos, N.A., and Evangelou, E. (2007). Heterogeneity in meta-analyses of genome-wide association investigations. PLoS ONE *2*, e841.
19. Machiela, M.J., and Chanock, S.J. (2015). LDlink: a web-based application for exploring population-specific haplotype structure and linking correlated alleles of possible functional variants. Bioinformatics *31*, 3555–3557.
20. Willer, C.J., Li, Y., and Abecasis, G.R. (2010). METAL: fast and efficient meta-analysis of genomewide association scans. Bioinformatics *26*, 2190–2191.
21. Kang, H.M., Sul, J.H., Service, S.K., Zaitlen, N.A., Kong, S.Y., Freimer, N.B., Sabatti, C., and Eskin, E. (2010). Variance component model to account for sample structure in genome-wide association studies. Nat. Genet. *42*, 348–354.
22. Morris, A.P. (2011). Transethnic meta-analysis of genomewide association studies. Genet. Epidemiol. *35*, 809–822.
23. Franceschini, N., van Rooij, F.J.A., Prins, B.P., Feitosa, M.F., Karakas, M., Eckfeldt, J.H., Folsom, A.R., Kopp, J., Vaez, A., Andrews, J.S., et al.; LifeLines Cohort Study (2012). Discovery and fine mapping of serum protein loci through transethnic meta-analysis. Am. J. Hum. Genet. *91*, 744–753.
24. Wang, X., Chua, H.X., Chen, P., Ong, R.T., Sim, X., Zhang, W., Takeuchi, F., Liu, X., Khor, C.C., Tay, W.T., et al. (2013). Comparing methods for performing trans-ethnic meta-analysis of genome-wide association studies. Hum. Mol. Genet. *22*, 2303–2311.
25. Maller, J.B., McVean, G., Byrnes, J., Vukcevic, D., Palin, K., Su, Z., Howson, J.M., Auton, A., Myers, S., Morris, A., et al.; Wellcome Trust Case Control Consortium (2012). Bayesian refinement of association signals for 14 loci in 3 common diseases. Nat. Genet. *44*, 1294–1301.
26. Bernstein, B.E., Stamatoyannopoulos, J.A., Costello, J.F., Ren, B., Milosavljevic, A., Meissner, A., Kellis, M., Marra, M.A., Beaudet, A.L., Ecker, J.R., et al. (2010). The NIH Roadmap Epigenomics Mapping Consortium. Nat. Biotechnol. *28*, 1045–1048.
27. ENCODE Project Consortium (2012). An integrated encyclopedia of DNA elements in the human genome. Nature *489*, 57–74.
28. Pasquali, L., Gaulton, K.J., Rodríguez-Seguí, S.A., Mularoni, L., Miguel-Escalada, I., Akerman, I., Tena, J.J., Morán, I., Gómez-Marín, C., van de Bunt, M., et al. (2014). Pancreatic islet enhancer clusters enriched in type 2 diabetes risk-associated variants. Nat. Genet. *46*, 136–143.
29. Pickrell, J.K. (2014). Joint analysis of functional genomic data and genome-wide association studies of 18 human traits. Am. J. Hum. Genet. *94*, 559–573.
30. Lin, Y.R., Kim, K., Yang, Y., Ivessa, A., Sadoshima, J., and Park, Y. (2011). Regulation of longevity by regulator of G-protein signaling protein, Loco. Aging Cell *10*, 438–447.
31. Cechova, S., Zeng, Q., Billaud, M., Mutchler, S., Rudy, C.K., Straub, A.C., Chi, L., Chan, F.R., Hu, J., Griffiths, R., et al. (2013). Loss of collectrin, an angiotensin-converting enzyme 2 homolog, uncouples endothelial nitric oxide synthase and causes hypertension and vascular dysfunction. Circulation *128*, 1770–1780.
32. Yang, Y., Parsons, K.K., Chi, L., Malakauskas, S.M., and Le, T.H. (2009). Glutathione S-transferase-micro1 regulates vascular smooth muscle cell proliferation, migration, and oxidative stress. Hypertension *54*, 1360–1368.
33. Deshmukh, H.A., Palmer, C.N., Morris, A.D., and Colhoun, H.M. (2013). Investigation of known estimated glomerular filtration rate loci in patients with type 2 diabetes. Diabet. Med. *30*, 1230–1235.
34. Sveinbjornsson, G., Mikaelsdottir, E., Palsson, R., Indridason, O.S., Holm, H., Jonasdottir, A., Helgason, A., Sigurdsson, S., Jonasdottir, A., Sigurdsson, A., et al. (2014). Rare mutations associating with serum creatinine and chronic kidney disease. Hum. Mol. Genet. *23*, 6935–6943.
35. Succurro, E., Arturi, F., Lugarà, M., Grembiale, A., Fiorentino, T.V., Caruso, V., Andreozzi, F., Sciacqua, A., Hribal, M.L., Perticone, F., and Sesti, G. (2010). One-hour postload plasma glucose levels are associated with kidney dysfunction. Clin. J. Am. Soc. Nephrol. *5*, 1922–1927.
36. Reiss, A.B., Voloshyna, I., De Leon, J., Miyawaki, N., and Mattana, J. (2015). Cholesterol Metabolism in CKD. Am. J. Kidney Dis. *66*, 1071–1082.
37. Chen, S., Bellew, C., Yao, X., Stefkova, J., Dipp, S., Saifudeen, Z., Bachvarov, D., and El-Dahr, S.S. (2011). Histone deacetylase (HDAC) activity is critical for embryonic kidney gene expression, growth, and differentiation. J. Biol. Chem. *286*, 32775–32789.
38. Brilli, L.L., Swanhart, L.M., de Caestecker, M.P., and Hukriede, N.A. (2013). HDAC inhibitors in kidney development and disease. Pediatr. Nephrol. *28*, 1909–1921.
39. Keyser, P., Borge-Renberg, K., and Hultmark, D. (2007). The Drosophila NFAT homolog is involved in salt stress tolerance. Insect Biochem. Mol. Biol. *37*, 356–362.
40. Salzler, H.R., Griffiths, R., Ruiz, P., Chi, L., Frey, C., Marchuk, D.A., Rockman, H.A., and Le, T.H. (2007). Hypertension and albuminuria in chronic kidney disease mapped to a mouse chromosome 11 locus. Kidney Int. *72*, 1226–1232.
41. Dickson, S.P., Wang, K., Krantz, I., Hakonarson, H., and Goldstein, D.B. (2010). Rare variants create synthetic genome-wide associations. PLoS Biol. *8*, e1000294.
42. Abecasis, G.R., Altshuler, D., Auton, A., Brooks, L.D., Durbin, R.M., Gibbs, R.A., Hurles, M.E., and McVean, G.A.; The 1000 Genomes Project Consortium (2010). A map of human genome variation from population scale sequencing. Nature *467*, 1061–1073.
43. Trynka, G., Sandor, C., Han, B., Xu, H., Stranger, B.E., Liu, X.S., and Raychaudhuri, S. (2013). Chromatin marks identify critical cell types for fine mapping complex trait variants. Nat. Genet. *45*, 124–130.
44. Parker, S.C., Stitzel, M.L., Taylor, D.L., Orozco, J.M., Erdos, M.R., Akiyama, J.A., van Bueren, K.L., Chines, P.S., Narisu, N., Black, B.L., et al.; NISC Comparative Sequencing Program; National Institutes of Health Intramural Sequencing Center Comparative Sequencing Program Authors; NISC Comparative Sequencing Program Authors (2013). Chromatin stretch enhancer states drive cell-specific gene regulation and harbor human disease risk variants. Proc. Natl. Acad. Sci. USA *110*, 17921–17926.
45. Gusev, A., Lee, S.H., Trynka, G., Finucane, H., Vilhjálmsson, B.J., Xu, H., Zang, C., Ripke, S., Bulik-Sullivan, B., Stahl, E., et al.; Schizophrenia Working Group of the Psychiatric Genomics Consortium; SWE-SCZ Consortium; Schizophrenia Working Group of the Psychiatric Genomics Consortium; SWE-SCZ Consortium (2014). Partitioning heritability of regulatory and cell-type-specific variants across 11 common diseases. Am. J. Hum. Genet. *95*, 535–552.

46. Kichaev, G., and Pasaniuc, B. (2015). Leveraging functional-annotation data in trans-ethnic fine-mapping studies. Am. J. Hum. Genet. *97*, 260–271.
47. Bigazzi, R., Bianchi, S., Baldari, D., Sgherri, G., Baldari, G., and Campese, V.M. (1994). Microalbuminuria in salt-sensitive patients. A marker for renal and cardiovascular risk factors. Hypertension *23*, 195–199.
48. Bihorac, A., Tezcan, H., Ozener, C., Oktay, A., and Akoglu, E. (2000). Association between salt sensitivity and target organ damage in essential hypertension. Am. J. Hypertens. *13*, 864–872.
49. Barba, G., Galletti, F., Cappuccio, F.P., Siani, A., Venezia, A., Versiero, M., Della Valle, E., Sorrentino, P., Tarantino, G., Farinaro, E., and Strazzullo, P. (2007). Incidence of hypertension in individuals with different blood pressure salt-sensitivity: results of a 15-year follow-up study. J. Hypertens. *25*, 1465–1471.
50. Thurau, K., and Schnermann, J. (1965). The sodium concentration in the macula densa cells as a regulating factor for glomerular filtration (micropuncture experiments). Klin. Wochenschr. *43*, 410–413.
51. Wilcox, C.S., and Welch, W.J. (1996). TGF and nitric oxide: effects of salt intake and salt-sensitive hypertension. Kidney Int. Suppl. *55*, S9–S13.
52. Persson, A.E.G., Lai, E.Y., Gao, X., Carlström, M., and Patzak, A. (2013). Interactions between adenosine, angiotensin II and nitric oxide on the afferent arteriole influence sensitivity of the tubuloglomerular feedback. Front. Physiol. *4*, 187.
53. Lu, Y., Wei, J., Stec, D.E., Roman, R.J., Ge, Y., Cheng, L., Liu, E.Y., Zhang, J., Hansen, P.B., Fan, F., et al. (2016). Macula Densa Nitric Oxide Synthase 1β Protects against Salt-Sensitive Hypertension. J. Am. Soc. Nephrol. *27*, 2346–2356.
54. Kanbay, M., Chen, Y., Solak, Y., and Sanders, P.W. (2011). Mechanisms and consequences of salt sensitivity and dietary salt intake. Curr. Opin. Nephrol. Hypertens. *20*, 37–43.
55. Morris, R.C., Jr., Schmidlin, O., Sebastian, A., Tanaka, M., and Kurtz, T.W. (2016). Vasodysfunction that involves renal vasodysfunction, not abnormally increased renal retention of sodium, accounts for the initiation of salt-induced hypertension. Circulation *133*, 881–893.

The American Journal of Human Genetics, Volume *99*

Supplemental Data

Trans-ethnic Fine Mapping Highlights Kidney-Function Genes Linked to Salt Sensitivity

Anubha Mahajan, Aylin R. Rodan, Thu H. Le, Kyle J. Gaulton, Jeffrey Haessler, Adrienne M. Stilp, Yoichiro Kamatani, Gu Zhu, Tamar Sofer, Sanjana Puri, Jeffrey N. Schellinger, Pei-Lun Chu, Sylvia Cechova, Natalie van Zuydam, the SUMMIT Consortium, the BioBank Japan Project, Johan Arnlov, Michael F. Flessner, Vilmantas Giedraitis, Andrew C. Heath, Michiaki Kubo, Anders Larsson, Cecilia M. Lindgren, Pamela A.F. Madden, Grant W. Montgomery, George J. Papanicolaou, Alex P. Reiner, Johan Sundström, Timothy A. Thornton, Lars Lind, Erik Ingelsson, Jianwen Cai, Nicholas G. Martin, Charles Kooperberg, Koichi Matsuda, John B. Whitfield, Yukinori Okada, Cathy C. Laurie, Andrew P. Morris, and Nora Franceschini

Figure S1. Dendogram to summarise relatedness between studies. The dendogram was constructed on the basis of genome-wide pair-wise allele frequency differences between studies. European ancestry studies are grouped in the red cluster, and Hispanic ancestry studies are grouped in the blue cluster.

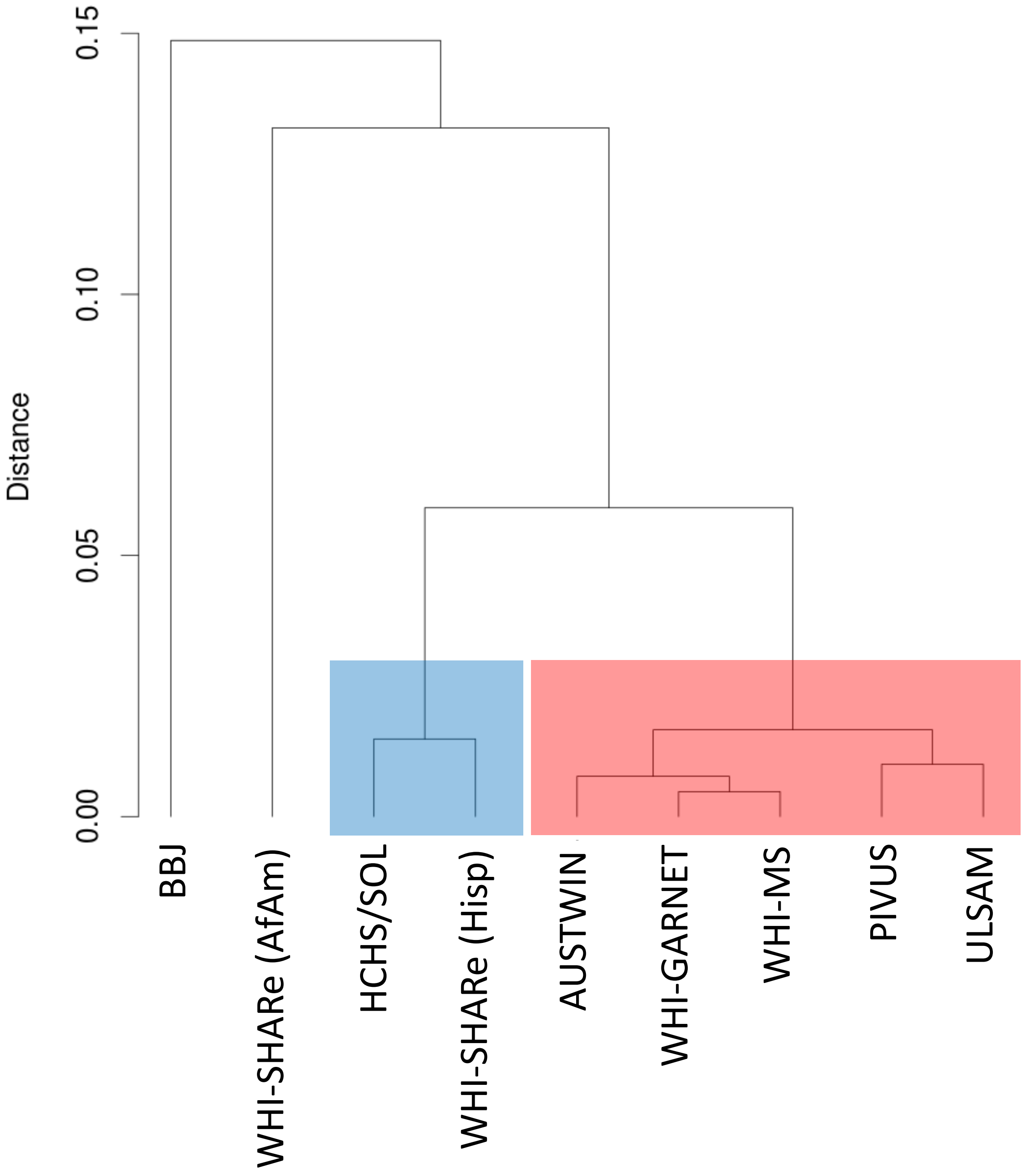

Figure S2. Genome-wide eGFR association summary from the trans-ethnic meta-analysis of 71,638 individuals. Each point corresponds to a SNP passing quality control in the meta-analysis, plotted according to physical position (NCBI build 37) on the x-axis and $-\log_{10}$ *p*-value on the y-axis. The locus names of loci attaining genome-wide significance ($p<5\times10^{-8}$, horizontal red line) are highlighted above the Manhattan plot. Association signals mapping to previously established loci are highlighted in green.

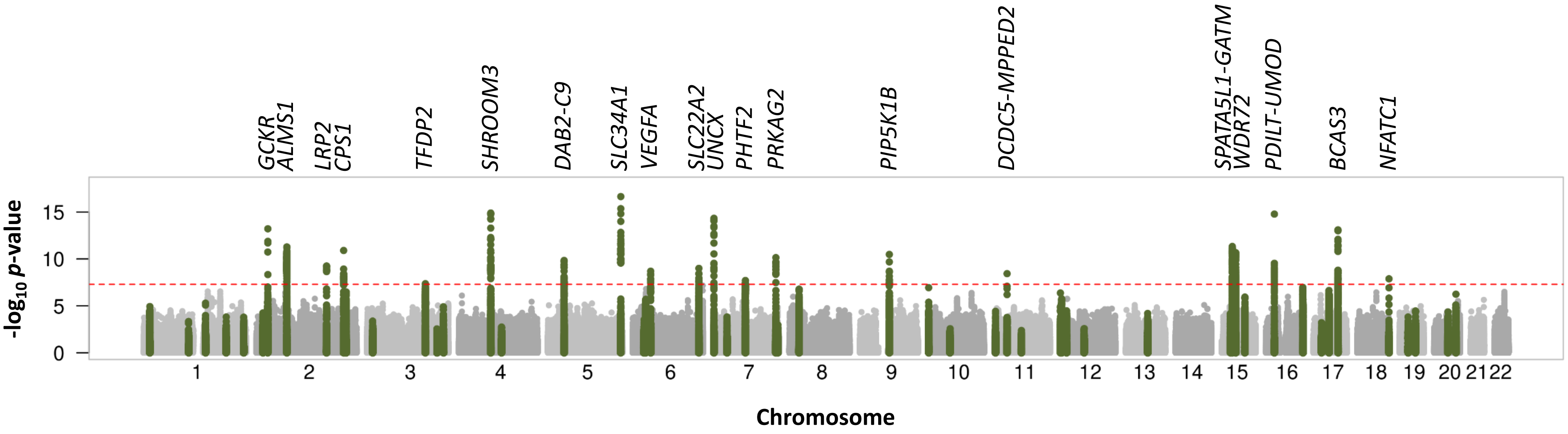

Figure S3. Comparison of allelic effects (beta) of lead SNPs on eGFR in the general population (from our trans-ethnic meta-analysis of 71,638 individuals) and in diabetics (from a meta-analysis of 13,158 individuals from the SUMMIT Consortium). Grey bars represent 95% confidence intervals for allelic effect sizes. The lead SNP at the *PDILT-UMOD* locus demonstrates greater allelic effect on eGFR in diabetics than in the general population at nominal significance ($p<0.05$).

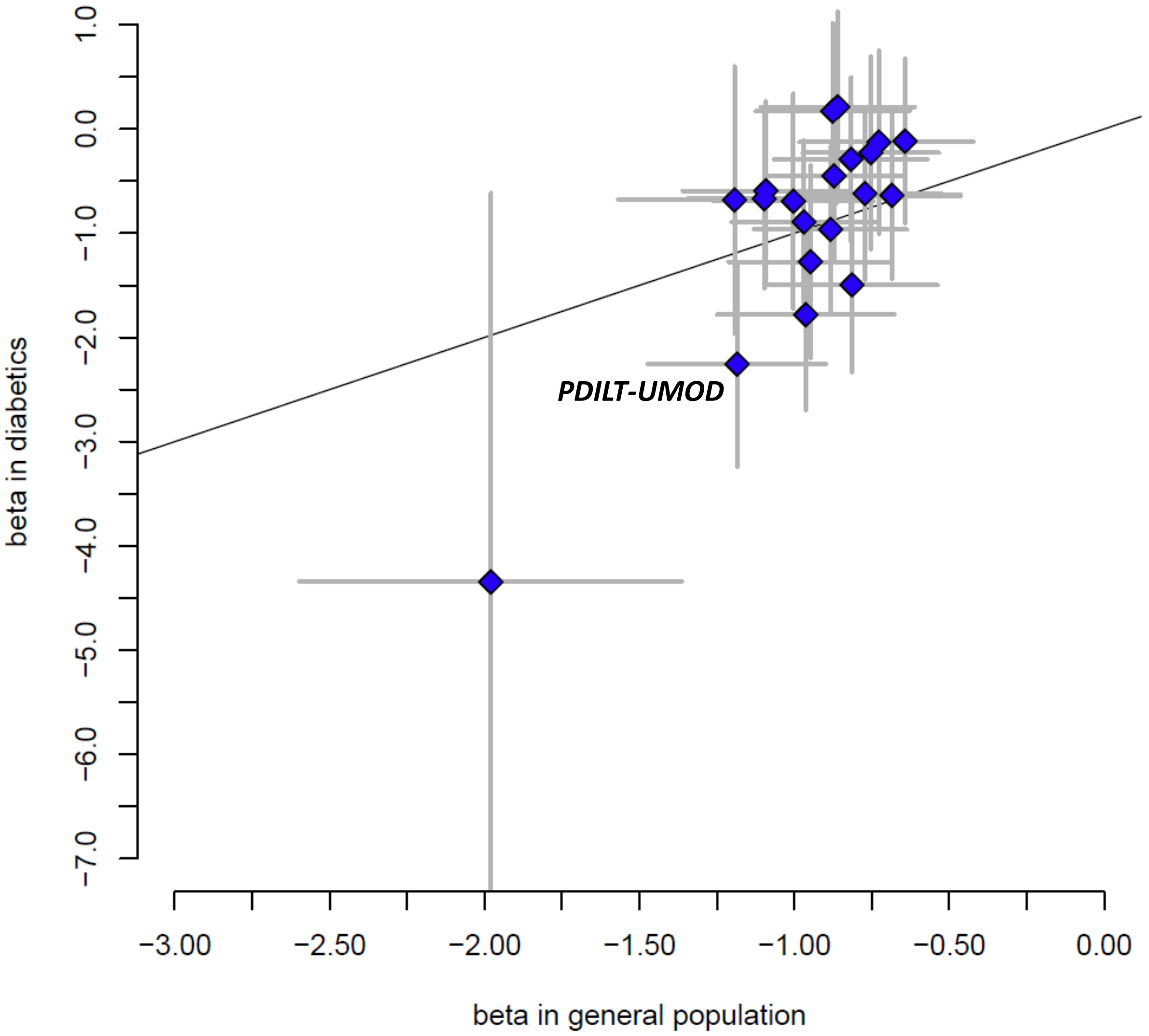

Figure S4. Enrichment of genomic annotations of regulatory chromatin state for 93 cell types, DHS for 145 cell types, and chromatin immuno-precipitation sequence binding sites for 165 transcription factors for Bayes' factors in favour of eGFR association. Each point corresponds to an annotation, plotted according to the effect size (log-enrichment in Bayes' factor) on the x-axis, and ranked according to the significance of the association on the y-axis. Significant enrichments are highlighted in red.

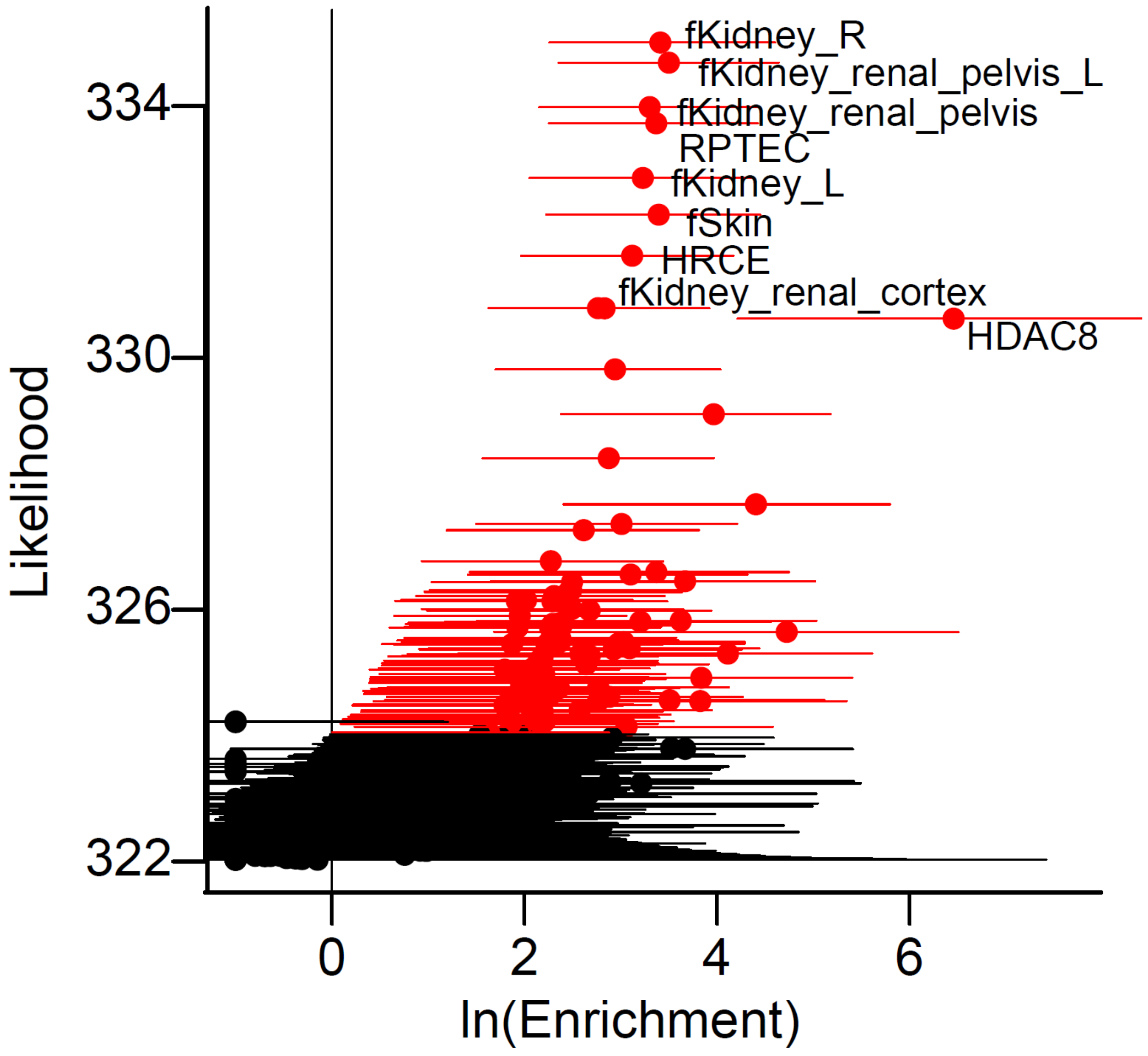

Figure S5. Overlap of credible set variants with enriched regulatory annotations at the *SLC34A1* and *NFATC1* loci. Each point corresponds to a SNP, plotted according to their chromosomal position and posterior probability of driving the eGFR association signal. The locations of enriched regulatory annotations (DHS in multiple kidney cell-types and TFBS for HDAC8) are highlighted for each locus.

SLC34A1 locus

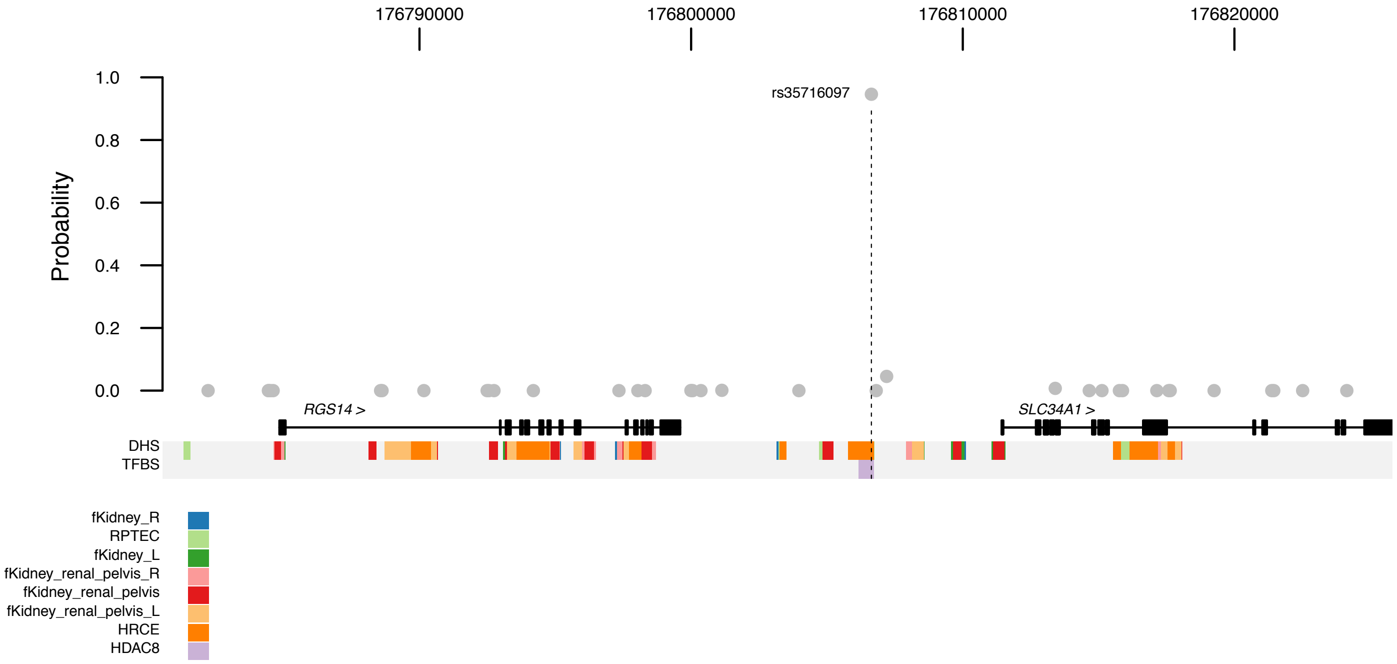

NFATC1 locus

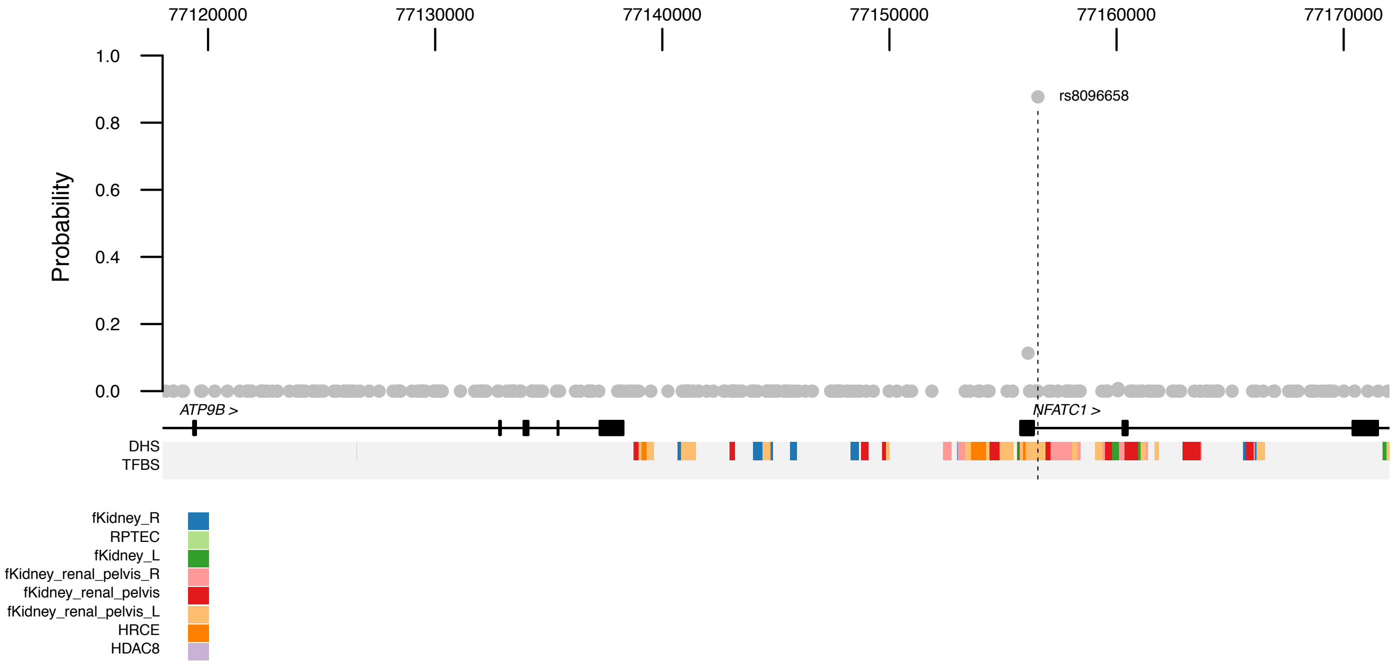

Table S1. Study sample characteristics.

Study (acronym)	Ethnicity (origin)	Sex	Sample characteristics				
			Sample size	Age (years) mean (SD)	Serum Creatinine (mg/dL) mean (SD)	eGFR mean (SD)	CKD cases/controls
Prospective Investigation of the Vasculature in Uppsala Seniors (PIVUS)	European (Sweden)	Males	471	70.1 (0.1)	0.99 (0.22)	83.8 (19.9)	136/808
		Females	473	70.2 (0.2)	0.82 (0.18)	77.9 (20.2)	
Uppsala Longitudinal Study of Adult Men (ULSAM)	European (Sweden)	Males	1,080	71.0 (0.6)	1.06 (0.15)	75.2 (11.3)	88/992
		Females	0	N/A	N/A	N/A	
Australian Twin-Family Studies (AUSTWIN)	European (Australia)	Males	4,662	48.7 (13.1)	1.13 (0.20)	76.6 (15.8)	NA/NA
		Females	7,096	46.9 (13.4)	0.90 (0.16)	75.1 (16.5)	
Women's Health Initiative Memory Study (WHI-MS)	European (USA)	Males	0	N/A	N/A	N/A	343/5,312
		Females	5,655	68.1 (5.9)	0.75 (0.15)	85.6 (17.8)	
Women's Health Initiative Genome-wide Association Research Network into Effects of Treatment (WHI-GARNET)	European (USA)	Males	0	N/A	N/A	N/A	240/3,876
		Females	4,116	65.6 (6.9)	0.74 (0.15)	88.1 (19.3)	
BioBank Japan Project (BBJ)	East Asian (Japan)	Males	12,802	64.4 (9.8)	0.89 (0.29)	100.2 (28.5)	1,330/22,206
		Females	10,734	60.7 (13.1)	0.64 (0.20)	109.1 (31.0)	
Hispanic Community Health Study and Study of Latinos (HCHS/SOL)	Hispanic (USA)	Males	5,242	45.3 (14.2)	0.98 (0.44)	95.5 (22.3)	462/12,314
		Females	7,534	46.7 (13.6)	0.73 (0.23)	96.6 (23.4)	
Women's Health Initiative SNP Health Association Resource (WHI-SHARe)	Hispanic (USA)	Males	0	N/A	N/A	N/A	174/3,375
		Females	3,549	60.3 (6.7)	0.71 (0.19)	94.7 (21.9)	
	African American (USA)	Males	0	N/A	N/A	N/A	1,203/7,021
		Females	8,224	61.6 (7.0)	0.82 (0.22)	80.1 (19.4)	

SD: standard deviation.

Table S2. Summary of study-specific genotyping, quality control, imputation and analysis.

Study acronym	Genotyping array	Sample quality control		Scaffold quality control			Pre-phasing and imputation			Association analysis		
		Call rate	Exclusions	Call rate	HWE *p*	MAF	Software	Quality filter	Passed SNPs	Software	Covariates	λ_{GC}
PIVUS	Illumina OmniExpress & Metabochip	95%	Heterozygosity, gender check and relatedness	95% (99% if MAF<5%)	10^{-6}	1%	SHAPEITv2 IMPUTEv2	info≥0.4	9,316,737	SNPTESTv2	Age, sex, 2 PCs	0.982
ULSAM	Illumina Omni2.5M & Metabochip	95%	Heterozygosity, gender check and relatedness	95% (99% if MAF<5%)	10^{-6}	1%	SHAPEITv2 IMPUTEv2	info≥0.4	9,388,420	SNPTESTv2	Age, 2 PCs	1.013
AUSTWIN	Illumina 317K, 370K, 610K, OmniExpress, Omni2.5 & HumanCoreExome	95%	Heterozygosity, gender check and relatedness	95%	10^{-6}	1%	MaCH minimac	r^2≥0.3	8,584,822	MERLIN	Age, sex, sub-study, 10 PCs	1.120
WHI-MS	Illumina OmniExpress-Exome	None	Ethnic outliers, gender check, relatedness and duplicates	97%	10^{-4}	1%	Beagle minimac	r^2≥0.3	8,814,333	ProbAbel/R	Age, centre, 10 PCs	1.025
WHI-GARNET	Illumina Human Omni1-Quad	None	Ethnic outliers, gender check, relatedness and duplicates	98%	10^{-4}	None	Beagle minimac	r^2≥0.3	8,864,693	ProbAbel/R	Age, centre, 10 PCs	1.018
BBJ	Iluumina HumanHap 610-Quad	98%	Ethnic outliers and relatedness	99%	10^{-7}	1%	MaCH minimac	r^2≥0.5	6,581,000	mach2qtl	None	1.058
HCHS/SOL	Illumina Omni2.5M & custom	98%	Gender check and duplicates	98%	10^{-5}	None	SHAPEITv2 IMPUTEv2	info≥0.4	11,374,299	LMM-OPS[a]	Age, sex, centre, sampling weights, 5PCs	1.006
WHI-SHARe (Hispanic)	Affymetrix 6.0	95%	Ethnic outliers, gender check, relatedness and duplicates	95%	10^{-6}	1%	MaCH	r^2≥0.3	10,025,812	ProbAbel	Age, centre, 10 PCs	1.027
WHI-SHARe (African American)	Affymetrix 6.0	95%	Ethnic outliers, gender check, relatedness and duplicates	95%	10^{-6}	1%	MaCH	r^2≥0.3	15,345,552	ProbAbel	Age, centre, 10 PCs	1.033

HWE: Hardy-Weinberg equilibrium. MAF: minor allele frequency. PC: principal component.
[a]In-house software, not yet publicly available; accounts for relatedness in linear mixed model.

Table S3. Real-time RT-PCR, oligonucleotide primers.

Gene	Primer Sequence
Rgs14	Forward: 5’-TGAGCCCAGTGAACATCGAC -3’<br>Reverse: 5’- TGTGCTCGGAACATATCTGGC-3'
Nfatc1	Forward: 5’-TGCCTTTTGCGAGCAGTATCT-3’<br>Reverse: 5’-CAGGCAAGGATGGGCTCATAT-3’

Table S4. Association summary statistics for eGFR at previously reported lead SNPs in established loci in trans-ethnic meta-analysis of 71,638 individuals.

Locus	SNP	Chr	Position	Alleles		Association summary statistics				Reference
			(bp, b37)	Effect[a]	Other	Beta	SE	*p*-value	*N*	
CASP9	rs12124078	1	15,869,899	G	A	-0.437	0.115	0.00019	71,636	Pattaro *et al*. (2012)[5]
SYPL2	rs12136063	1	110,014,170	G	A	-0.172	0.148	0.25	61,867	Pattaro *et al*. (2016)[8]
LASS2	rs267734	1	150,951,477	T	C	-0.311	0.158	0.052	71,638	Kottgen *et al*. (2010)[4]
CACNA1S	rs3850625	1	201,016,296	G	A	-0.795	0.207	0.00016	71,638	Pattaro *et al*. (2016)[8]
SDCCAG8	rs2802729	1	243,501,763	A	C	-0.323	0.118	0.0068	71,638	Pattaro *et al*. (2016)[8]
DDX1	rs6431731	2	15,863,002	T	C	-0.456	0.322	0.16	48,102	Pattaro *et al*. (2012)[5]
GCKR	rs1260326	2	27,730,940	C	T	-0.872	0.114	$6.1x10^{-14}$	71,638	Kottgen *et al*. (2010)[4]
ALMS1	rs13538	2	73,868,328	A	G	-0.920	0.140	$9.2x10^{-11}$	48,102	Kottgen *et al*. (2010)[4]
LRP2	rs4667594	2	170,008,506	A	T	-0.263	0.115	0.025	71,637	Pattaro *et al*. (2016)[8]
CPS1	rs7422339	2	211,540,507	A	C	-0.771	0.125	$1.2x10^{-9}$	71,638	Kottgen *et al*. (2010)[4]
IGFBP5	rs2712184	2	217,682,779	A	C	-0.573	0.114	$7.6x10^{-7}$	65,983	Pattaro *et al*. (2016)[8]
WNT7A	rs6795744	3	13,906,850	G	A	-0.159	0.156	0.32	71,638	Pattaro *et al*. (2016)[8]
TFDP2	rs347685	3	141,807,137	A	C	-0.637	0.123	$3.0x10^{-7}$	71,638	Kottgen *et al*. (2010)[4]
SKIL	rs9682041	3	170,091,902	T	C	-0.141	0.159	0.38	71,638	Pattaro *et al*. (2016)[8]
ETV5	rs10513801	3	185,822,353	G	T	-0.341	0.194	0.083	71,638	Pattaro *et al*. (2016)[8]
SHROOM3	rs17319721	4	77,368,847	A	G	-0.815	0.120	$2.2x10^{-11}$	71,638	Kottgen *et al*. (2010)[4]
NFKB1	rs228611	4	103,561,709	A	G	-0.351	0.124	0.0052	48,101	Pattaro *et al*. (2016)[8]
DAB2-C9	rs11959928	5	39,397,132	A	T	-0.719	0.113	$4.1x10^{-10}$	71,638	Kottgen *et al*. (2010)[4]
SLC34A1	rs6420094	5	176,817,636	G	A	-0.804	0.123	$1.1x10^{-10}$	71,638	Kottgen *et al*. (2010)[4]
ZNF204	rs7759001	6	27,341,409	A	G	-0.233	0.138	0.099	61,867	Pattaro *et al*. (2016)[8]
MHC region	rs3828890	6	31,440,669	C	G	-0.089	0.194	0.65	59,865	Okada *et al*. (2012)[6]
LOC100132354-VEGFA	rs881858	6	43,806,609	A	G	-0.772	0.127	$2.0x10^{-9}$	71,638	Kottgen *et al*. (2010)[4]
SLC22A2	rs2279463	6	160,668,389	G	A	-0.905	0.169	$1.4x10^{-7}$	71,638	Kottgen *et al*. (2010)[4]
UNCX	rs10277115	7	1,285,195	T	A	-1.089	0.141	$3.3x10^{-14}$	59,865	Okada *et al*. (2012)[6]
KBTBD2	rs3750082	7	32,919,927	T	A	-0.441	0.118	0.00025	71,638	Pattaro *et al*. (2016)[8]
PHTF2	rs6465825	7	77,416,439	C	T	-0.590	0.125	$3.4x10^{-6}$	61,867	Kottgen *et al*. (2010)[4]
PRKAG2	rs7805747	7	151,407,801	A	G	-0.813	0.136	$4.4x10^{-9}$	48,102	Kottgen *et al*. (2010)[4]
RNF32	rs6459680	7	156,258,568	T	G	-0.315	0.120	0.0097	71,638	Pattaro *et al*. (2016)[8]
STC1	rs10109414	8	23,751,151	T	C	-0.605	0.116	$2.5x10^{-7}$	71,637	Kottgen *et al*. (2010)[4]
PIP5K1B	rs4744712	9	71,434,707	A	C	-0.753	0.112	$3.3x10^{-11}$	71,638	Kottgen *et al*. (2010)[4]

WDR37	rs10794720	10	1,156,165	T	C	-0.664	0.182	0.00033	71,638	Kottgen *et al*. (2010)[4]
A1CF	rs10994860	10	52,645,424	C	T	-0.322	0.177	0.072	61,866	Pattaro *et al*. (2016)[8]
KCNQ1	rs163160	11	2,789,955	G	A	-0.557	0.148	0.00021	71,638	Pattaro *et al*. (2016)[8]
DCDC5-MPPED2	rs3925584	11	30,760,335	T	C	-0.647	0.121	$1.5x10^{-7}$	65,983	Pattaro *et al*. (2012)[5]
AP5B1	rs4014195	11	65,506,822	G	C	-0.289	0.122	0.021	71,638	Pattaro *et al*. (2016)[8]
SLC6A13	rs10774021	12	349,298	T	C	-0.477	0.112	$2.9x10^{-5}$	71,638	Kottgen *et al*. (2010)[4]
TSPAN9	rs10491967	12	3,368,093	A	G	-0.398	0.148	0.0080	61,867	Pattaro *et al*. (2016)[8]
PTPRO	rs7956634	12	15,321,194	T	C	-0.426	0.125	0.00076	71,638	Pattaro *et al*. (2016)[8]
INHBC	rs1106766	12	57,809,456	C	T	-0.233	0.137	0.095	71,637	Pattaro *et al*. (2016)[8]
DACH1	rs626277	13	72,347,696	A	C	-0.428	0.116	0.00027	71,638	Kottgen *et al*. (2010)[4]
INO80	rs2928148	15	41,401,550	G	A	-0.278	0.113	0.016	71,638	Pattaro *et al*. (2012)[5]
SPATA5L1-GATM	rs2453533	15	45,641,225	A	C	-0.849	0.124	$1.8x10^{-11}$	71,638	Kottgen *et al*. (2010)[4]
WDR72	rs491567	15	53,946,593	A	C	-0.639	0.120	$1.4x10^{-7}$	71,638	Kottgen *et al*. (2010)[4]
UBE2Q2	rs1394125	15	76,158,983	A	G	-0.442	0.126	0.00052	71,638	Kottgen *et al*. (2010)[4]
PDILT-UMOD	rs12917707	16	20,367,690	G	T	-1.050	0.169	$8.8x10^{-10}$	48,102	Kottgen *et al*. (2010)[4]
DPEP1	rs164748	16	89,708,292	G	C	-0.593	0.131	$8.4x10^{-6}$	71,637	Pattaro *et al*. (2016)[8]
SLC47A1	rs2453580	17	19,438,321	C	T	-0.314	0.126	0.014	71,638	Pattaro *et al*. (2012)[5]
CDK12	rs11078903	17	37,631,924	A	G	-0.564	0.125	$8.6x10^{-6}$	71,638	Pattaro *et al*. (2012)[5]
BCAS3	rs9895661	17	59,456,589	C	T	-1.003	0.132	$7.9x10^{-14}$	71,638	Kottgen *et al*. (2010)[4]
NFATC1	rs8091180	18	77,164,243	A	G	-0.415	0.131	0.0018	59,864	Pattaro *et al*. (2016)[8]
SLC7A9	rs12460786	19	20,977,663	T	C	-0.030	0.117	0.80	71,638	Kottgen *et al*. (2010)[4]
SIPA1L3	rs11666497	19	38,464,262	T	C	-0.119	0.157	0.46	71,638	Pattaro *et al*. (2016)[8]
TP53INP2	rs6088580	20	33,285,053	C	G	-0.192	0.112	0.091	71,638	Pattaro *et al*. (2016)[8]
BCAS1	rs17216707	20	52,732,362	T	C	-0.761	0.150	$5.7x10^{-7}$	71,638	Pattaro *et al*. (2016)[8]

Chr: chromosome. SE: standard error.

[a]Effect allele is eGFR decreasing allele.

Table S5. Ancestry-specific association summary statistics for eGFR for lead SNPs from the trans-ethnic meta-analysis of 71,638 individuals.

Locus	Lead SNP	Chr	Position (bp, b37)	Alleles		Ancestry group	Ancestry-specific association statistics				
				Effect	Other		EAF	Beta	SE	*p*-value	*N*
GCKR	rs1260326	2	27,730,940	C	T	AFA	0.84	-1.123	0.422	0.0078	8,224
						EAS	0.44	-0.637	0.262	0.015	23,536
						EUR	0.60	-0.830	0.158	2.0×10^{-7}	23,553
						HIS	0.66	-1.098	0.248	1.1×10^{-5}	16,325
ALMS1	rs7587577	2	73,832,786	C	T	AFA	0.48	-0.894	0.301	0.0029	8,224
						EAS	N/A	N/A	N/A	N/A	N/A
						EUR	0.76	-1.037	0.186	3.2×10^{-8}	23,553
						HIS	0.73	-0.813	0.262	0.0020	16,325
LRP2	rs57989581	2	170,194,459	C	A	AFA	0.96	-2.458	0.813	0.0025	8,224
						EAS	0.92	-1.065	0.461	0.021	23,536
						EUR	0.98	-3.084	0.667	4.4×10^{-6}	23,553
						HIS	0.98	-2.658	0.784	0.00074	16,325
CPS1	rs715	2	211,543,055	C	T	AFA	0.22	-1.180	0.386	0.022	8,224
						EAS	0.16	-1.406	0.373	0.00017	23,536
						EUR	0.32	-0.765	0.175	1.4×10^{-5}	23,553
						HIS	0.28	-0.729	0.259	0.0051	16,325
TFDP2	rs1511299	3	141,716,072	T	C	AFA	0.91	-1.214	0.523	0.022	8,224
						EAS	0.72	-0.584	0.286	0.041	23,536
						EUR	0.74	-0.746	0.177	3.0×10^{-5}	23,553
						HIS	0.83	-0.668	0.306	0.029	16,325
SHROOM3	rs52020545	4	77,414,988	T	C	AFA	0.26	-0.410	0.355	0.25	8,224
						EAS	0.16	-1.803	0.391	4.0×10^{-6}	23,536
						EUR	0.43	-1.116	0.161	6.4×10^{-12}	23,553
						HIS	0.36	-0.579	0.242	0.017	16,325
DAB2-C9	chr5:39404526:D	5	39,404,526	D	R	AFA	0.12	-1.086	0.375	0.0039	8,224
						EAS	N/A	N/A	N/A	N/A	N/A
						EUR	0.42	-0.721	0.160	7.8×10^{-6}	23,553
						HIS	0.38	-0.925	0.241	0.00013	16,325
SLC43A1	rs35716097	5	176,806,636	T	C	AFA	0.36	-0.734	0.393	0.062	8,224
						EAS	0.33	-1.905	0.324	4.2×10^{-9}	23,536
						EUR	0.30	-0.897	0.178	5.8×10^{-7}	23,553
						HIS	0.29	-1.169	0.268	1.4×10^{-5}	16,325
LOC100132354-VEGFA	rs881858	6	43,806,609	A	G	AFA	0.41	-0.335	0.318	0.29	8,224
						EAS	0.87	-0.807	0.434	0.063	23,536
						EUR	0.68	-0.632	0.175	0.00034	23,553
						HIS	0.71	-1.375	0.264	2.1×10^{-7}	16,325
SLC22A2	rs316009	6	160,675,764	C	T	AFA	0.91	-1.648	0.547	0.0026	8,224
						EAS	0.95	-1.777	0.569	0.0018	23,536
						EUR	0.90	-1.000	0.255	0.00010	23,553
						HIS	0.92	-1.123	0.437	0.010	16,325
UNCX	rs62435145	7	1,286,567	T	G	AFA	N/A	N/A	N/A	N/A	N/A
						EAS	0.32	-1.611	0.282	1.1×10^{-8}	23,536
						EUR	0.66	-0.773	0.197	0.00010	23,553
						HIS	0.49	-1.208	0.261	4.1×10^{-6}	12,776
PHTF2	rs848486	7	77,552,127	G	A	AFA	0.53	-0.665	0.298	0.026	8,224
						EAS	0.23	-0.442	0.309	0.15	23,536
						EUR	0.41	-0.600	0.160	0.00020	23,553
						HIS	0.37	-0.844	0.238	0.00041	16,325
PRKAG2	rs10265221	7	151,414,329	C	T	AFA	0.16	-1.512	0.506	0.0028	8,224
						EAS	0.07	-0.680	0.707	0.34	23,536
						EUR	0.29	-0.850	0.183	4.2×10^{-6}	23,553
						HIS	0.20	-1.117	0.295	0.00016	16,325

PIP5K1B	rs4744712	9	71,434,707	A	C	AFA	0.42	-0.828	0.303	0.0063	8,224
						EAS	0.38	-0.620	0.264	0.019	23,536
						EUR	0.40	-0.669	0.158	$2.7x10^{-5}$	23,553
						HIS	0.28	-1.055	0.261	$5.7x10^{-5}$	16,325
DCDC5-MPPED2	rs963837	11	30,749,090	T	C	AFA	0.85	-0.365	0.453	0.42	8,224
						EAS	0.65	-0.989	0.268	0.00023	23,536
						EUR	0.55	-0.572	0.158	0.00032	23,553
						HIS	0.60	0.79	0.239	0.0010	16,325
SPATA5L1-GATM	rs2486288	15	45,712,339	C	T	AFA	0.82	-1.057	0.405	0.0090	8,224
						EAS	0.94	-0.497	0.543	0.36	23,536
						EUR	0.38	-0.923	0.162	$1.5x10^{-8}$	23,553
						HIS	0.63	-0.801	0.255	0.0018	16,325
WDR72	rs1031755	15	53,951,435	A	C	AFA	0.82	-0.966	0.390	0.013	8,224
						EAS	0.60	-1.463	0.263	$2.8x10^{-8}$	23,536
						EUR	0.79	-0.963	0.193	$7.0x10^{-7}$	23,553
						HIS	0.70	-0.016	0.263	0.95	16,325
PDILT-UMOD	rs77924615	16	20,392,332	G	A	AFA	0.92	-0.446	0.651	0.49	8,224
						EAS	0.78	-1.589	0.314	$4.2x10^{-7}$	23,536
						EUR	0.80	-1.300	0.213	$1.5x10^{-9}$	23,553
						HIS	0.80	-0.779	0.289	0.0072	16,325
BCAS3	rs9895661	17	59,456,589	C	T	AFA	0.45	-0.569	0.301	0.059	8,224
						EAS	0.53	-1.491	0.309	$1.4x10^{-6}$	23,536
						EUR	0.20	-0.851	0.231	0.00026	23,553
						HIS	0.45	-1.148	0.243	$2.5x10^{-6}$	16,325
NFATC1	rs8096658	18	77,156,537	G	C	AFA	N/A	N/A	N/A	N/A	N/A
						EAS	0.29	-0.856	0.308	0.0054	23,536
						EUR	0.47	-0.632	0.196	0.0014	23,553
						HIS	0.43	-1.124	0.270	$3.4x10^{-5}$	12,776

Chr: chromosome. EAF: effect allele frequency. SE: standard error. AFA: African American. EAS: East Asian. EUR: European. HIS: Hispanic.

Table S6. Residual association signals for eGFR from the trans-ethnic meta-analysis of 71,638 individuals at each locus after adjusting for the lead SNP.

Locus	Index SNP[a]	Chr	Pos (bp, b37)	Alleles		Unconditional meta-analysis			Conditional meta-analysis		
				Effect[b]	Other	Beta	SE	*p*-value	Beta	SE	*p*-value
GCKR	rs113778329	2	27,896,643	G	A	-0.608	0.383	0.12	-1.332	0.447	0.0029
ALMS1	rs12998058	2	73,511,468	G	A	-0.125	0.172	0.47	-0.717	0.173	$3.3x10^{-5}$
LRP2	rs74648148	2	169,774,784	G	C	-0.742	0.336	0.029	-1.114	0.382	0.0035
CPS1	rs9917188	2	211,904,894	T	A	-0.552	0.249	0.029	-0.910	0.251	0.00028
TFDP2	rs58623354	3	141,550,696	T	G	-0.313	0.144	0.032	-0.548	0.145	0.00017
SHROOM3	rs62300863	4	77,399,651	C	T	-0.697	0.152	$6.4x10^{-6}$	-0.543	0.161	0.00075
DAB2-C9	rs117574694	5	39,762,051	G	T	1.183	0.337	0.00053	-1.113	0.330	0.00074
SLC34A1	rs72813176	5	176,709,333	A	G	0.145	0.248	0.57	-0.585	0.253	0.021
LOC100132354-VEGFA	rs111451988	6	43,566,036	G	A	-0.638	0.305	0.040	-1.060	0.308	0.00058
SLC22A2	rs2665355	6	160,837,368	G	C	0.120	0.109	0.28	-0.401	0.110	0.00026
UNCX	rs10282027	7	1,005,018	A	G	-0.747	0.316	0.020	-1.047	0.317	0.00095
PHTF2	rs151202634	7	77,811,782	G	A	-1.495	0.525	0.0050	-1.585	0.513	0.0020
PRKAG2	rs6464171	7	151,505,876	C	G	-0.099	0.127	0.44	-0.388	0.127	0.0023
PIP5K1B	rs75852340	9	71,164,514	C	G	-1.916	0.564	0.00081	-1.877	0.661	0.0046
DCDC5-MPPED2	rs1813133	11	31,243,672	C	T	-0.523	0.340	0.13	-1.191	0.344	0.00054
SPATA5L1-GATM	rs140661904	15	46,041,594	A	T	-1.567	0.441	0.00047	-1.551	0.536	0.0038
WDR72	rs1878189	15	53,786,594	C	G	-1.015	0.282	0.00040	-1.108	0.302	0.00025
PDILT-UMOD	rs9928757	16	20,352,863	G	C	-1.012	0.165	$1.5x10^{-9}$	-0.677	0.180	0.00018
BCAS3	rs79068244	17	59,217,958	C	T	1.542	0.389	$9.3x10^{-5}$	-1.629	0.380	$1.8x10^{-5}$
NFATC1	rs526317	18	77,546,641	A	G	-0.676	0.198	0.00077	-0.549	0.186	0.0032

Chr: chromosome. SE: standard error.

[a]Index SNP has strongest residual signal of association across the locus in trans-ethnic meta-analysis after adjusting for lead SNP.

[b]Effect allele is eGFR decreasing allele in conditional meta-analysis.

Table S7. Association summary statistics for CKD at lead eGFR SNPs from the trans-ethnic meta-analysis of up to 3,976 cases and 55,904 controls.

Locus	Lead eGFR SNP	Chr	Position (bp, b37)	Alleles		CKD association summary statistics			Sample size: cases/controls
				Effect[a]	Other	OR (95% CI)	*p*-value	Cochran's *Q* *p*-value	
GCKR	rs1260326	2	27,730,940	C	T	1.04 (0.99-1.09)	0.16	0.0047	3,976/55,904
ALMS1	rs7587577	2	73,832,786	C	T	1.17 (1.09-1.24)	3.1×10^{-6}	0.29	2,646/33,698
LRP2	rs57989581	2	170,194,459	C	A	1.10 (0.98-1.24)	0.11	0.29	3,976/55,904
CPS1	rs715	2	211,543,055	C	T	1.06 (1.00-1.12)	0.069	0.38	3,976/55,904
TFDP2	rs1511299	3	141,716,072	T	C	1.06 (1.00-1.12)	0.068	0.39	3,976/55,904
SHROOM3	rs5020545	4	77,414,988	T	C	1.05 (1.00-1.12)	0.064	0.22	3,976/55,904
DAB2-C9	chr5:39404526:D	5	39,404,526	D	R	1.09 (1.02-1.16)	0.0084	0.63	2,646/33,698
SLC34A1	rs35716097	5	176,806,636	T	C	1.10 (1.04-1.16)	0.0011	0.84	3,976/55,904
LOC100132354-VEGFA	rs881858	6	43,806,609	A	G	1.06 (1.00-1.12)	0.057	0.010	3,976/55,904
SLC22A2	rs316009	6	160,675,764	C	T	1.13 (1.03-1.24)	0.0089	0.52	3,976/55,904
UNCX	rs62435145	7	1,286,567	T	G	1.18 (1.11-1.25)	2.2×10^{-7}	0.27	2,599/45,508
PHTF2	rs848486	7	77,552,127	G	A	1.03 (0.98-1.08)	0.31	0.29	3,976/55,904
PRKAG2	rs10265221	7	151,414,329	C	T	1.09 (1.02-1.16)	0.023	0.43	3,976/55,904
PIP5K1B	rs4744712	9	71,434,707	A	C	1.06 (1.01-1.11)	0.016	0.78	3,976/55,904
DCDC5-MPPED2	rs963837	11	30,749,090	T	C	1.04 (0.99-1.10)	0.15	0.49	3,976/55,904
SPATA5L1-GATM	rs2486288	15	45,712,339	C	T	1.09 (1.03-1.16)	0.0049	0.098	3,976/55,904
WDR72	rs1031755	15	53,951,435	A	C	1.09 (1.03-1.15)	0.0033	0.99	3,976/55,904
PDILT-UMOD	rs77924615	16	20,392,332	G	A	1.18 (1.10-1.26)	4.0×10^{-6}	0.11	3,976/55,904
BCAS3	rs9895661	17	59,456,589	C	T	1.06 (1.01-1.12)	0.020	0.70	3,976/55,904
NFATC1	rs8096658	18	77,156,537	G	C	1.07 (1.00-1.14)	0.040	0.13	2,599/45,508

Chr: chromosome. OR: odds ratio. CI: confidence interval.
[a]Effect allele is eGFR decreasing allele.

Table S8. Association summary statistics for eGFR for lead SNPs in 3,961/9,197 type 1/2 diabetes cases, all of European ancestry, from the SUMMIT Consortium.

Locus	Lead SNP	Chr	Position (bp, b37)	Alleles		Type 1 diabetes cases			Type 2 diabetes cases			All diabetes cases combined			
				Effect[a]	Other	Beta (SE)	*p*-value	*N*	Beta (SE)	*p*-value	*N*	Beta (SE)	*p*-value	Cochran's *Q p*-value	*N*
GCKR	rs1260326	2	27,730,940	C	T	-0.768 (0.618)	0.21	3,961	-0.204 (0.543)	0.71	9,197	-0.450 (0.408)	0.27	0.49	13,158
ALMS1	rs7587577	2	73,832,786	C	T	-0.977 (0.746)	0.19	3,961	-1.472 (0.611)	0.016	9,197	-1.273 (0.473)	0.0071	0.61	13,158
LRP2	rs57989581	2	170,194,459	C	A	-8.085 (4.266)	0.058	1,313	-3.417 (2.127)	0.11	9,197	-4.346 (1.904)	0.022	0.33	10,510
CPS1	rs715	2	211,543,055	C	T	-0.292 (0.662)	0.66	3,961	0.517 (0.571)	0.37	9,197	0.172 (0.432)	0.69	0.35	13,158
TFDP2	rs1511299	3	141,716,072	T	C	0.019 (0.701)	0.98	3,961	-0.228 (0.585)	0.70	9,197	-0.126 (0.449)	0.78	0.79	13,158
SHROOM3	rs5020545	4	77,414,988	T	C	-1.051 (0.616)	0.088	3,961	-0.777 (0.522)	0.14	9,197	-0.892 (0.398)	0.025	0.73	13,158
DAB2-C9	chr5:39404526:D	5	39,404,526	D	R	-1.165 (0.615)	0.058	3,961	0.363 (0.532)	0.49	9,197	-0.291 (0.402)	0.47	0.060	13,158
SLC34A1	rs35716097	5	176,806,636	T	C	-0.876 (0.658)	0.18	3,961	-0.507 (0.584)	0.39	9,197	-0.669 (0.437)	0.13	0.68	13,158
LOC100132354-VEGFA	rs881858	6	43,806,609	A	G	-0.356 (0.656)	0.59	3,961	-0.809 (0.557)	0.15	9,197	-0.619 (0.424)	0.14	0.60	13,158
SLC22A2	rs316009	6	160,675,764	C	T	0.268 (1.075)	0.80	3,961	-1.241 (0.827)	0.13	9,197	-0.680 (0.655)	0.30	0.27	13,158
UNCX	rs62435145	7	1,286,567	T	G	-0.884 (0.645)	0.17	3,961	-0.341 (0.600)	0.57	9,197	-0.593 (0.440)	0.18	0.54	13,158
PHTF2	rs848486	7	77,552,127	G	A	0.076 (0.616)	0.90	3,961	-0.260 (0.531)	0.62	9,197	-0.117 (0.402)	0.77	0.68	13,158
PRKAG2	rs10265221	7	151,414,329	C	T	-1.273 (0.731)	0.082	3,961	-2.129 (0.604)	0.00043	9,197	-1.782 (0.466)	0.00013	0.37	13,158
PIP5K1B	rs4744712	9	71,434,707	A	C	-0.640 (1.003)	0.52	1,313	0.475 (0.536)	0.38	9,197	-0.227 (0.473)	0.63	0.33	10,510
DCDC5-MPPED2	rs963837	11	30,749,090	T	C	-0.336 (0.614)	0.58	3,961	-0.872 (0.543)	0.11	9,197	-0.637 (0.407)	0.12	0.51	13,158
SPATA5L1-GATM	rs2486288	15	45,712,339	C	T	-0.711 (0.617)	0.25	3,961	-1.159 (0.544)	0.033	9,197	-0.963 (0.408)	0.018	0.59	13,158
WDR72	rs1031755	15	53,951,435	A	C	1.758 (0.702)	0.012	3,961	-1.018 (0.627)	0.10	9,197	0.212 (0.468)	0.65	0.0032	13,158
PDILT-UMOD	rs77924615	16	20,392,332	G	A	-1.405 (0.760)	0.064	3,961	-2.915 (0.668)	1.3×10^{-5}	9,197	-2.256 (0.502)	6.9×10^{-6}	0.14	13,158
BCAS3	rs9895661	17	59,456,589	C	T	-0.943 (0.771)	0.22	3,961	-0.471 (0.723)	0.51	9,197	-0.692 (0.527)	0.19	0.66	13,158
NFATC1	rs8096658	18	77,156,537	G	C	-0.617 (0.630)	0.33	3,961	-2.235 (0.578)	0.00011	9,197	-1.495 (0.426)	0.00045	0.058	13,158

Chr: chromosome. SE: standard error.
[a]Effect allele is eGFR decreasing allele from trans-ethnic meta-analysis.

Table S9. Properties of 99% credible sets of variants at eGFR loci on the basis of trans-ethnic meta-analysis of 71,638 individuals.

Locus	Lead SNP	Chr	Position (bp, b37)	99% credible set		
				SNPs	Distance (bp)	Interval (bp, b37)
GCKR	rs1260326	2	27,730,940	3	11,664	27,730,940-27,742,603
ALMS1	rs7587577	2	73,832,786	159	278,238	73,622,663-73,900,900
LRP2	rs57989581	2	170,194,459	6	10,315	170,194,459-170,204,773
CPS1	rs715	2	211,543,055	9	40,636	211,540,507-211,581,142
TFDP2	rs1511299	3	141,716,072	123	221,865	141,637,438-141,859,302
SHROOM3	rs5020545	4	77,414,988	6	20,971	77,394,018-77,414,988
DAB2-C9	chr5:39404526:D	5	39,404,526	31	68,620	39,359,773-39,428,392
SLC34A1	rs35716097	5	176,806,636	2	562	176,806,636-176,807,197
LOC100132354-VEGFA	rs881858	6	43,806,609	16	14,135	43,804,808-43,818,942
SLC22A2	rs316009	6	160,675,764	99	126,912	160,631,670-160,758,581
UNCX	rs62435145	7	1,286,567	7	11,947	1,281,064-1,293,010
PHTF2	rs848486	7	77,552,127	180	478,315	77,112,367-77,590,681
PRKAG2	rs10265221	7	151,414,329	13	9,719	151,405,818-151,415,536
PIP5K1B	rs4744712	9	71,434,707	5	3,534	71,431,174-71,434,707
DCDC5-MPPED2	rs963837	11	30,749,090	4	27,925	30,749,090-30,777,014
SPATA5L1-GATM	rs2486288	15	45,712,339	49	114,098	45,614,502-45,728,599
WDR72	rs1031755	15	53,951,435	20	49,581	53,915,766-53,965,346
PDILT-UMOD	rs77924615	16	20,392,332	1	1	20,392,332-20,393,332
BCAS3	rs9895661	17	59,456,589	6	22,488	59,449,636-59,472,123
NFATC1	rs8096658	18	77,156,537	2	435	77,156,103-77,156,537

Chr: chromosome.

Table S10. Membership of 99% credible sets containing no more than five variants on the basis of MANTRA trans-ethnic fine-mapping analysis of 71,638 individuals.

Locus	Lead SNP	99% credible set										
		Variant	Chr	Position (bp, b37)	Effect allele[a]	Other allele	Beta	SE	*p*-value	*N*	$\log_{10}$BF	Posterior probability π_C
GCKR	rs1260326	rs1260326	2	27,730,940	C	T	-0.872	0.114	6.1×10^{-14}	71,638	12.23	0.938
		rs780094	2	27,741,237	C	T	-0.810	0.113	2.0×10^{-12}	71,638	10.59	0.021
		rs780093	2	27,742,603	C	T	-0.821	0.114	1.3×10^{-12}	71,638	10.84	0.038
SLC34A1	rs35716097	rs35716097	5	176,806,636	T	C	-1.097	0.127	2.2×10^{-17}	71,638	15.92	0.946
		rs12659266	5	176,807,197	T	C	-1.109	0.134	4.3×10^{-16}	71,638	14.60	0.045
PIP5K1B	rs4744712	rs7042786	9	71,431,174	A	T	-0.727	0.113	2.1×10^{-10}	71,637	8.36	0.117
		rs2039424	9	71,432,174	G	A	-0.689	0.113	2.0×10^{-9}	71,638	7.57	0.019
		rs1556751	9	71,433,212	G	A	-0.666	0.113	7.0×10^{-9}	71,638	6.78	0.003
		rs10746942	9	71,434,465	G	A	-0.688	0.114	2.5×10^{-9}	71,637	7.54	0.018
		rs4744712	9	71,434,707	A	C	-0.753	0.112	3.3×10^{-11}	71,638	9.21	0.835
DCDC5-MPPED2	rs963837	rs963837	11	30,749,090	T	C	-0.685	0.114	3.7×10^{-9}	71,638	7.37	0.920
		rs3925584	11	30,760,335	T	C	-0.647	0.121	1.5×10^{-7}	65,983	5.84	0.027
		rs10767873	11	30,768,678	C	T	-0.628	0.115	8.2×10^{-8}	71,638	6.04	0.043
		chr11:30777014:I	11	30,777,014	R	I	-0.656	0.130	6.4×10^{-7}	48,102	5.24	0.007
PDILT-UMOD	rs77924615	rs77924615	16	20,392,332	G	A	-1.185	0.147	1.7×10^{-15}	71,638	14.23	1.000
NFATC1	rs8096658	rs71359461	18	77,156,103	C	G	-0.786	0.146	1.2×10^{-7}	59,864	5.95	0.113
		rs8096658	18	77,156,537	G	C	-0.814	0.141	1.3×10^{-8}	59,864	6.84	0.876
		rs138901831	18	77,160,067	G	C	-0.827	0.169	1.5×10^{-6}	59,864	4.78	0.008

Chr: chromosome. SE: standard error.
[a]Effect allele is eGFR decreasing allele.

Table S11. Posterior probability of driving eGFR association signals across for each single nucleotide variant annotation.

Annotation[a]	Number of single nucleotide variants	Posterior probability of driving association signals	
		Total	Percentage
Missense	317	1.04	5.39
5' UTR	249	0.14	0.73
3' UTR	709	1.02	5.29
Downstream	2099	0.3	1.56
Upstream	2473	0.12	0.62
Intronic	32384	12.12	62.83
Intergenic	13354	2.83	14.67
Non-coding transcript	1135	0.97	5.03
Others	1661	0.75	3.89

[a]Annotations were prioritised by considering the most severe consequence of all those reported for each variant.

Table S12. Genomic annotations of regulatory chromatin state from 93 cell types, Dnase I hypersensitivity sites from 145 cell types (DHS), and chromatin immuno-precipitation binding sites for 165 proteins (TF ChIP-seq) that were predictive of posterior probability of driving eGFR association signals (*p*<0.00012, Bonferroni correction for 403 annotations).

Annotation	Description	Logistic regression model			fGWAS
		Effect	SE	*p*-value	Effect (95% CI)
HDAC8	TF ChIP-seq	4.695	0.614	1.1×10^{-14}	6.45 (4.21-8.40)
NFE2	TF ChIP-seq	4.676	0.618	1.9×10^{-14}	4.72 (1.68-6.50)
FOSL1	TF ChIP-seq	3.866	0.558	2.1×10^{-12}	4.40 (2.40-5.80)
RPTEC	Renal epithelial DHS	2.194	0.407	3.4×10^{-8}	3.37 (2.25-4.42)
HRCE	Renal epithelial DHS	2.135	0.436	4.7×10^{-7}	3.11 (1.96-4.17)
ATF3	TF ChIP-seq	3.010	0.648	1.7×10^{-6}	3.66 (1.64-5.01)
fKidney_renal_cortex_L	Fetal kidney DHS	1.847	0.430	8.8×10^{-6}	2.76 (1.62-3.82)
fKidney_L	Fetal kidney DHS	1.881	0.446	1.2×10^{-5}	3.22 (2.05-4.36)
fKidney_R	Fetal kidney DHS	1.986	0.475	1.4×10^{-5}	3.41 (2.25-4.60)
IRF4	TF ChIP-seq	3.069	0.749	2.1×10^{-5}	3.84 (0.95-5.40)
fIntestine_Lg	Fetal intestine DHS	2.088	0.512	2.3×10^{-5}	2.96 (1.70-4.03)
fKidney_renal_pelvis	Fetal kidney DHS	1.884	0.465	2.5×10^{-5}	3.30 (2.15-4.22)
fKidney_renal_pelvis_L	Fetal kidney DHS	1.932	0.489	3.9×10^{-5}	3.50 (2.35-4.64)
MAFK	TF ChIP-seq	2.375	0.603	4.1×10^{-5}	3.03 (1.06-4.29)
HRE	Renal epithelial DHS	1.903	0.501	7.4×10^{-5}	2.87 (1.56-3.97)
fSkin	Fetal skin DHS	1.956	0.523	9.2×10^{-5}	3.40 (2.22-4.45)

SE: standard error. CI: confidence interval.

Table S13. Variants with more than 80% posterior probability of driving eGFR association signals that overlap with enriched regulatory annotations and their impact on expression of most correlated gene in GTEx database.

Locus	Lead SNP	Posterior probability π_C	Overlap with enriched regulatory annotations	Expression quantitative trait loci reported in GTEx database		
				Tissue	Gene	*p*-value
PDILT-UMOD	rs77924615	1.000	fKidney_R, RPTEC, fKidney_L, fKidney_renal_pelvis_L, fKidney_renal_pelvis, fKidney_renal_pelvis_R, HRCE			
SLC34A1	rs35716097	0.946	RPTEC, HRCE, HDAC8	Adipose_Subcutaneous	*RGS14*	4.1×10^{-15}
				Adrenal_Gland	*RGS14*	1.1×10^{-11}
				Artery_Aorta	*RGS14*	6.4×10^{-21}
				Artery_Coronary	*RGS14*	2.8×10^{-8}
				Artery_Tibial	*RGS14*	2.5×10^{-28}
				Brain_Cerebellum	*RGS14*	1.9×10^{-9}
				Breast_Mammary_Tissue	*RGS14*	1.1×10^{-8}
				Cells_Transformed_fibroblasts	*RGS14*	1.9×10^{-45}
				Colon_Sigmoid	*RGS14*	9.3×10^{-7}
				Colon_Transverse	*RGS14*	1.1×10^{-14}
				Esophagus_Mucosa	*RGS14*	5.2×10^{-18}
				Esophagus_Muscularis	*RGS14*	5.6×10^{-13}
				Heart_Atrial_Appendage	*RGS14*	3.9×10^{-13}
				Heart_Left_Ventricle	*RGS14*	1.4×10^{-18}
				Lung	*RGS14*	3.9×10^{-11}
				Muscle_Skeletal	*RGS14*	1.3×10^{-12}
				Nerve_Tibial	*RGS14*	6.1×10^{-15}
				Pancreas	*RGS14*	1.4×10^{-6}
				Pituitary	*RGS14*	3.0×10^{-13}
				Skin_Not_Sun_Exposed_Suprapubic	*RGS14*	2.1×10^{-8}
				Skin_Sun_Exposed_Lower_leg	*RGS14*	5.9×10^{-18}
				Stomach	*RGS14*	1.0×10^{-11}
				Testis	*RGS14*	1.5×10^{-27}
				Thyroid	*RGS14*	7.8×10^{-15}
DCDC5- MPPED2	rs963837	0.920	fKidney_R, fKidney_renal_pelvis_L			
NFATC1	rs8096658	0.877	fKidney_R, fKidney_L, fKidney_renal_pelvis, fKidney_renal_pelvis_R, fKidney_renal_pelvis_L	Heart_Left_Ventricle	*NFATC1*	2.4×10^{-9}
				Muscle_Skeletal	*NFATC1*	2.8×10^{-21}

PIP5K1B	rs4744712	0.835	fKidney_renal_pelvis	Artery_Aorta	*PIP5K1B*	$3.6x10^{-6}$
				Artery_Tibial	*PIP5K1B*	$3.6x10^{-14}$
				Testis	*PIP5K1B*	$1.9x10^{-6}$

Table S14. Estimated effects from the Cox proportional hazards model with robust standard errors, applied on the experimental *Drosophila melanogaster* survival data under isogenic and heterogenic conditions.

(a) Isogenic background

**Mutation**	**NaCl concentration**	**Log-hazard ratio**	**Robust SE**	***p*-value**
d06164	0.2	-1.4	0.32	2.2×10^{-5}
	0.3	-2.4	0.20	$<10^{-16}$
	0.4	-2.7	0.16	$<10^{-16}$
	0.5	-2.2	0.17	$<10^{-16}$
EY-P283	0.2	-0.29	0.75	0.21
	0.3	-1.9	0.22	$<10^{-16}$
	0.4	-2.3	0.17	$<10^{-16}$
	0.5	-1.7	0.18	$<10^{-16}$

(b) Heterogenic background

**Mutation**	**NaCl concentration**	**Log-hazard ratio**	**Robust SE**	***p*-value**
d06164	0.2	-2.1	0.46	6.9×10^{-6}
	0.3	-1.9	0.31	6.5×10^{-10}
	0.4	-1.8	0.22	1.1×10^{-16}
	0.5	-1.4	0.23	3.6×10^{-10}
EY-P283	0.2	-1.6	0.48	0.00078
	0.3	-2.3	0.32	2.0×10^{-12}
	0.4	-0.18	0.17	0.30
	0.5	-0.042	0.21	0.84

SE: standard error

ADDITIONAL ACKNOWLEDGEMENTS

AUSTWIN. We acknowledge funding from the Australian National Health and Medical Research Council (241944, 389875, 389891,389892, 389938, 442915, 442981, 496739 and 552485), US National Institutes of Health (AA07535, AA10248 and AA014041) and the Australian Research Council (DP0770096).

BBJ. The BioBank Japan Project was supported by the Ministry of Education, Culture, Sports, Sciences and Technology of the Japanese government.

HCHS/SOL. The Hispanic Community Health Study / Study of Latinos was carried out as a collaborative study supported by contracts from the National Heart, Lung, and Blood Institute (NHLBI) to the University of North Carolina (N01-HC65233), University of Miami (N01-HC65234), Albert Einstein College of Medicine (N01-HC65235), Northwestern University (N01-HC65236), and San Diego State University (N01-HC65237). The following contribute to the HCHS/SOL through a transfer of funds to the NHLBI: National Center on Minority Health and Health Disparities, the National Institute of Deafness and Other Communications Disorders, the National Institute of Dental and Craniofacial Research (NIDCR), the National Institute of Diabetes and Digestive and Kidney Diseases, the National Institute of Neurological Disorders and Stroke, and the Office of Dietary Supplements. The Genetic Analysis Center at the University of Washington was supported by NHLBI and NIDCR contracts (HHSN268201300005C AM03 and MOD03).

PIVUS and ULSAM. These projects were supported by Knut and Alice Wallenberg Foundation (Wallenberg Academy Fellow), European Research Council (ERC Starting Grant), Swedish Diabetes Foundation (2013-024), Swedish Research Council (2012-1397, 2012-1727, and 2012-2215), Marianne and Marcus Wallenberg Foundation, County Council of Dalarna, Dalarna University, and Swedish Heart-Lung Foundation (20120197). The computations were performed on resources provided by SNIC through Uppsala Multidisciplinary Center for Advanced Computational Science (UPPMAX) under Project b2011036. Genotyping was funded by the Wellcome Trust under award WT064890. Analysis of genetic data was funded by the Wellcome Trust under awards WT098017 and WT090532. We thank the SNP&SEQ Technology Platform in Uppsala (www.genotyping.se) for excellent genotyping.

WHI. The Women's Health Initiative program is funded by the National Heart, Lung, and Blood Institute, National Institutes of Health, US Department of Health and Human Services, through contracts HHSN268201100046C, HHSN268201100001C, HHSN268201100002C, HHSN268201100003C, HHSN268201100004C, and HHSN271201100004C.
